# Do Emotions Expressed Online Correlate with Actual Changes in Decision-Making?: The Case of Stock Day Traders

**DOI:** 10.1371/journal.pone.0144945

**Published:** 2016-01-14

**Authors:** Bin Liu, Ramesh Govindan, Brian Uzzi

**Affiliations:** 1 Google Inc, 1600 Amphitheater Parkway, Mountain View, California, United States of America; 2 University Southern California, Department of Computer Science, Los Angeles, California, United States of America; 3 Northwestern Institute on Complex Systems (NICO), Northwestern University, Evanston, Illinois, United States of America; University of Vermont, UNITED STATES

## Abstract

Emotions are increasingly inferred linguistically from online data with a goal of predicting off-line behavior. Yet, it is unknown whether emotions inferred linguistically from online communications correlate with actual changes in off-line activity. We analyzed all 886,000 trading decisions and 1,234,822 instant messages of 30 professional day traders over a continuous 2 year period. Linguistically inferring the traders’ emotional states from instant messages, we find that emotions expressed in online communications reflect the same distributions of emotions found in controlled experiments done on traders. Further, we find that expressed online emotions predict the profitability of actual trading behavior. Relative to their baselines, traders who expressed little emotion or traders that expressed high levels of emotion made relatively unprofitable trades. Conversely, traders expressing moderate levels of emotional activation made relatively profitable trades.

## Introduction

Emotional states inferred from online communications may furnish new approaches to collective behavior problems [1]. For example, if emotions predict how individuals are likely to perceive and process information, the content or timing of information may be tailored to emotional states to better achieve more positive outcomes. The great market crash of 1929, for example, is lamented by observers as striking without warning—the information on the day of the crash looked remarkably like any other day—one thing that differed on the day of the crash from other days was the emotional state of the actors [2]. Research on 9/11 dispatches suggests that the perception of the same information by different coordinators was partly contingent on their emotional state [3]. These patterns suggest that information presented to evacuees, persons in situations similar to the events on 9/11, or populations experiencing panics may be altered in various ways to improve the accuracy of the perceptions of critical information. Further, in the domain of consumer behavior, businesses may alter their messages about products, and in area of political affairs, governments can aim to improve voter engagement on climate change, gun rights, or other issues.

Other work finds that the emotional states of large populations are themselves changeable through online manipulation [4–5]. A large-scale online experiment involving millions of subjects showed that by manipulating the amount of positive or negative words a person received in their Facebook feeds caused them to express similar emotions in their feeds even though the experience that produced the emotion didn’t happen to them [4]. Consequently, not only can knowledge of someone’s mood potentially indicate how or when information might be presented to them to achieve a certain results, moods can be created or changed to guide collective actions.

Despite the potential of emotions to shape collective behavior, it remains to be tested whether emotions measured through online communications are associated with actual changes in off-line behavior. Classical approaches hold that emotions lead to more mental blunder than expected given the information at hand [6–8]. Conversely, recent neuro-physiological work suggests that emotions can reduce inhibitions that contributes to making risky choices [9–14]. Yet, in contrast to the strong evidence in experimental settings where emotions are verified physiologically, online settings measure emotions indirectly through linguistic analytics. As such, contacts online can repeat their friends’ words because of decorum or mimicry, rather than actually experiencing the emotion and its attendant behavioral manifestations [5]. Thus, it is unclear whether emotions measured in online communications predict off-line behavior in the way physiologically verified emotions do.

To investigate whether emotions expressed online relate to changes in behavior, we examined the relationship between stock traders’ emotions expressed online and their trading decisions. Many disciplines study the important links between emotions, trading behavior, and society [15–20]. Lab studies confirm that emotions do effect trading under controlled conditions [16], backing up observations by business leaders. “Success in investing,” Warren Buffet has remarked, “doesn't correlate with IQ once you are above the level of 100…what you need is the temperament to control the urges that get other people into trouble in investing.” Observational studies of traders have focused on the need to understand the range and regulation of emotions during trading. Top traders appear to find the right balance between emotions that support making quick, risky decisions rather than exhibit emotions that overwhelm rational evaluations of information [15]. Nevertheless, despite the interest of scientists and practitioners in the question of the link between online and offline behavior, difficult to acquire data has made real-world studies of trading behavior under different emotional states hard to conduct. To examine the link between expressed emotions online and trading outcomes, we designed a study that uses unique data on the instant messages and trades of real-world traders at a typical firm over a two year period. If emotions expressed online are proxies for real emotions they should predict changes in trading behavior not unlike the experimental evidence produced under control conditions.

## Methods

This is a Northwestern University IRB approved study (# STU00200578). All data were previously collected, accessed through the firm's archives, anonymized, involved no manipulation or interaction with subjects, and all subjects were fully aware that the data were being collected and could be used for research purposes. We received written permission from the firm to use these data, contingent on the identifying characteristics of the firm and its traders remaining confidential and anonymous.

## Data

Day traders exploit intraday movements in stock prices (i.e., day trades). They do not hold stocks long-term. Rather, to generate profits they make many, relatively small trades over the course of the day in an attempt to quickly enter and leave a position before other market participants react. To avoid unexpected price movements and reduce exposure that can occur overnight, they close all their positions at the end of the day, and then open new positions the next day. Hence, their behavior is normally well-bounded within a trading day. Data on our sample of day traders were collected unobtrusively in computer logs to avoid interfering with trading and included all known instant message communications (IMs) and trading decisions. All traders knew that the firm recorded all their communications and trades per SEC rules and that the data could be used for research purposes. We included all 30 professional stock day traders at the firm for which there were at least thirty consecutive days of complete data on their stock trades and communications, January 2007 through December 2008, which included 100,000s of trades and over 1,000,000 IMs [18]. All traders were men between 22 and 50 years of age, used the same trading technology, had access to the same public information news sources, and worked under equivalent compensation schemes [18]. Also at the firm, 14 traders were excluded from the analysis because they worked only a few days out of the two year period or sporadically, raising sample selection issues. The data for these traders is shown in Figure F in S1 File. Traders were paid a base salary plus commissions on trades, which were based on end of the day earnings rather than trade-by-trade earnings to smooth out fluctuations. These traders traded roughly half of the stocks available on the major U.S. exchanges and they tended to be stocks with higher intraday price fluctuations.

## Measures

The median daily profit of a trader is considered the relevant unit of analysis for measuring the quality of day traders’ decisions for several reasons. First, maximizing daily performance, not trade-by-trade performance, is a trader’s objective function. A trader might sell any stock at a loss to free up capital for a trade that provides a larger net gain for the portfolio. This means that an average measure of profit per trade is a better indicator of a trader’s central tendency than single trades. Second, traders make up to hundreds of decisions a day, which means that the average daily profitability helps account for random fluctuations in trade by trade behavior due to arbitrary distractions, accidently clicks, technical issues, or other chance factors. Third, prior research has validated this measure [15–16, 18–19]. Thus, we operationalized judgment in trading behavior as the median daily profit/loss of the trader. To aggregate individual trades to the average daily level of profit, we followed industry conventions that good trading involves finding the right buy and sell price within narrow windows of time [15–16]. We computed the per share difference in price between the actual price traded and the best possible price for a trade in the 5-minute window of time around the trade multiplied by the number of shares traded. For example, suppose a subject bought Google stock at $266.88 per share at 10:31 and Google stock prices at 10:30 ~ 10:35 were between $265.11 and $268.99 a share. The subject’s deviation is the actual price ($266.88) and “best” price ($265.11) multiplied by the number of shares (and vice versa for sell decisions). To test the validity of this operationalization of trading behavior, we verified that a trader’s choices of trading times within a 5-minute window could not be explained by randomness (*p* < 0.00001); the more traders deviate from the best possible times on a per trade basis, the lower the their daily profit (*p* = 0.005); and other measures of average daily profit (e.g., median) produced similar results as reported in S1 File.

**Fig 1:**
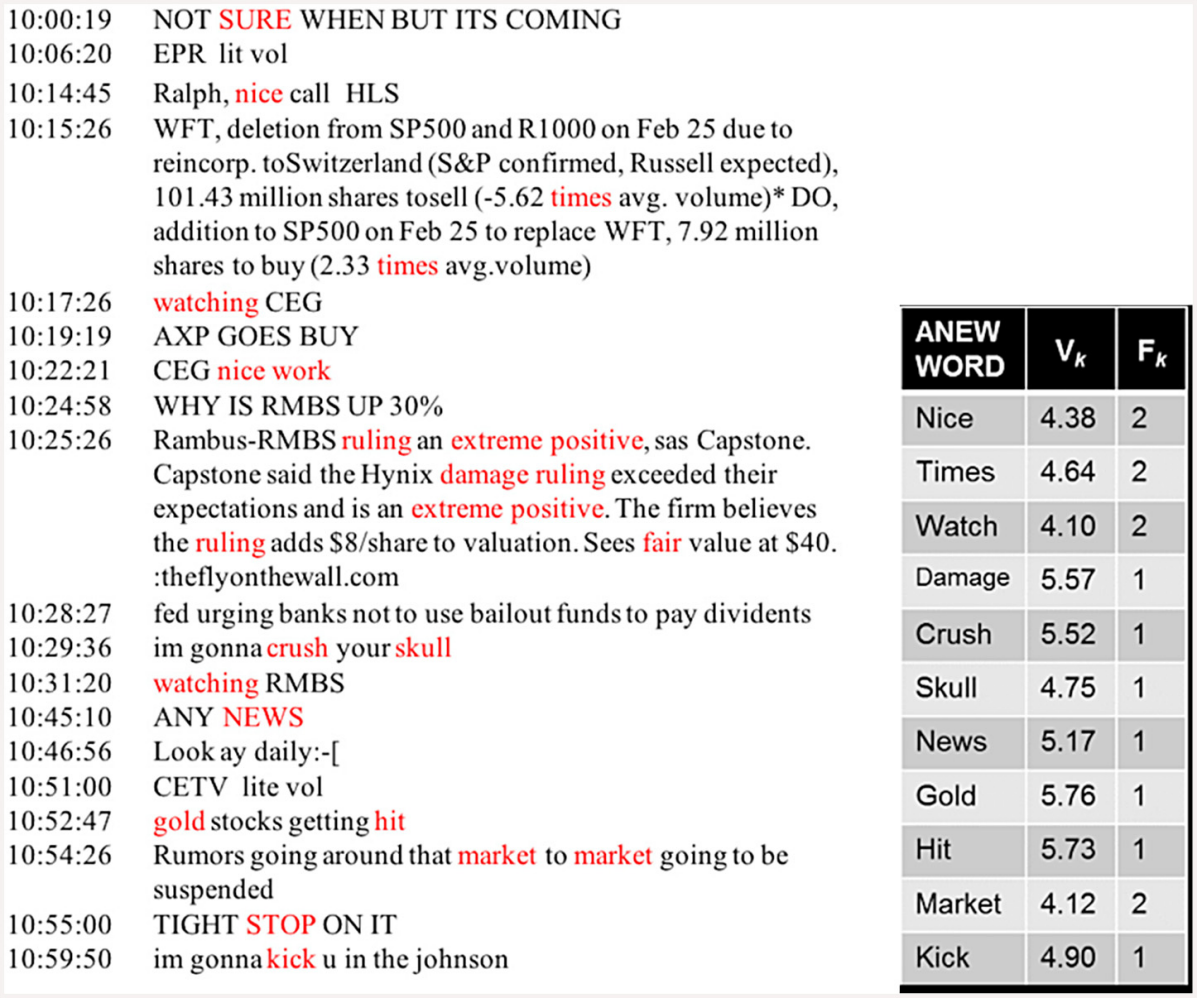
Instant Message Emotional Activation Coding. Left panel contains an actual Instant Message (IM) conversation as recorded in our data for a trader. Words highlighted in red are words in the ANEW dictionary that reflect a level of emotional activation over our threshold. Right panel indicates how an emotional activation level is computed for a selection of words that appear in the instant message conversation and the ANEW dictionary.

To code each trader’s IMs for emotional activation, we used the validated Affective Norms for English Words (ANEW) dictionary [21–22]. The ANEW dictionary is not the only way to measure emotional activation but it has been used in many previous studies [22–23]. The ANEW dictionary identifies words that when used in written or spoken conversation reflect a subject’s level of emotional activation. Each word is measured on a scale of 0.0, words expressing no emotional activation, to 9.0, words expressing high emotional activation, respectively. Following typical coding procedures, words in traders’ sent IMs were assigned to corresponding ANEW values. IMs with no ANEW words in them were assigned a value of zero activation, and IMs with one or more ANEW words in them were given the mean value of their ANEW words. Fig 1 exemplifies the coding of actual IMs as recorded in our data. Figure A in S1 File reports the frequency of the top 200 ANEW words appearing the traders’ Instant Messages. Table C in S1 File contains the words in the entire 2010 ANEW dictionary.

Once a value of activation was determined for an IM, we followed past research and converted continuous activation scores (0.0–9.0) to binary variables that defined whether the IM expressed activation or not [24]. Because no theory specifies an exact threshold at which to dichotomize an IM, we used three thresholds around the middle of the ANEW dictionary scale—4.5, 5.0, and 5.5 (S1 File). Further, Fig 1 shows that IMs with no activation and IMs with activation happen relatively close in time. Given that emotionally activated states may be in existence if expressed in some but not all communications happening close in time, or exist past the time of the last recorded IM because emotions decay over time [25–26], we did not attempt to pinpoint emotions at an exact time during the day. This would impose an unrealistic level of precision on the timing of emotions [25–26]. To manage this measurement issue regarding the precise timing over which emotions may affect behavior, [5] used the daily level of emotions expressed in Facebook posts as a proxy for the average level of emotion over the day. [4] used the daily expression of emotions in Facebook posts aggregated to the week across control and experimental conditions. Following prior research, we estimated a subject’s average daily level of activation as the frequency count of the number of daily sent IMs coded as activated controlling for total IMs exchanged. The following validation checks of this coding scheme were conducted. (1) We constructed a series to null models to see if the level of activated messages per day could be explained by chance by randomizing the sent IMs of each trader over a week to the days in the week, holding the number and hour of sent IMs per day constant. This test indicated that the frequency of activated IMs per day could not be attributed to chance (*p* < .0001). (2) We tested whether the words traders sent in IMs were composed of words from their receiving IMs, which if the case, might indicate that the words in the IMs were not self-expressions of their emotions but imitations of the emotions of their contacts. The overlap of activated language between received and sent IMs was less than 1% of on average, indicating that sent IMs contained original vocabularies of activated words. Thus, our coding scheme has precedent in prior research [4–5], links the average daily trading profit to average daily emotional activation, and is confirmed by validity checks within our context.

## Model

We used a within-subjects design to control for unobservable individual differences in the traders. The within-subject design makes each subject their own control group by controlling for fixed individual level differences that might affect trading behavior but are unobservable such as trader’s IQ, risk aversion, emotional baseline, or trading strategy net of exogenous control variables that can affect trading. Also, to estimate average effects, we ran fixed effects regressions with robust standard errors clustered by each trader and control variables [27–30] for (1) daily price volatility measured by the VIX, the standard volatility index used in finance_*t*_, (2) the sentiment of a trader’s newsfeeds_*t*_, (3) the sentiment of a trader’s received IMs_*t*_, (4) the information complexity of a trader’s newsfeeds_*t*_, (5) the information complexity of a trader’s received IMs_*t*_, (6) daily profit of a trader_*t*_, (7) prior daily day profit of a trader_*t-1*_, (8) number of traders at firm_*t*_, (9) daily number of instant messages exchanged by trader_*t*_, and (10) fixed effects for day of the week. The coding scheme for defining the information complexity and sentiment of the traders’ instant messages is reported in Tables A and B in S1 File.

## Results

**Fig 2:**
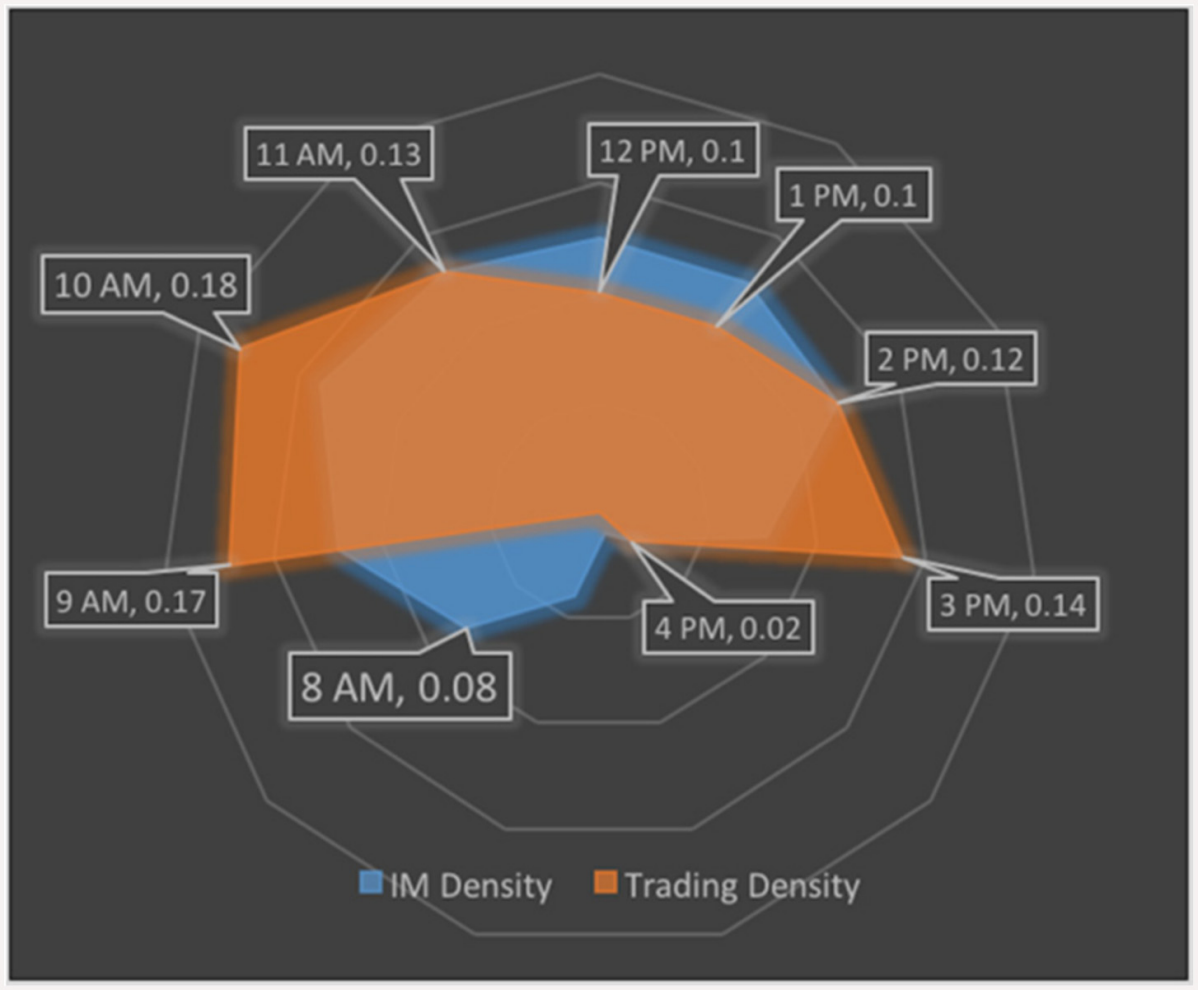
Instant Messaging and Trading are Closely Associated in Time. The figure shows the probability of observing traders trading (red area) and instant messaging (blue area) over the hours of the day averaged over time and traders in our sample and indicates that changes in the frequency of instant messaging are associated with changes in the frequency of trading. Trading begins at 9am and ends at 4pm. Instant messaging done at any time day or night is captured by the firm’s recording system.

Fig 2 shows that the frequency of use of IMs over the day. We note that the use of instant messaging grows quickly at the beginning of the day when day traders are taking new positions, drops around lunch, rises in mid-afternoon, and drops at day’s end when traders exit their positions. Also, we find that increases and decreases in the frequency of IM correlates with increases and decreases in trading frequency throughout the day, suggesting a systematic association between these variables.

**Fig 3:**
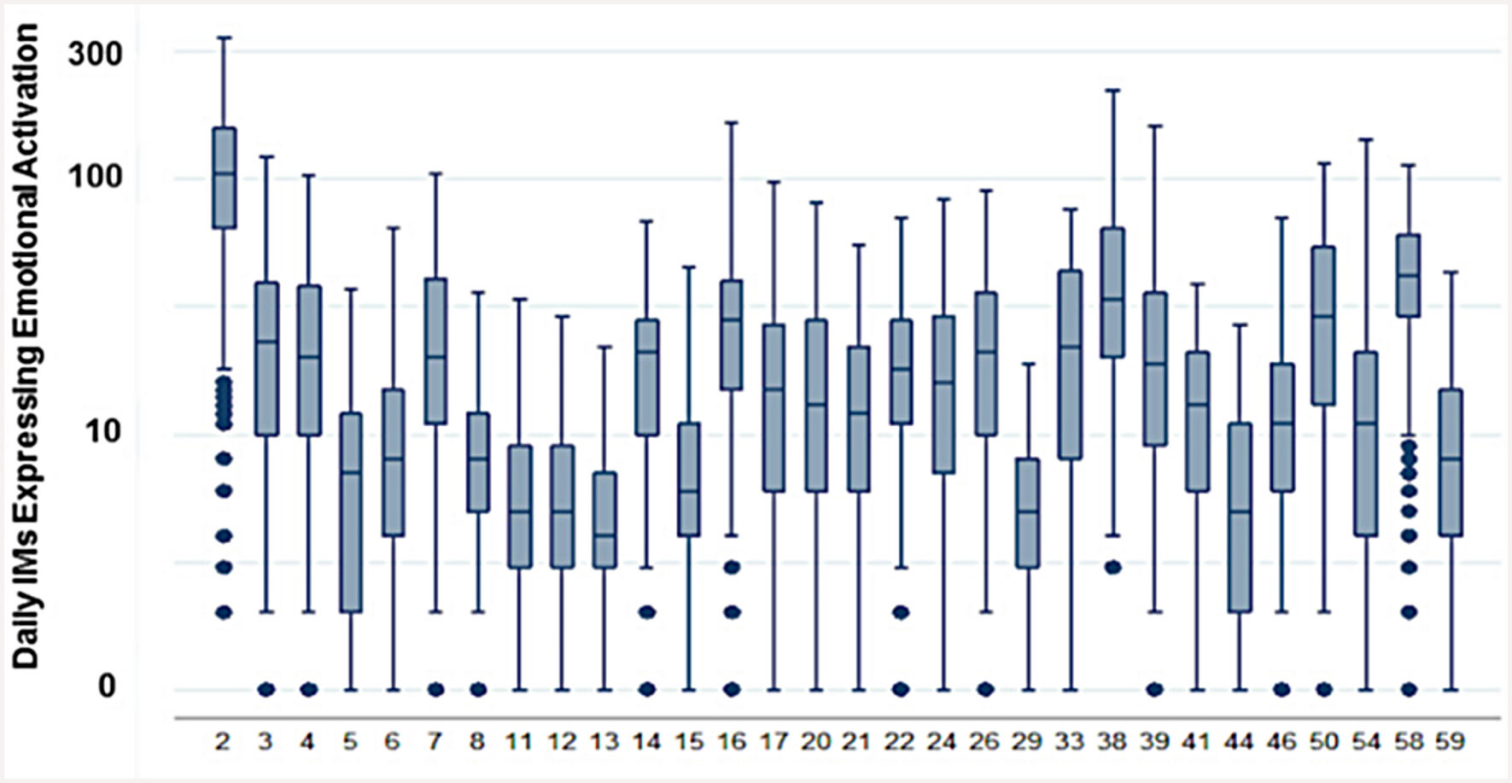
Linguistically Inferred Emotional Activation Levels. Each boxplot represents a trader’s distribution of daily instant messages expressing emotional activation as inferred from the linguistic content of their sent instant messages. Traders’ anonymous IDs are identified by the numbers on the x axis. Comparably distributions of emotional activation have been identified in traders from physiological measurements [16].

Experimental work normally manipulates one instantiation of emotional activation [15–16]. Little is known about the experimental realism of controlled experiments in real-world settings where actors can receive repeated exposures, learn, or be exposed to multiple determinants of behavior. Fig 3 shows the distribution of emotional activation for our 30 traders measured as the daily average number of sent IMs that linguistically contained expressed emotions. We note that the median level of emotional activation varies across traders. Nevertheless, while each trader’s central tendency for emotional activation varies, the dispersion around the median has a similar interquartile range, a finding consistent with the observation that different traders exhibit wide propensities for different levels of emotional regulation [15–16].

**Fig 4:**
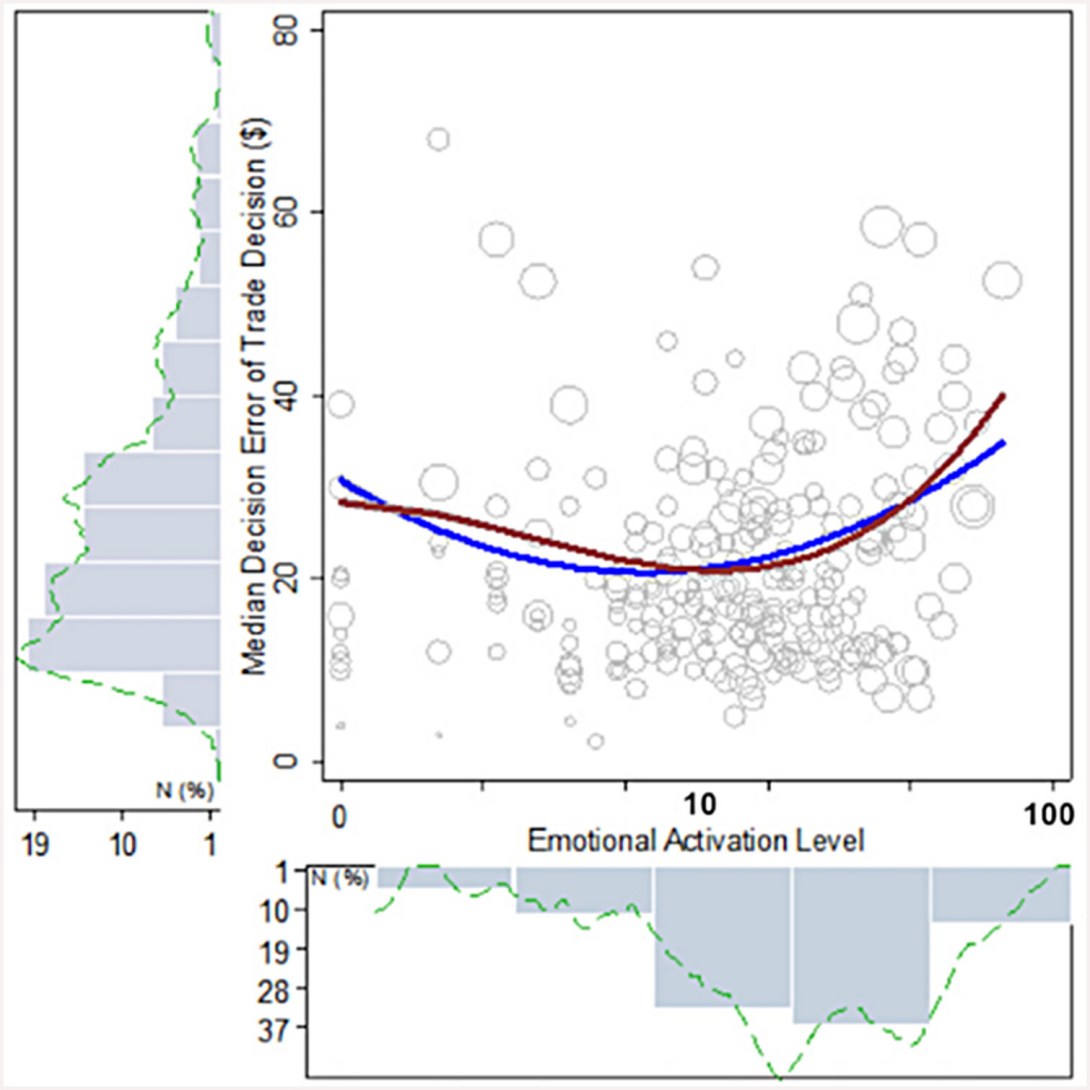
Daily Online Emotional Activation and Daily Median Decision Errors in Stock Trading Decisions. The figure shows the relationship between emotional activation and stock trading decision errors for trader #7. Size of scatter points represent the number of observations. The red line is a non-parametric lowess spline fit and the blue line is a quadratic fit. Plots for each trader are shown in the S1 File.

Fig 4 suggests that emotional activation expressed through online communications is predictive of changes in trading behavior. Considering trader #7 as a case example, the x-axis shows the association between his daily emotional activation and his median deviation from his best profit. The plot suggests that a medium level of activation is most associated with optimal trades. Movement in levels of activation away from a median level—towards a decrease in activation or an increase in activation—are associated with significantly greater deviations from optimal trading. To estimate the relationship of these associations more precisely, we used a non-parametric lowess spline (red line). The shape of the spline suggests that a U shaped relationship characterizes the association between emotions and trading. To test this expectation, we fitted a quadratic regression (blue line) that included control variables for price volatility, sentiment of traders’ sent messages, information complexity of the trader’s received instant messages and online newsfeeds, daily profit of trader, daily prior day profits, daily number of instant messages exchanged, and fixed effects for day of week [27–30]. A Wald test, residual plot, and the BIC statistics indicated that the quadratic specification fit the data better than a linear fit are reported in Figure B in S1 File. The predicted regression line (with error bars) estimate for this trader suggests that decision errors increase up to a median expected loss of 35 dollars per trade or $2,350 per day net of control variables. The same plot for each trader in the study is shown in Figures C and D and E in S1 File.

To estimate the group-level relationship for all 30 traders, Table 1 presents the results of a pooled fixed-effects regression with robust standard errors clustered on traders. Table 1 indicates that the overall model explains 67% of the variance in deviations from trading. Also, as expected, we find that the level of price volatility during the day of trading is significantly associated with the profitability of traders [29, 30]. For day traders, volatility is positively related to the level of decision making error. This suggests that as price movement uncertainty increases, traders are less able to anticipate the market, making poorer trading decisions on average. Similarly, we observe that the greater the complexity of news, the greater the level of decision errors. News with higher complexity would contain more components of information and/or more diverse information. Thus, while news is important to provide information to trade on, when the news is complex, it becomes process and assimilate quickly, making stock predictions less reliable [12, 20, 28]. Net of controls, we observe that on average traders exhibit a curvilinear relationship between their level of emotional activation and average level of decision errors. When traders are either lacking in emotional activation or highly emotionally activated they tend to make poorer trading decisions as measured by the amount of money they make on the trade. This empirical evidence is consistent with the conclusion that emotions measured online can be predictive of off-line behavior.

**Table 1:** Daily Median Errors in Stock Trading and Daily Emotional Activation as Measured via Online Communications. Fixed effect regression showing the relationship between changes in the daily emotional activation of day traders and the size of their trading decision errors as measured by the deviation in trading profits from the optimal trades for the day that could have taken place within the same 5 minute window as the actual trade that did take place. Results suggest an inverted U shaped relationship between the magnitude of decision errors and the level of emotional activation of the decision maker. Consistent with prior work, the more IMs exchanged by a trader the better their trading decisions [18–19]. Robust standard errors clustered on trader ID showing are shown. SI File presents null models, robustness checks, and detailed explanations of control variables.

VARIABLES	Coefficient	Robust SE
Emotional Activation _*t*_	-1.260**	-0.518
Emotional Activation^2^_*t*_	0.303***	-0.101
Price Volatility _*t*_	0.0685***	-0.0208
Profit of Trader _*t-1*_	-6.55E-07	-1.15E-05
# of IMs Trader Exchanged _*t*_	-0.0588**	-0.0265
Information Complexity—Instant Messages _*t*_	-3.967	-3.732
Information Complexity—News _*t*_	0.0334**	-0.0138
Sentiment—Instant Messages _*t*_	0.214	-0.146
Sentiment—News _*t*_	-0.000398	-0.000358
Crash Fixed Effect	Yes	Yes
Day of Year Fixed Effect	Yes	Yes
Day of Week Fixed Effects	Yes	Yes
Trader Fixed Effects	Yes	Yes
Constant	23.28**	-8.645
R-squared	0.67	

*** p<0.01,

** p<0.05,

* p<0.1.

## Discussion

Experimental research shows that otherwise normal subjects with localized brain damage that prevents them from becoming emotionally activated, hesitate to make risky decisions despite having made the same agile and rational calculations made by normal subjects. In the case of risky decisions like stock trading, hesitation is likely to hurt a trader’s ability to trade at the right time. Conversely, over-activation has been found to derail rational faculties as well. Facing risky choices about investments, emotionally activated subjects accept worse offers and make bigger blunders than control groups. Thus, evidence from this sample of traders is consistent with the idea that emotions measured linguistically using online communications predicts behavior in a not dissimilar way to emotions measured physiologically in regard to risk decision-making [15–16].

Our study suggests while information complexity is an important determinant of decision acumen, online expressions of emotional activation may help identify conditions under which systems could deviate from desired states after taking into account the quality of the information in the system. We show that these patterns can be predicted via unobtrusive digital measurements provided increasingly by online social network communications. Furthermore, our analysis implies that even in systems designed to have high quality information, decision-makers may make poor decisions when not in the right state of mind. Many critical emergency systems are designed to have optimal and timely amounts of information available to decision-makers. Emergency evacuation systems, for example, provide text messages with information about where and when to move to a safe location. Markets are ever searching for better measures of information pertinent for efficient trading and high return investments. 911 dispatchers are trained to provide the best lifesaving information.

This work implies that in these systems where quality of information and the state of mind of the decision-makers is critical to system effectiveness, large-scale and pervasive network communication can be used to estimate the state of mind of the decision-makers and provide early warning signals for systems at risk of failure. For example, the tracking of IMs or Tweets during an evacuation or natural disaster could indicate which targets of aid are least likely to make good decisions. Similarly, in organizations with analysts who must interpret data quickly and make rapid decisions—such as financial firms, national security agencies, or emergency rescue workers—the ability to evaluate their states of mind via their communications over the course of the event could be used to determine when operators should step away from their decisions or become more engaged. As information continues to grow in volume and in number of potential sources, future work should address how the collective intelligence of systems can be improved in terms of how to best present and process data for risky decisions.

## Supporting Information

S1 File Supporting Analyses, Results and Null models. (PDF)
