# Supplementary material for: Do Emotions Expressed Online Correlate with Actual Changes in Decision-Making?: The Case of Stock Day Traders
Source: PLoS One. 2016 Jan 14;11(1):e0144945. doi: 10.1371/journal.pone.0144945 (PMC4713085; doi:10.1371/journal.pone.0144945)
Supplement: S1 File — (PDF) [file pone.0144945.s001.pdf]

S1 File. Supporting Analyses, Results and Null models

Do Emotions Expressed Online Correlate with Actual Changes in Decision-Making and Judgment?: The Case of Stock Day Traders

Bin Liu[1]
Ramesh Govindan[2]
Brian Uzzi[3*]

[1]Google Inc.
[2]University Southern California
[3*]Northwestern Institute on Complex Systems (NICO) and Northwestern University
* Corresponding author

MEASUREMENT AND ROBUSTNESS CHECKS

Emotional Activation

To code words in texts we prepped each text by (i) reducing each word to its stem word (Porter 1980) (e.g., connecting or connection = connect); (ii) converting abbreviations to words (e.g., lov and luv to love); (iii) coding words in the text for activation using the ANEW dictionary's standard word scale of 0.0 to 9.0. The Affective Norms for English Words (ANEW) dictionary contains English words, which, when used in conversation, provides a proxy for a person's level of emotional activation. The ANEW was validated using autonomic physiological responses (pulse, conductance, etc.) and is used extensively across research domains (e.g., the search term "The Affective Norms for English Words" generates more than 100,000 Google hits). Following prior work on establishing a level at which activation is considered to occur for an individual, we followed prior work (e.g., Kassam et al. 2013) and defined a person as being in an activated state (1=yes; 0=otherwise) if their average activation level exceeded a threshold level of activation based on the activation words appearing in the text. Because there is no a priori theory specifying an exact threshold, we addressed the issue empirically and used three thresholds. The three thresholds were 4.5, 5.0, or 5.5.

Null Models

To test that activation effects are not due to chance, we constructed null models that randomized the ordering of texts over the hour, day, week, or month. These tests verified that chance did not explain observed periods of activation ($p < .0001$).

Sensitivity Tests for Different Thresholds of Activation

To test that activation effects are robust to a chosen threshold and length of duration of activation, we used three threshold values 4.5, 5.0, and 5.5 and 7 durations 30, 35, 40, 45, 50, 55, and 60 minutes. All three thresholds produced a similar pattern of effects as noted above. According to a binomial test, the likelihood that 448/735 (=3*7*35) pass the two-tail binomial test has a p-value<.00001 as shown above in the regression analyses.

Counts of the Top 200 Activation Words used by All Traders

The S1A and S2B Figures show the frequency count of the appearance of ANEW activation words in traders' texts. In cases where traders use abbreviations and intentional misspelled words in their texts, e.g., plai for play or comput for compute, we report native spellings.

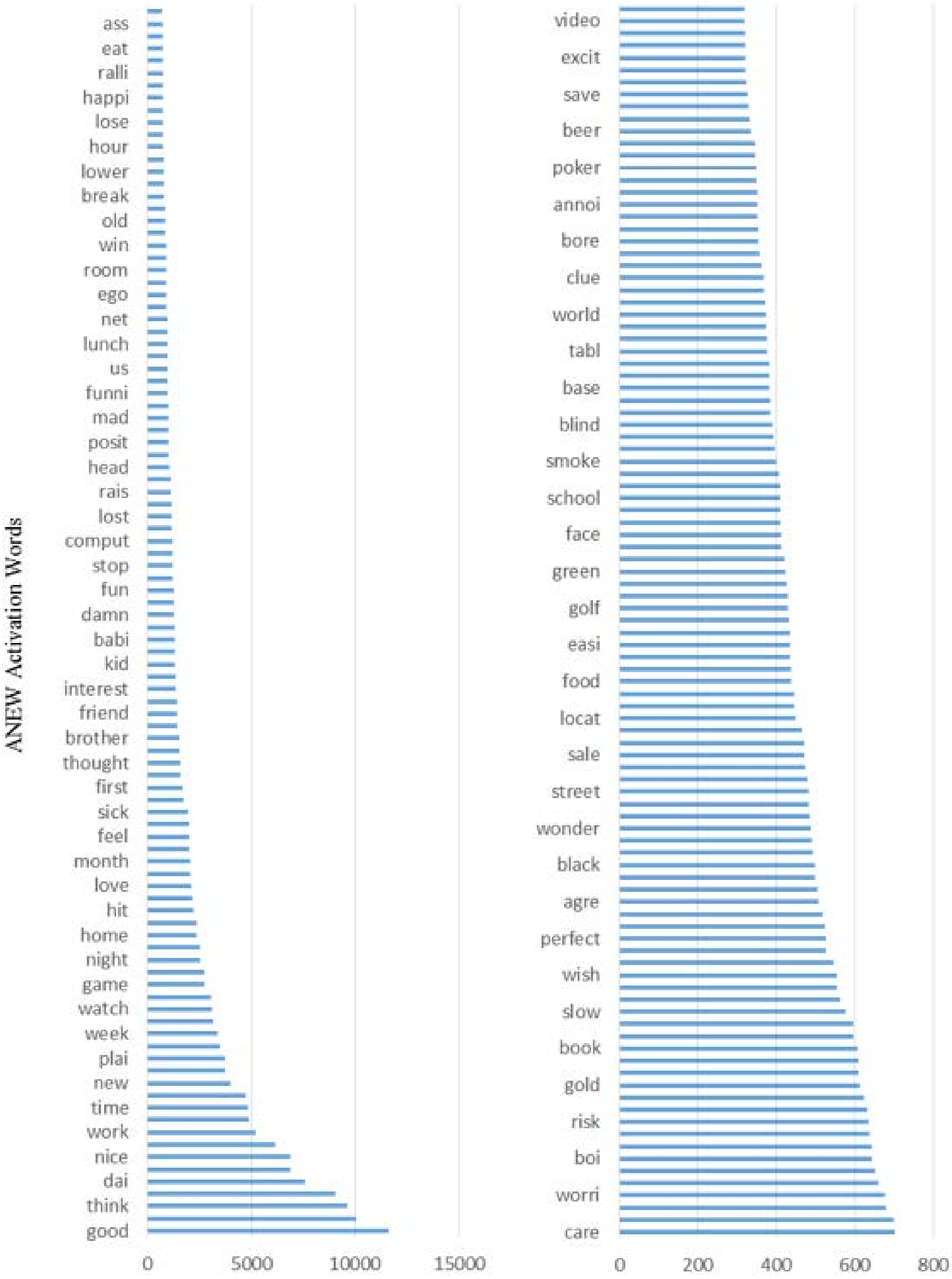

Figure A in S1 File. Counts of the Top 1-100 (left panel) and 101-200 (right panel) Activation Words Appearing in Traders' Instant Messages

Decision Error

The *relevant unit of analysis* for measuring decision error is the quality of trading decisions at the level of the day for several reasons. *First*, maximizing performance at the level of the day, not at the level of a trade, is the objective function of a trader. Traders are evaluated at the level of the day by managers and analysts. *Second*, traders make up to hundreds of decisions a day. Thus, a trade-by-trade analysis is not as informative as the average daily decision acumen. *Third*, prior research specifically on stock traders has validated this measure (Lo and Rapin 2002; Saavedra et al. 2011; Steenbarger 2002; Lo et al. 2005). Consistent with this logic, we analyzed the relationship between decision error and activation at the day level.

Decision Error is measured as the difference between the trade price and the hypothetical best price for that trade, scaled by the number of shares traded in that trading decision and averaged over a day to give the average deviation over decisions in a day. For example, suppose a subject bought Google stock at $266.88 per share at 10:31 and Google stock prices at 10:30 ~ 10:35 were between $265.11 and $268.99 a share. The subject's deviation is the actual price ($266.88) and "best" price ($265.11) multiplied by the number of shares and vice versa for sell decisions.

Null models of Random Decision Error

We randomly sampled 1,000 prices from each 5-minute window around a trading decision verified that chance could not explain the observed decision error ($p < 0.00001$).

Face Validity

Measure has *face validity:* decision error per trade is correlated with a trader's daily profit ($p =$ 0.005).

Agreement among Alternative Measures of Decision Error

Different measures of Decision Error produced similar patterns of findings. The (1) median, (2) arithmetic mean, (3) harmonic mean, (4) geometric mean, (5) grand mean-arithmetic mean, (6) grand mean – harmonic mean, (7) grand mean – geometric mean, and (8) z-score all produced similar results. To do these tests, we ran regressions that consisted of the 8 different measures of Decision Error and our 21 different threshold conditions for being activated times our 35 subjects. The threshold conditions used were: three threshold values 4.5, 5.0, and 5.5 for level activation and 7 emotional activation durations of 30, 35, 40, 45, 50, 55, and 60 minutes (explained in detail above in the section on emotional activation). This produced 8*3*7*35 = 5,880 regression coefficients for our 35 subjects. We found that 61.79% of the regressions coefficients were significant and in the predicted direction ($p<.00001$) (i.e., we ran separate a regression for each subject). This pattern of results is unlikely to occur by chance (binomial test, $p<.00001$). A fixed-effect regression pooling all the individual traders and dummy coding each trader separately in the regression confirmed the within-subject effects across the 8 different measures.

Regression Specification

The fixed-effects regressions estimate the effect for the “average” subject using a fixed effect for each trader, frequency weights for number of trades, robust standard errors and standard errors clustered by trader.

Dependent Variable
Decision Error (defined above)

Independent Variable
Emotional Activation (defined above)

Control variables

1. *Daily Volatility* measured by the VIX to control for market uncertainty,
2. *End of Day profit of the Trader at t-1* to control for risk aversion,
3. *Daily Total IMs sent* by the trader to control for IM volume,
4. *Informational complexity of trader’s IMs* to control for information in texts affecting decision making error,
5. *Informational complexity of news* to control for public information affecting decision making acumen,
6. *Sentiment of the trader’s IMs* to control for sentiment toward information in texts,
7. *Sentiment of news* to control for sentiment in public information
8. *Crash*
9. *Day of Year Fixed Effect* to control for *Daily Number of Active Traders* at the firm to control for organizational effects, profits for the day, and all other values that are fixed for a single day but can vary between days
10. *Day-of-Week Fixed Effect* to control for the possibilities that Mondays differ from Tuesdays because the markets reopened after the weekend close, Friday traders may go home early, etc.),
11. *Trader Fixed Effect*

Null model tests of the regression coefficients show they cannot be explained by chance.

We compared all the regression models for each subject with the observed instant messages randomized at the *week, month and year* level against those obtained from randomization. 13,055 out of 17,640 regressions (recall we have in total 5,880 regressions for decision errors, so the three permutation granularities produce 5,880*3 = 17,640 cases for null models), had coefficients that were different than expected by chance (z-score > 2 for Activation^2, and z-score < -2 for Activation) using this formula: Z-score = [observed β – mean(1,000 permutated βs)] ÷ [std (1,000 permutated βs)]. The difference observed between the actual and null models was unlikely to occur by chance ($p < .00001$) according to a binomial test.

Sensitivity Tests in Regressions for Activation Levels

For the individual and fixed effects regressions we ran a large range of regressions. The regressions had the same dependent variables, same independent variables and same control variables as noted above. The change from regression to regression depended on *threshold of activation* chosen. As discussed in the text, we had three thresholds, 4.5, 5.0, and 5.5. 60% of regressions had significant negative coefficients for emotional activation and a significant positive coefficient for emotional activation squared (the hypothesized relationships). According to a binomial test, the likelihood that 448/735 pass two-tail binomial test has a p-value<.00001.

Functional Form Analysis

Residual plots

Residual plots indicated that the relationship between activation and decision error was non-linear. The best fitting model was of the form Decision error = Activation + Activation^2 and was confirmed. The BIC of the linear equation was somewhat higher than the quadratic equations, which also indicated that the quadratic was a better fit

Wald Test

A *Wald test* of the quadratic fit vs. a similar simple linear model confirmed the fit of the quadratic model (p < .006).

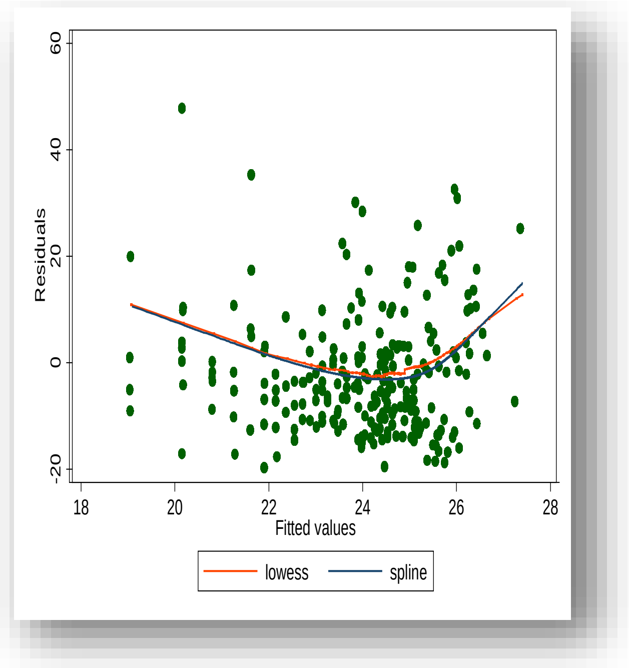

Figure B in S1 File. Residual Plot of the Residuals of a Regression of Decision Error on Activation.

Individual Trader Plots

Below we present the scatter plot of each trader's emotional activation and decision error relationship.

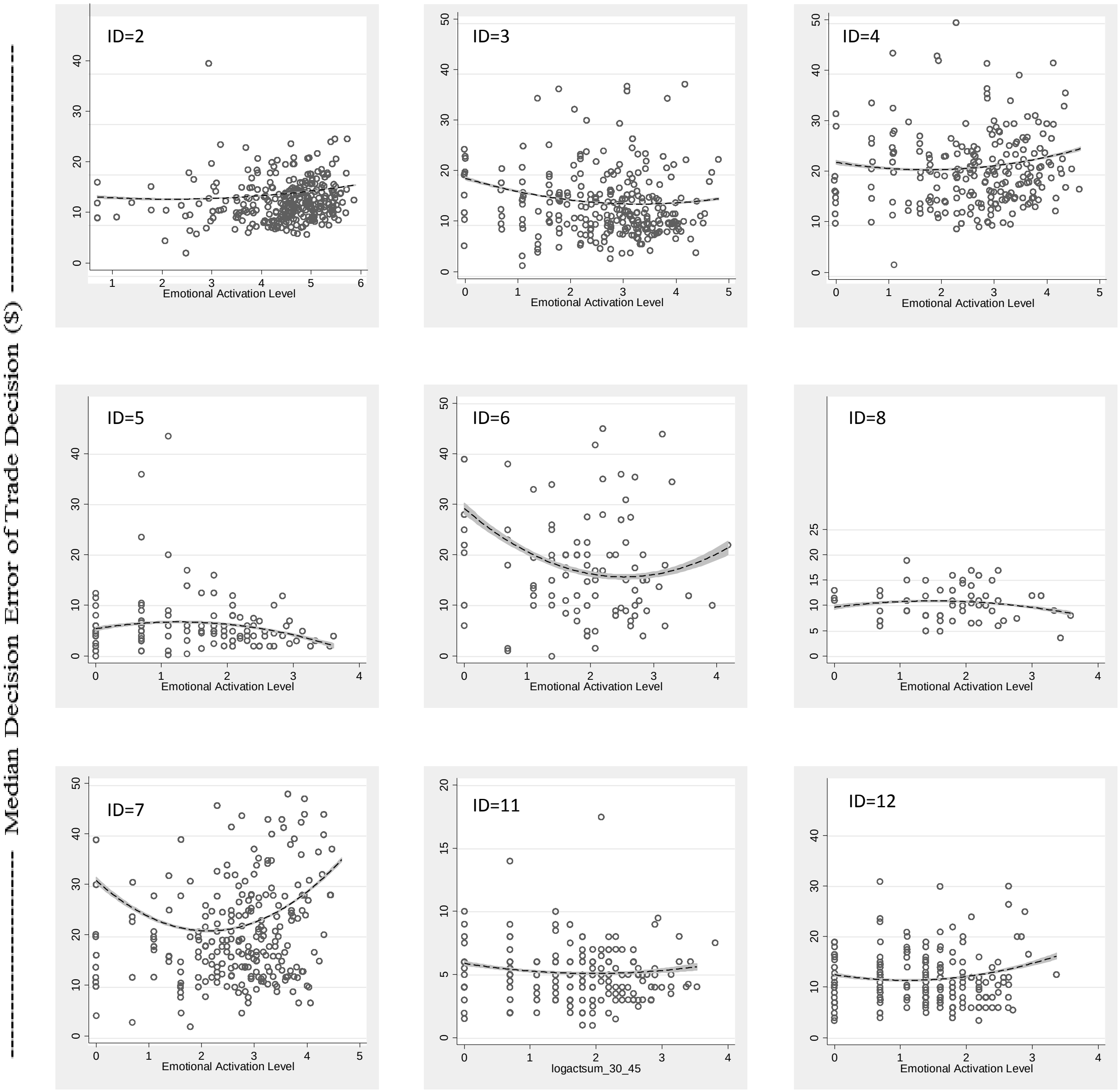

Figure C in S1 File. Scatter Plots of Each Trader's Decision Error and Emotional Activation Relationship. Below are the separate scatter plots for each trader in our study. Scatter plots bordered in red indicate that a U-shaped relationship between emotional activation and decision errors was statistically supported; the linear term was significant and negative ($p < .05$) and the quadratic term was significant and positive ($p < .05$). The ID number of each trader is indicated in the top left corner of each plot. A fixed-effects regression (Table 1) pooling all the traders together showed that the U-shaped relationship though not universal among traders holds on average for the traders net of control variables.

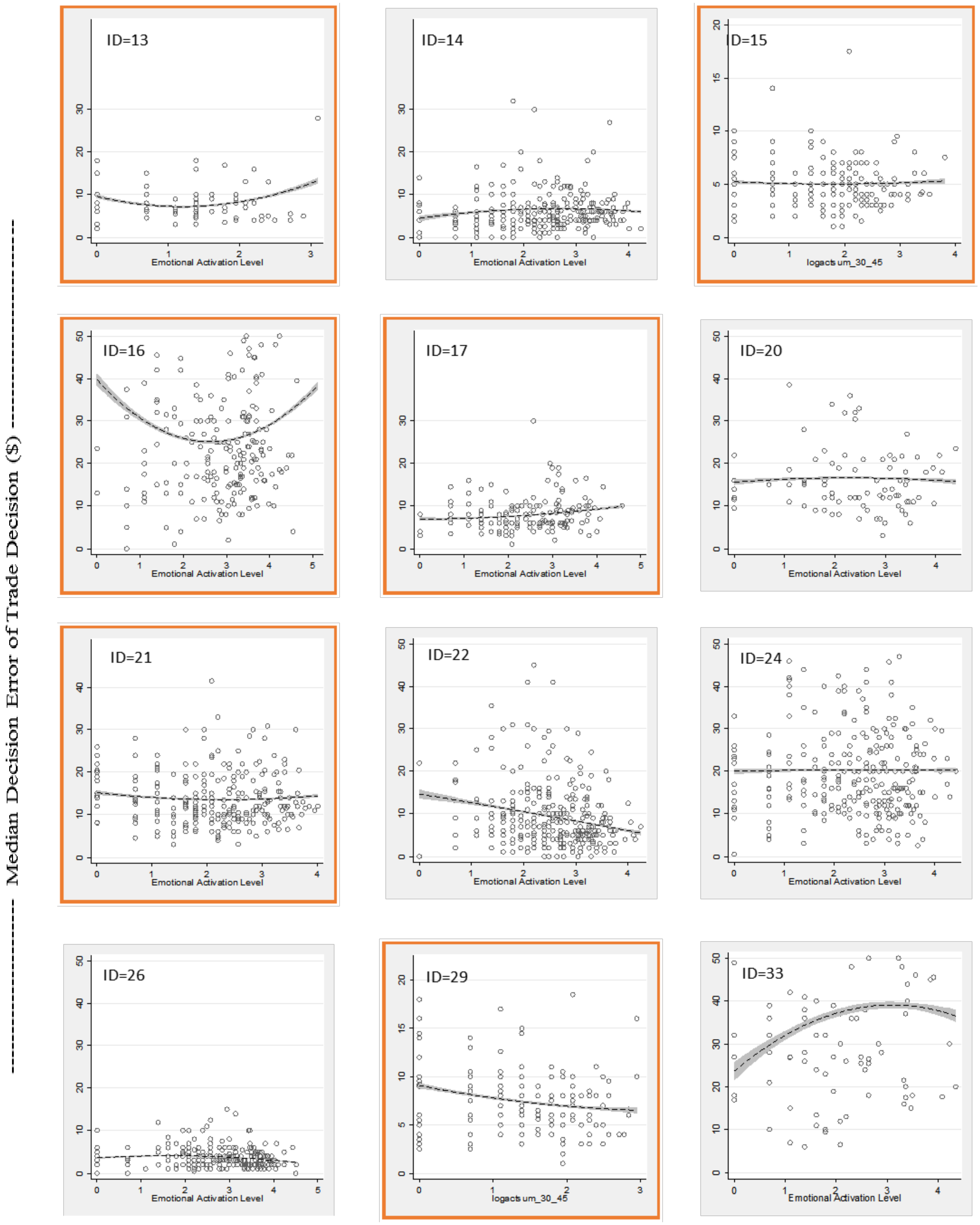

Figure D in S1 File. Scatter Plots of Each Trader's Decision Error and Emotional Activation Relationship.

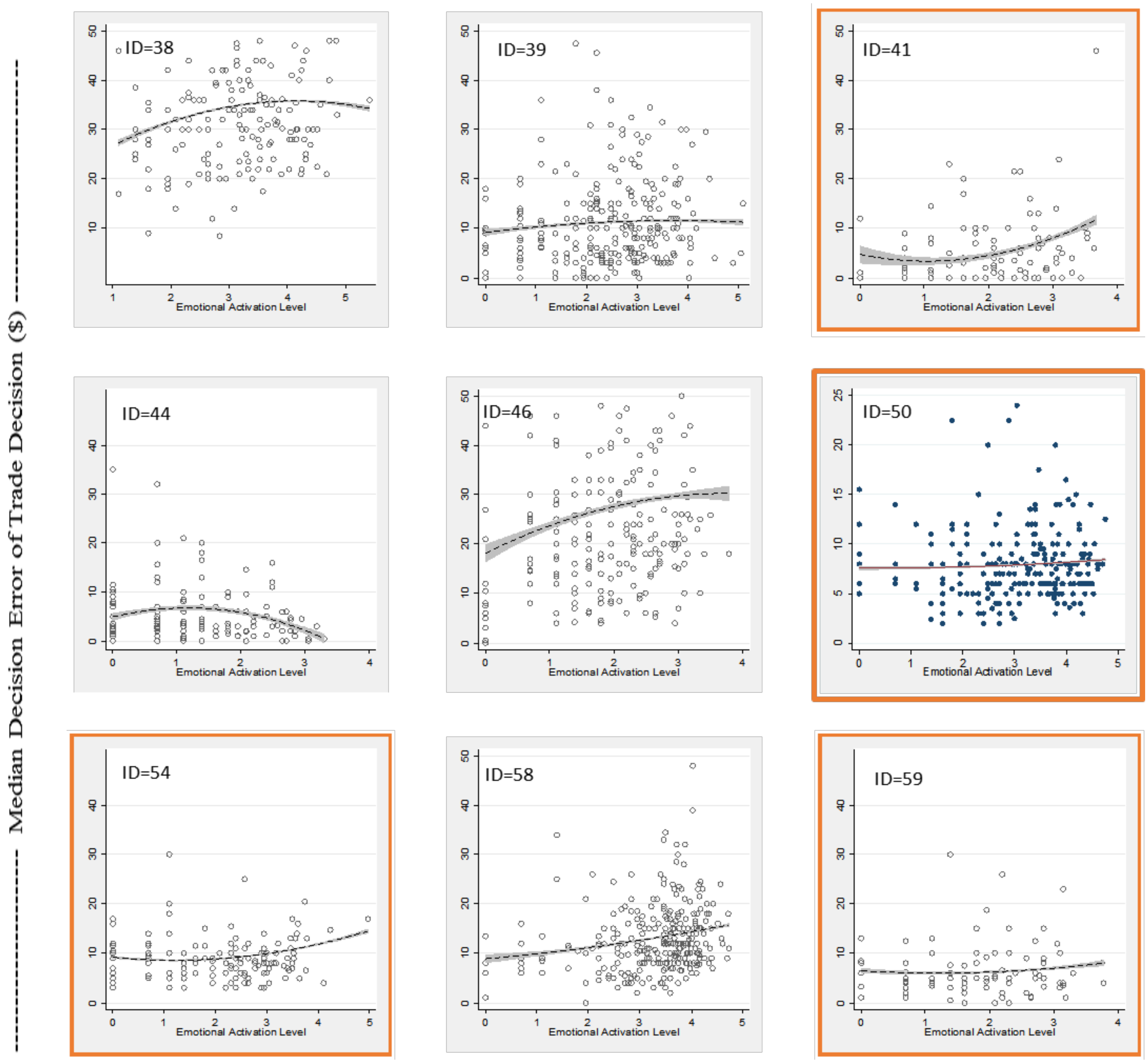

Figure E in S1 File. Scatter Plots of Each Trader's Decision Error and Emotional Activation Relationship

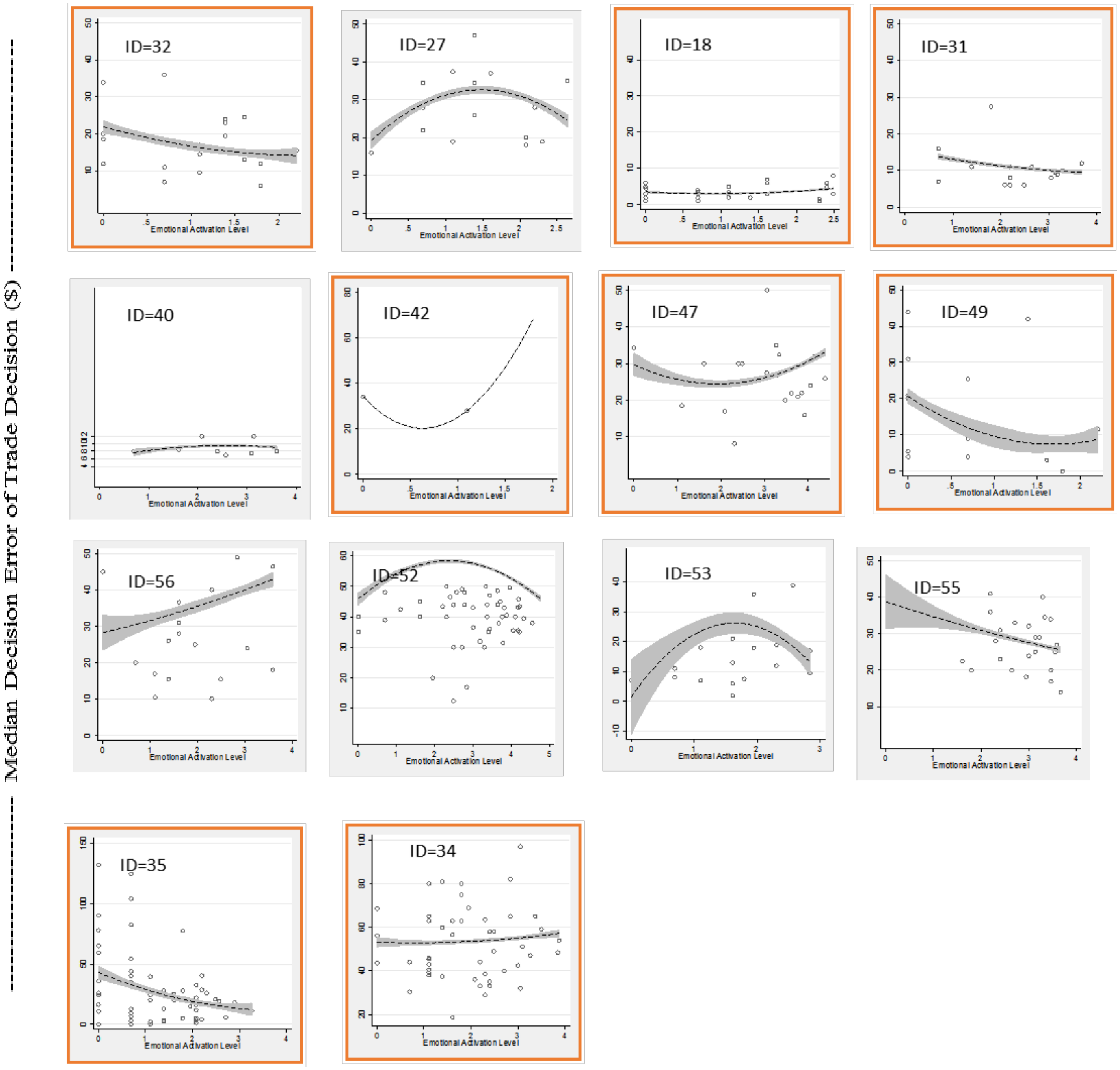

Figure F in S1 File. Scatter Plots of Trader Omitted from the Analysis due to Selection Bias. These 14 traders worked sporadically or too seldomly to include in the analysis (e.g. trader 42) because of the unknown sampling selection bias that can be introduced into the analysis by their atypical participation. Another 12 traders did not have trading data owned by the firm: they were freelance traders. Scatter plots bordered in red indicate that a U-shaped relationship between emotional activation and decision errors was statistically supported; the linear term was significant and negative ($p < .05$) and the quadratic term was significant and positive ($p < .05$). The ID number of each trader is indicated in the top left corner of each plot. Including the 14 additional cases with sketchy data does not produce results dissimilar form those reported for the 30 cases on which there is systematic data. For example, when including all 42 cases in the fixed effects regression reported in the main paper, the coefficients for emotional activation and emotional activation squared are -1.119 ($p=0.016$) and 0.2906 ($p=0.004$) respectively.

Information Complexity & Sentiment Control Variables

We measure not only the emotional activation of IMs but also the information in the messages themselves. This added control is important because information complexity and sentiment can affect a decision's accuracy independent of activation levels [26-28]. Thus, we controlled for the sentiment and information complexity of each trader's IMs and public trading information. Thirteen different sentiment and information complexity variables were added to regressions as control variables.

To verify the validity of these controls as measures of sentiment and information complexity, we correlated the number of trading decisions taken per day with negative vs. positive sentiment and low vs. high information complexity. We defined negative and positive sentiment and low and high information complexity as being below and above the median of these variables, respectively. (Note: emotional activation is a fundamentally different concept then positive or negative emotions or positive or negative sentiments because high emotional activation can occur when emotions or sentiments are negative or positive.) As expected, validation tests confirmed that positive sentiment was positively related to the number of trading decisions and high information complexity was negatively related to the number of trading decisions ($p = 0.0044$ and $p=0.0054$ Wilcoxon rank-sum tests). Other pertinent control variables known to affect the trading behavior and decision acumen of traders were included in our regressions as specified in standard financial models (30-31).

Measures

To calculate the complexity of Bloomberg news feeds (hereafter "Bloombergs") and individual instant messages we used compression, entropy, and perplexity measures. To compute the sentiment of Bloombergs and instant messages we used the Loughran and McDonald method (2011). Traders use Bloomberg newsfeeds to find and summarize the key information out of the surfeit of publicly available data. Bloomberg Corporation systematically tracks an exceptionally broad range of market data and pushes the "filtered" information to traders via e-messages. The ubiquitous use of, and high cost of access to, Bloomberg news feeds in finance attests to their value. The way in which these measures are related to decision making error are described in the text.

Information Complexity Methods

1. *Compression measures*. Five compression algorithms were used (ZIP, TAR, GZIP and 7ZIP). For a string, the complexity using any compression algorithm should be [0,1].
2. *Entropy and Perplexity measures*. For each Bloomberg and instant message, we calculated the entropy and perplexity at the character and word level of analysis. Note that at word level, we only keep words that are "meaningful". Stop words and non-financial words are removed.
3. Formulas:

a. LZMA algorithm: http://en.wikipedia.org/wiki/Lempel-Ziv-Markov_chain_algorithm; GZIP algorithm: http://www.gzip.org/; BZIP2 algorithm: http://www.bzip.org/; DEFLATE algorithm: http://en.wikipedia.org/wiki/DEFLATE; PPMD algorithm: http://en.wikipedia.org/wiki/Prediction_by_partial_matching; Entropy: http://en.wikipedia.org/wiki/Entropy ; Perplexity: http://en.wikipedia.org/wiki/Perplexity

Procedures

For each of the compression algorithms listed above, we calculate information complexity as follows:

1. Use a compression algorithm to compress a string (e.g., abcd abcd") and obtain the "compression ratio", r_1
2. Use the same algorithm to compress the simplest string of the same length, i.e., a string of the same length as the focal string but containing only one character, e.g., "aaaaaaaaa", and obtain the "compression ratio", r_2
3. Complexity is calculated as: $1 - r_1 / (r_2 - 1)$ and ranges from 0 to 1.

Variables

1. *Complexity of IMs and Bloomberg News Feeds* (daily level of information complexity is the average of all values of news in one day): 9 different measures

- blzma: complexity value of applying lzma compression to the news
- bgzip: complexity value of applying gzip compression to the news
- bbzip2: complexity value of applying bzip2 compression to the news
- bdeflate: complexity value of applying deflate compression to the news
- bppmd: complexity value of applying ppmd compression to the news
- bcentropy: character level entropy value of the news
- bcperplexity: character level perplexity value of the news
- bentropy: word level entropy value of the news
- bperplexity: word level perplexity value of the news

Sentiment Methods

Using Loughran and B. McDonald's method (2011), "When is a Liability not a Liability," *Journal of Finance*, V66, pp. 35-65; http://www3.nd.edu/~mcdonald/Word_Lists.html, we computed the sentiment of each Bloomberg and instant message using a finance specific lexicon.

Procedures

Bloomberg Financial words and IM sentiment statistics (each co-variable is obtained by counting the number of news that has at least one financial word (xxx_ct) or counting the number of financial words in all news (xxx_sum) within one day). For example, if there are 3 Bloomberg news items in one day, suppose the first one contains 3 positive financial words while the second one contains 5 positive financial words, and the third one contains no financial words. Then, for this day, the variable *bpos_ct* = 1+1+0 = 2, and *bpos_sum* = 3+2+0 = 5. Bpos_ct and bpos_sum are defined below. *IM Financial words statistics* were calculated using the same method as for Bloombergs.

Variables

- bneg_ct: number of news containing at least one Negative financial word
- bneg_sum: number of Negative financial words in all news
- bpos_ct: number of news containing at least one Positive financial word
- bpos_sum: number of Positive financial words in all news
- bunce_ct: number of news containing at least one Uncertainty financial word
- bunce_sum: number of Uncertainty financial words in all news
- bliti_ct: number of news containing at least one Litigious financial word

- bliti_sum: number of Litigious financial words in all news

Table A in S1 File. Coding Scheme for Defining the Information Complexity and Sentiment of Instant Messages

Complexity of Information Measure									Content of Bloomberg
**lzma**	**gzip**	**bzip2**	**deflate**	**ppmd**	**centropy**	**cperplexity**	**entropy**	**perplexity**	
0.9695	0.8859	0.8224	0.9713	0.9605	5.182	36.3018	4.8402	28.6453	*NCITI DERVS AM: THE CARS - DRIVE...SOUTH? NKY 10D V0L 13.8%?? Inflows net flat for now -buy reits,wsale & sell auto,techs.etc. Though Euro EQs get whacked w/ V2X jumpg to 26.25, US EQ catches late bid & VIX eases 25.5->23.6. CDX.NA.IG still @ hi-end +
0.9694	0.8844	0.823	0.9709	0.9599	5.1926	36.5702	4.5236	23	EURUROMARKETS -0.5% ON WEAK $(EUR1.4313),US RATE CUT LIKELY OCT 31 CASINO Sells property worth E650m(as opposed to Tesco bid rumors CLARINS Q3 sales +8%(10.8% org)to E231.8m above SGe.Reits f'cast EADS ILFC ordered 20 A350s(less than exp),also 74 Drea
0.9692	0.8863	0.8333	0.9714	0.961	5.2794	38.8393	4.2971	19.6585	AUTO EVENTS TODAY: ML Conf Call - transformation at GM & Ford. (Today 10:00am EDT). Please join us to discuss the impact of the pending UAW labour contract. http://research1.ml.com/CFCR/GetDoc.aspx?q=KYQM%2Bs0078%2FltljEpgrEYA%3D%3D ** US markets: Mkts e
0.8106	0.5003	0.361	0.8245	0.7641	0.8722	1.8304	1.585	3	GPS 122/129 UNCH POST EARNINGS
0.8106	0.4907	0.3946	0.8193	0.7571	0.7925	1.7321	1.585	3	CAL pfd 25k at 35 1/2 vs 25.75
0.8106	0.4907	0.4101	0.8193	0.7571	0.7718	1.7074	1.585	3	YRCW 5% 95 1/4 bid 614m vs 17
									**Content of Instant Messages**
0.9676	0.8757	0.813	0.9685	0.9561	4.6372	24.885	4.6439	25	Jason, do you look at MLM, Vulcan, Eagle, Texas Industries, St.Gobain, Lafarge, Cemex, etc? Mike Betts will be in CT next Thursday, March 13, we just had a cancellation for 12pm lunch there, if that would work, let me know.
0.9676	0.8796	0.8203	0.9696	0.9568	4.7569	27.0379	3.9069	15	MACRO: FINANCIALS NOT THE FOCUS TODAY For what feels like the first time in a while, Financials won't be the primary focus today. Microsoft's bid for Yahoo (61% premium) has S&P futures up a quick 15 this morning. I'd still be hesitant to come to
0.9676	0.8768	0.8114	0.9688	0.9568	4.7883	27.6322	4.3219	20	I BET SOUTHEASTERN IS BLOWING OUT THE LVLT TOO -- I WOULDN'T PLA Y ANY $2 STOCK BUT I LOVE TO WATCH. This T is now stupid trading. I'm going to buy some out of attitude. I wish we could take advantage of NYT move but with the short recall pr

0.8871	0.5896	0.2059	0.9016	0.8653	3.8099	14.0252	2.8074	7	i was trying to sell at 68. then went to hit 67.25 bid......toooooo slow.......
0.8871	0.54	0.25	0.894	0.8633	3.9259	15.1986	2	4	mike 1 day i can trade 1000 shares all my stocks move multiple points
0.8871	0.5512	0.257	0.8973	0.8702	4.1443	17.6831	2.585	6	this thing could actually be worth a shot if it opened close to 16.50

Factor Analysis of Information Complexity and Sentiment Measures

Conceptually and empirically, the measures of information complexity should correlate with each other and the measures of sentiment should correlate with each other. If this is the case, it is better to analyze related measures as factors rather than individual measures to avoid multicollinearity problems in the regression analyses.

To this point, we examined all the separate information complexity and sentiment measures for intercorrelations via factor analytic procedures using Stata in an attempt to reduce the 34 separate measures into a more economic set of factors. Factors represent highly correlated individual items, where the high correlation suggests they are measuring a related underlying construct. The protocol is conservative and minimizes noise due to individual differences and multicollinearity in a regression. We used the default factor analysis settings (Principal Axis Factor) and rotated the matrix of loadings to obtain orthogonal (independent) factors (Varimax rotation). We identified items loadings >0.10 only. This produced 4 factors, which are presented below. As expected, measures of information complexity were correlated with each other and measures of sentiment were correlated with each other but information complexity and sentiment were not correlated with each other. In addition, information complexity and sentiment were further parsed along the lines of Bloombergs and instant messages. S2 Table summarizes the factor analysis.

In our regression analyses, we ran separate analyses using the 4-factor variables below and all 34 separate measures of information complexity or sentiment. Using the factors or the separate measures did not affect the relationship between activation and decision error. Factors provided a more consistent pattern across results as expected. The factors consistently showed effects on decision making in the expected directions. The individual measures had a spottier pattern of results when used individually, as expected. Hence, we report the factor variables in our regressions.

Table B in S1 File. Factor Analysis of Information Complexity and Sentiment Measures

Factor	Construct	Individual measures loading together on the same factor
1	Bloomberg Sentiment	(bunce_ct+bunce_sum)/2
2	IM Information Complexity	(ilzma + igzip+ibzip2+ideflate+ippmd+icentropy+icperplexity+ientropy)/8
3	IM Sentiment	(ineg_ct+ineg_sum+ipos_ct+ipos_sum+iunce_ct+iunce_sum+iliti_ct+iliti_sum)/8
4	Bloomberg Information Complexity	(blzma+bgzip+bbzip2+bdeflate+bppmd+bcentropy+bcperplexity)/7

Factor analysis/correlation Number of obs = 770029

Method: principal-component factors Retained factors = 4
Rotation: (unrotated) Number of params = 130

Factor	Eigenvalue	Difference	Proportion	Cumulative	
Factor1	**15.7220**	6.68794	0.4624	0.4624	
Factor2	**9.03414**	5.19822	0.2657	0.7281	
Factor3	**3.83592**	1.61533	0.1128	0.8409	
Factor4	**2.22059**	1.38630	0.0653	0.9063	
Factor5	0.83428	0.12155	0.0245	0.9308	(Note: factors with Eigenvalues <1.0)
Factor6	0.71273	0.28203	0.0210	0.9518	
.					
.					
.					
Factor34	0.00000	0.00000	0.0000	1.0000	

Table C in S1 File. Affective Norms for English Words (Bradley, M.M., & Lang, P.J., 2010)

Description	Word No.	Valence Mean(SD)		Arousal Mean(SD)		Dominance Mean (SD)	
abduction	621	2.76	(2.06)	5.53	(2.43)	3.49	(2.38)
able	1041	6.74	(2)	4.30	(2.17)	6.83	(2.04)
abortion	622	3.50	(2.3)	5.39	(2.8)	4.59	(2.54)
absent	1042	3.69	(1.72)	4.73	(1.76)	4.35	(1.87)
absurd	623	4.26	(1.82)	4.36	(2.2)	4.73	(1.72)
abundance	624	6.59	(2.01)	5.51	(2.63)	5.80	(2.16)
abuse	1	1.80	(1.23)	6.83	(2.7)	3.69	(2.94)
accept	1043	6.80	(2.11)	5.53	(1.96)	5.41	(1.92)
acceptance	625	7.98	(1.42)	5.40	(2.7)	6.64	(1.91)
access	1044	6.14	(1.62)	5.07	(1.68)	6.25	(1.53)
accident	2	2.05	(1.19)	6.26	(2.87)	3.76	(2.22)
accord	1045	5.53	(1.2)	4.24	(1.72)	5.31	(1.2)
accost	1046	4.28	(1.41)	5.24	(1.92)	4.38	(1.61)
accuse	1047	2.54	(1.37)	6.57	(1.93)	4.07	(2.28)
ace	626	6.88	(1.93)	5.50	(2.66)	6.39	(2.31)
ache	627	2.46	(1.52)	5.00	(2.45)	3.54	(1.73)
achievement	3	7.89	(1.38)	5.53	(2.81)	6.56	(2.35)
acre	1048	5.66	(1.26)	4.90	(1.88)	6.04	(1.67)
action	1049	6.63	(1.45)	6.53	(1.87)	5.63	(2.06)
activate	4	5.46	(0.98)	4.86	(2.56)	5.43	(1.84)
actor	1050	7.13	(1.31)	6.07	(1.89)	4.97	(1.73)
ad	1051	5.00	(1.22)	4.29	(1.7)	5.00	(1.12)
adapt	1052	5.72	(1.14)	4.52	(1.86)	5.43	(1.41)
addict	581	2.48	(2.08)	5.66	(2.26)	3.72	(2.54)
addicted	628	2.51	(1.42)	4.81	(2.46)	3.46	(2.23)
adhere	1053	5.03	(1.16)	4.07	(1.74)	4.33	(1.77)
adjust	1054	5.25	(1.55)	5.39	(1.69)	4.86	(1.46)
admire	1055	7.63	(0.93)	6.20	(1.79)	6.27	(1.6)
admired	5	7.74	(1.84)	6.11	(2.36)	7.53	(1.94)
admit	1056	4.93	(1.53)	4.97	(1.86)	4.45	(1.96)
adopt	1057	6.52	(1.29)	4.71	(1.99)	5.88	(1.39)
adorable	6	7.81	(1.24)	5.12	(2.71)	5.74	(2.48)
adore	1058	7.22	(2.01)	5.74	(2.54)	5.19	(1.78)
adorn	1059	6.11	(1.12)	4.56	(1.89)	5.22	(1.28)
adult	546	6.49	(1.5)	4.76	(1.95)	5.75	(2.21)
advantage	629	6.95	(1.85)	4.76	(2.18)	6.36	(2.23)
adventure	630	7.60	(1.5)	6.98	(2.15)	6.46	(1.67)
advice	1060	6.17	(1.42)	5.17	(1.74)	5.47	(2.1)
affair	1061	3.12	(1.94)	6.21	(2.54)	3.71	(2.16)
affection	7	8.39	(0.86)	6.21	(2.75)	6.08	(2.22)
affectionate	1062	7.20	(1.85)	5.67	(2.83)	5.57	(2.21)
affirm	1063	6.17	(1.23)	4.79	(1.37)	6.07	(1.25)
afford	1064	6.27	(2.02)	5.31	(2.11)	5.76	(2.42)
afraid	8	2.00	(1.28)	6.67	(2.54)	3.98	(2.63)
aggravated	1065	2.66	(1.45)	5.79	(2.16)	4.28	(2.02)
aggravation	1066	2.10	(1.06)	6.10	(2.47)	3.83	(1.91)
aggressive	9	5.10	(1.68)	5.83	(2.33)	5.59	(2.4)
agility	22	6.46	(1.57)	4.85	(1.8)	5.87	(1.52)
agony	10	2.43	(2.17)	6.06	(2.67)	4.02	(2.49)
agree	1067	7.00	(1.08)	4.59	(1.56)	6.28	(1.28)
agreement	631	7.08	(1.59)	5.02	(2.24)	6.22	(1.85)
aid	1068	6.93	(1.16)	5.41	(1.78)	5.55	(1.99)
ail	1069	3.70	(1.73)	4.59	(2.01)	4.21	(2.08)
air	632	6.34	(1.56)	4.12	(2.3)	5.10	(1.56)
airplane	1070	6.43	(1.94)	6.77	(1.59)	3.73	(2.24)
airport	1071	5.90	(2.09)	5.97	(2.01)	4.41	(1.82)
alarm	1072	2.86	(1.65)	7.36	(2.3)	3.75	(1.96)
album	1073	6.21	(1.73)	5.18	(2.21)	5.04	(1.6)
alcohol	1074	5.97	(2.15)	5.94	(1.82)	5.50	(1.78)
alcoholic	582	2.84	(2.34)	5.69	(2.36)	4.45	(2.56)
alcove	1075	5.32	(0.98)	4.19	(1.64)	4.93	(1.47)
alert	11	6.20	(1.76)	6.85	(2.53)	5.96	(2.24)
alien	633	5.60	(1.82)	5.45	(2.15)	4.64	(2.07)
alimony	634	3.95	(2)	4.30	(2.29)	4.63	(2.3)
alive	635	7.25	(2.22)	5.50	(2.74)	6.39	(2.15)
allege	1076	4.37	(1.4)	3.87	(1.78)	4.77	(1.04)
allergy	636	3.07	(1.64)	4.64	(2.34)	3.21	(1.77)
alley	637	4.48	(1.97)	4.91	(2.42)	4.00	(1.7)
alone	12	2.41	(1.77)	4.83	(2.66)	3.70	(2.42)
aloof	13	4.90	(1.92)	4.28	(2.1)	4.69	(1.92)

Description	Word No.	Valence Mean(SD)		Arousal Mean(SD)		Dominance Mean (SD)	
alter	1077	5.76	(1.5)	4.93	(2.31)	5.96	(1.29)
amaze	1078	7.57	(1.41)	6.90	(1.94)	5.57	(1.63)
ambition	14	7.04	(1.98)	5.61	(2.92)	6.93	(2.07)
ambitious	1079	7.62	(1.35)	6.57	(2.28)	6.93	(1.92)
ambulance	15	2.47	(1.5)	7.33	(1.96)	3.22	(2.29)
ambush	1080	4.03	(1.8)	6.23	(1.52)	4.00	(2)
amuse	1081	7.57	(1.38)	5.97	(2.28)	5.93	(1.28)
amusement	1082	7.96	(1.22)	6.22	(2.24)	5.74	(1.83)
anarchy	1083	3.58	(1.71)	5.48	(2.17)	3.65	(1.96)
ancestor	1084	5.73	(1.74)	4.27	(2.03)	4.83	(2.05)
angel	16	7.53	(1.58)	4.83	(2.63)	4.97	(2.34)
anger	17	2.34	(1.32)	7.63	(1.91)	5.50	(2.82)
angry	18	2.85	(1.7)	7.17	(2.07)	5.55	(2.74)
anguished	19	2.12	(1.56)	5.33	(2.69)	3.45	(2.37)
animal	1085	6.48	(1.55)	5.55	(1.53)	6.00	(1.6)
ankle	638	5.27	(1.54)	4.16	(2.03)	4.77	(1.74)
annoy	1086	2.96	(1.65)	5.52	(2.49)	4.44	(2)
annoy	20	2.74	(1.81)	6.49	(2.17)	5.09	(2.04)
annoyance	1087	2.97	(1.61)	5.18	(2.44)	4.21	(1.87)
answer	639	6.63	(1.68)	5.41	(2.43)	5.85	(1.88)
antique	1088	5.39	(1.91)	4.06	(2.26)	5.19	(1.99)
anus	1089	4.52	(2.06)	4.28	(2.32)	5.48	(1.36)
anxiety	1090	2.77	(1.59)	6.72	(1.94)	2.72	(1.83)
anxious	21	4.81	(1.98)	6.92	(1.81)	5.33	(1.82)
apartment	1091	6.80	(1.35)	4.75	(1.92)	6.67	(1.88)
apathy	1092	4.30	(1.95)	4.00	(2.1)	4.33	(1.65)
apology	1093	5.00	(2.22)	5.18	(1.66)	5.31	(2.19)
appall	1094	3.61	(2.18)	6.50	(2.67)	4.61	(2.28)
applause	640	7.50	(1.5)	5.80	(2.79)	6.48	(2.11)
apple	1095	6.41	(1.5)	4.17	(2.11)	5.86	(1.64)
appliance	641	5.10	(1.21)	4.05	(2.06)	5.05	(1.34)
application	1096	4.69	(1.65)	5.62	(2.27)	3.90	(1.63)
appreciative	1097	7.43	(1.36)	5.73	(2.23)	5.90	(1.75)
apprehension	1098	3.03	(1.61)	5.68	(2.26)	3.11	(1.4)
arch	1099	5.37	(1.08)	4.37	(2.29)	5.07	(2.02)

Description	Word No.	Valence Mean(SD)		Arousal Mean(SD)		Dominance Mean (SD)	
area	1100	5.45	(1.77)	3.80	(2.19)	5.23	(1.59)
arena	1101	6.40	(1.61)	6.43	(2.05)	5.10	(2.02)
argue	1102	2.83	(1.23)	6.07	(2.12)	4.70	(2.25)
arm	642	5.34	(1.82)	3.59	(2.4)	5.07	(1.5)
army	23	4.72	(1.75)	5.03	(2.03)	5.03	(2.45)
aroma	1103	7.17	(1.81)	5.19	(2.62)	5.50	(1.75)
arouse	1104	7.48	(1.33)	6.97	(2.16)	6.10	(2.14)
aroused	24	7.97	(1)	6.63	(2.7)	6.14	(1.97)
arrogant	25	3.69	(2.4)	5.65	(2.23)	5.14	(2.71)
arrow	1105	5.17	(1.66)	5.37	(1.88)	4.87	(2.01)
arson	1106	2.60	(2.12)	6.58	(2.7)	3.46	(2.48)
art	643	6.68	(2.1)	4.86	(2.88)	5.30	(2.33)
artist	1107	6.59	(1.72)	4.94	(2.44)	5.91	(1.35)
ash	1108	4.04	(1.76)	4.41	(2.06)	4.89	(1.48)
asphalt	1109	5.12	(1.07)	3.77	(2.1)	5.19	(1.72)
aspire	1110	7.21	(1.76)	5.83	(2.25)	7.00	(1.44)
ass	1111	6.09	(2.02)	6.00	(2.17)	5.06	(1.98)
assassin	26	3.09	(2.09)	6.28	(2.53)	4.33	(2.68)
assault	27	2.03	(1.55)	7.51	(2.28)	3.94	(3.1)
assume	1112	4.69	(1.51)	4.97	(1.38)	4.24	(1.88)
assure	1113	7.04	(1.32)	4.52	(2.31)	6.22	(2.19)
astonished	28	6.56	(1.61)	6.58	(2.22)	5.16	(1.79)
astronaut	501	6.66	(1.6)	5.28	(2.11)	5.20	(1.95)
athlete	1114	7.24	(1.74)	6.50	(2.17)	6.67	(1.55)
athletics	644	6.61	(2.08)	6.10	(2.29)	6.12	(2.12)
atlas	1115	5.53	(1.38)	4.53	(1.96)	5.37	(1.25)
atom	1116	4.73	(1.62)	4.00	(1.84)	4.33	(1.69)
attack	1117	2.70	(2.07)	7.03	(2.24)	3.30	(2.52)
attain	1118	6.70	(1.49)	5.53	(2)	6.03	(1.45)
attend	1119	6.30	(1.73)	4.74	(2.1)	5.35	(1.67)
aunt	1120	6.39	(2.04)	4.96	(1.69)	5.39	(1.37)
auto	1121	6.29	(1.67)	4.89	(2.28)	5.19	(1.75)
autumn	29	6.30	(2.14)	4.51	(2.5)	5.15	(1.85)
avalanche	645	3.29	(1.95)	5.54	(2.37)	3.61	(2)
avenge	1122	4.61	(1.26)	5.89	(1.76)	6.33	(1.41)

Description	Word No.	Valence Mean(SD)		Arousal Mean(SD)		Dominance Mean (SD)	
avenue	646	5.50	(1.37)	4.12	(2.01)	5.40	(1.53)
avert	1123	5.17	(1.44)	4.73	(2.23)	4.67	(1.77)
avoid	1124	3.62	(1.45)	5.04	(2.28)	4.26	(1.95)
await	1125	4.64	(1.87)	4.54	(2.78)	3.75	(2.25)
awaken	1126	5.34	(1.88)	5.00	(2.21)	5.09	(1.77)
award	1127	8.44	(1.05)	7.22	(2.03)	7.26	(2.21)
awe	1128	6.48	(1.62)	5.50	(2.05)	4.43	(1.71)
awed	30	6.70	(1.38)	5.74	(2.31)	5.30	(2.03)
awful	1129	2.66	(1.78)	5.72	(2.15)	3.97	(1.99)
awkward	1130	3.20	(1.79)	5.70	(1.82)	3.03	(1.59)
awning	1131	5.10	(1.47)	3.97	(1.87)	5.17	(1.39)
baby	31	8.22	(1.2)	5.53	(2.8)	5.00	(2.8)
backyard	1132	6.87	(1.66)	4.87	(2.5)	5.77	(1.92)
bacon	1133	6.70	(2.46)	5.63	(2.5)	5.81	(2.39)
bad	1134	2.56	(1.36)	5.52	(1.87)	3.84	(1.82)
badge	1135	5.37	(1.71)	4.60	(1.98)	4.90	(1.69)
baffle	1136	4.04	(1.49)	5.48	(1.85)	3.67	(1.55)
bake	647	6.17	(1.71)	5.10	(2.3)	5.49	(1.88)
balcony	1137	5.77	(1.72)	4.60	(2.3)	4.63	(1.65)
bald	1138	3.78	(1.83)	4.26	(2.44)	4.44	(2.04)
ballet	1139	5.61	(2.08)	4.00	(2.08)	5.84	(1.55)
balloons	1140	6.97	(1.61)	4.90	(1.75)	5.53	(1.28)
ban	1141	3.48	(1.53)	5.62	(2.06)	3.90	(2.4)
banana	1142	6.61	(1.93)	4.93	(2.34)	5.61	(1.64)
band	1143	6.55	(1.62)	5.66	(1.91)	5.34	(1.61)
bandage	648	4.54	(1.75)	3.90	(2.07)	4.52	(1.89)
banish	1144	2.46	(1.4)	5.68	(2.84)	3.21	(2.25)
bankrupt	32	2.00	(1.31)	6.21	(2.79)	3.27	(2.39)
banner	649	5.40	(0.83)	3.83	(1.95)	4.80	(1.57)
bar	650	6.42	(2.05)	5.00	(2.83)	5.47	(1.94)
bargain	1145	7.28	(1.3)	5.94	(2)	6.47	(1.22)
bark	1146	4.68	(2.16)	6.07	(2.22)	4.85	(2.14)
barn	1147	5.48	(2.01)	4.27	(2.27)	5.73	(2.2)
barrel	651	5.05	(1.46)	3.36	(2.28)	4.89	(1.57)
basement	1148	4.67	(1.58)	4.73	(2.26)	4.60	(1.94)
basin	1149	4.93	(0.87)	3.83	(1.97)	4.47	(1.2)
basis	1150	4.81	(0.65)	3.83	(1.68)	5.37	(1.33)
basket	547	5.45	(1.15)	3.63	(2.02)	5.76	(1.45)
basketball	1151	6.14	(2.04)	5.94	(2.3)	5.19	(1.94)
bastard	33	3.36	(2.16)	6.07	(2.15)	4.17	(2.4)
batch	1152	6.18	(1.36)	4.63	(1.8)	5.67	(1.41)
bath	502	7.33	(1.45)	4.16	(2.31)	6.41	(1.87)
bathe	1153	7.37	(1.52)	4.63	(2.48)	5.93	(1.96)
bathroom	548	5.55	(1.36)	3.88	(1.72)	5.65	(1.59)
bathtub	652	6.69	(1.57)	4.36	(2.59)	5.76	(1.76)
baton	1154	5.16	(1.53)	4.38	(1.91)	5.22	(1.45)
battleship	1155	4.93	(2.62)	5.85	(2.64)	3.89	(2.21)
bawl	1156	3.41	(1.48)	5.00	(2.06)	3.97	(1.36)
beach	34	8.03	(1.59)	5.53	(3.07)	5.44	(2.52)
beads	1157	5.54	(1.32)	4.11	(2.44)	4.61	(1.77)
bean	1158	5.35	(1.76)	3.97	(2.27)	5.80	(1.99)
bear	1159	4.78	(2.03)	6.52	(2.44)	3.78	(2.24)
beard	1160	5.82	(2.06)	4.30	(2.33)	5.89	(1.95)
beast	653	4.23	(2.41)	5.57	(2.61)	4.89	(2.29)
beautiful	654	7.60	(1.64)	6.17	(2.34)	6.29	(1.81)
beauty	35	7.82	(1.16)	4.95	(2.57)	5.53	(2.1)
bed	549	7.51	(1.38)	3.61	(2.56)	6.88	(1.78)
bedspread	1161	5.60	(1.94)	4.21	(2.18)	5.90	(1.68)
beer	1162	5.96	(2.32)	5.36	(2.44)	5.18	(2.36)
bees	583	3.20	(2.07)	6.51	(2.14)	4.16	(2.11)
beet	1163	4.33	(1.52)	4.03	(2.28)	5.03	(1.32)
beg	1164	2.75	(1.86)	5.00	(2.25)	3.30	(2)
beggar	36	3.22	(2.02)	4.91	(2.45)	4.09	(2.38)
beginning	1165	6.20	(2.09)	4.87	(2.05)	6.10	(2.09)
behave	1166	5.50	(2.05)	3.93	(2.02)	4.56	(2.15)
belief	1167	6.61	(1.79)	5.70	(2.48)	5.85	(2.07)
bell	1168	5.38	(1.4)	4.45	(1.78)	5.10	(1.23)
belly	1169	4.96	(1.71)	4.33	(2.08)	4.96	(1.79)
belong	1170	6.46	(1.71)	5.64	(2.23)	5.75	(1.6)
bench	655	4.61	(1.4)	3.59	(2.07)	4.68	(1.38)

Description	Word No.	Valence Mean(SD)		Arousal Mean(SD)		Dominance Mean (SD)	
benefit	1171	6.59	(1.78)	5.14	(2.23)	6.03	(2.13)
berate	1172	3.53	(2.01)	5.47	(2.37)	3.83	(1.93)
bereavement	656	4.57	(1.7)	4.20	(2.15)	4.33	(1.73)
betray	37	1.68	(1.02)	7.24	(2.06)	4.92	(2.97)
beverage	657	6.83	(1.48)	5.21	(2.46)	5.63	(2.17)
beware	1173	3.52	(1.6)	6.38	(1.93)	3.69	(2.19)
bib	1174	5.57	(1.91)	4.37	(2.09)	4.77	(1.68)
bible	1175	5.97	(2.28)	4.57	(2.75)	4.47	(2.16)
bicker	1176	3.21	(1.37)	5.36	(2.25)	4.61	(1.69)
bicyclist	1177	5.40	(1.61)	4.93	(1.96)	5.13	(1.33)
bikini	1178	6.47	(2.32)	6.27	(2.05)	5.20	(2.19)
binge	1179	3.25	(1.68)	4.94	(2.2)	4.28	(2.52)
binoculars	1180	6.18	(1.54)	4.44	(1.87)	6.19	(1.9)
bird	38	7.27	(1.36)	3.17	(2.23)	4.42	(2.26)
birth	1181	7.64	(1.42)	6.82	(2.36)	4.96	(2.1)
birthday	39	7.84	(1.92)	6.68	(2.11)	5.89	(2.61)
bitch	1182	3.00	(1.98)	6.36	(2.72)	4.36	(2.34)
black	543	5.39	(1.8)	4.61	(2.24)	5.14	(1.79)
blackmail	40	2.95	(1.95)	6.03	(2.7)	3.54	(2.67)
blade	1183	4.30	(2.05)	6.07	(2.08)	4.87	(1.78)
blame	1184	2.77	(1.41)	4.93	(2.02)	3.47	(1.59)
bland	658	4.10	(1.08)	3.29	(1.89)	4.88	(1.27)
blanket	1185	6.94	(1.34)	3.41	(2.15)	5.75	(1.7)
blase	41	4.89	(1.16)	3.94	(1.76)	4.57	(1.44)
blasphemy	659	3.75	(2.26)	4.93	(2.34)	4.75	(1.59)
bleed	1186	2.97	(1.78)	5.64	(2.67)	3.93	(2.14)
bless	42	7.19	(1.69)	4.05	(2.59)	5.52	(2.22)
blight	1187	4.03	(1.65)	4.66	(2.15)	4.40	(1.52)
blind	43	3.05	(1.99)	4.39	(2.36)	3.28	(1.91)
bliss	660	6.95	(2.24)	4.41	(2.95)	6.12	(2.15)
blister	661	2.88	(1.75)	4.10	(2.34)	3.98	(1.9)
blob	1188	5.23	(2.12)	4.81	(2.46)	5.70	(1.86)
blond	662	6.43	(2.04)	5.07	(2.7)	5.74	(1.67)
blonde	1189	6.18	(2.18)	4.86	(2.63)	5.14	(2.37)
blood	1190	3.64	(1.57)	6.00	(2.72)	4.54	(2.44)

Description	Word No.	Valence Mean(SD)		Arousal Mean(SD)		Dominance Mean (SD)	
bloody	584	2.90	(1.98)	6.41	(2)	3.96	(1.89)
bloom	1191	7.21	(1.42)	4.96	(2.03)	5.36	(1.77)
blossom	44	7.26	(1.18)	5.03	(2.65)	5.53	(2.21)
blouse	1192	5.75	(1.71)	4.43	(2.33)	4.71	(2.31)
blowdryer	1193	5.31	(1.33)	3.84	(1.9)	5.41	(1.46)
blubber	663	3.52	(1.99)	4.57	(2.38)	3.86	(1.97)
blue	544	6.76	(1.78)	4.31	(2.2)	5.63	(1.64)
blues	1194	4.11	(2.18)	3.46	(2.01)	4.75	(2.41)
blurt	1195	4.48	(1.09)	5.36	(1.93)	4.75	(1.62)
board	664	4.82	(1.23)	3.36	(2.12)	4.98	(1.77)
boat	1196	7.79	(1.37)	5.31	(2.9)	5.66	(2.16)
body	665	5.55	(2.37)	5.52	(2.63)	5.34	(2.12)
bold	45	6.80	(1.61)	5.60	(2.21)	6.67	(1.81)
bolt	1197	5.42	(1.53)	5.73	(1.99)	5.08	(1.79)
bomb	46	2.10	(1.19)	7.15	(2.4)	4.54	(2.88)
bone	1198	5.00	(1.24)	4.62	(2.62)	4.88	(1.48)
bonus	1199	8.00	(1.8)	6.83	(2.65)	6.83	(2.05)
book	47	5.72	(1.54)	4.17	(2.49)	5.30	(2.05)
boom	1200	5.10	(1.83)	6.67	(1.83)	4.60	(2.25)
boost	1201	6.33	(1.94)	5.52	(2.67)	5.14	(1.68)
booth	1202	5.20	(0.71)	4.20	(1.99)	5.23	(1.59)
boots	1203	5.97	(1.87)	4.67	(2.04)	5.77	(1.76)
bored	48	2.95	(1.35)	2.83	(2.31)	4.11	(1.7)
boring	1204	3.38	(2.54)	2.29	(2.07)	4.18	(1.79)
bothered	1205	3.00	(1.46)	6.17	(2.1)	4.17	(1.93)
bottle	666	6.15	(1.49)	4.79	(2.44)	4.78	(1.65)
bounty	1206	5.08	(1.35)	5.64	(1.85)	4.96	(1.49)
bouquet	667	7.02	(1.84)	5.46	(2.47)	6.15	(1.8)
bowl	49	5.33	(1.33)	3.47	(2.12)	4.69	(1.67)
boxer	585	5.51	(1.8)	5.12	(2.26)	5.10	(1.64)
boy	50	6.32	(1.6)	4.58	(2.37)	5.34	(2.2)
boyfriend	1207	5.74	(2.58)	5.73	(2.5)	4.67	(2.29)
bracelet	1208	6.17	(1.49)	5.10	(1.9)	5.47	(1.11)
braces	1209	3.17	(1.71)	5.24	(1.88)	3.90	(2.23)
brain	1210	6.70	(1.82)	5.53	(2.08)	5.93	(2.08)

Description	Word No.	Valence Mean(SD)		Arousal Mean(SD)		Dominance Mean (SD)	
brave	668	7.15	(1.64)	6.15	(2.45)	7.22	(1.86)
break	1211	4.59	(2.09)	5.23	(2.06)	5.26	(1.73)
breast	51	6.50	(1.78)	5.37	(2.39)	5.39	(2.27)
breath	1212	6.07	(1.46)	4.77	(2.39)	6.00	(1.68)
breathless	1213	4.54	(2.1)	5.85	(1.87)	4.31	(1.76)
breeze	669	6.85	(1.71)	4.37	(2.32)	5.54	(1.67)
bribe	1214	4.14	(1.88)	6.00	(1.62)	4.48	(1.95)
brick	1215	4.79	(1.97)	4.46	(2.13)	5.14	(1.63)
bride	670	7.34	(1.71)	5.55	(2.74)	5.74	(2.36)
bridge	1216	5.26	(1.32)	5.03	(1.82)	4.68	(1.74)
bright	671	7.50	(1.55)	5.40	(2.33)	6.34	(1.82)
broken	672	3.05	(1.92)	5.43	(2.42)	4.14	(1.62)
brood	1217	4.57	(1.98)	4.63	(1.88)	5.21	(1.68)
broom	1218	4.83	(0.79)	3.23	(1.98)	5.23	(1.59)
broth	1219	5.23	(1.65)	3.93	(2)	5.33	(1.56)
brother	52	7.11	(2.17)	4.71	(2.68)	5.12	(2.31)
brow	1220	5.30	(0.79)	3.57	(1.96)	5.13	(1.59)
brownie	1221	7.33	(2.14)	6.10	(2.16)	5.97	(1.97)
bruise	1222	3.38	(1.45)	5.93	(1.62)	4.52	(1.99)
brutal	53	2.80	(1.9)	6.60	(2.36)	4.59	(2.7)
bucket	1223	5.10	(1.29)	4.17	(1.89)	5.34	(1.45)
buffalo	1224	6.04	(1.56)	4.59	(2.47)	4.30	(1.73)
bug	1225	3.73	(2)	5.57	(2.27)	4.50	(1.48)
bugle	1226	5.27	(1.48)	4.73	(1.95)	5.03	(0.85)
building	550	5.29	(1.15)	3.92	(1.94)	5.25	(1.57)
bulb	1227	5.17	(0.91)	4.24	(1.72)	5.10	(1.05)
bulge	1228	3.46	(1.69)	4.82	(2.44)	4.32	(1.76)
bullet	673	3.29	(2.06)	5.33	(2.48)	3.90	(2.61)
bumble	1229	4.83	(1.73)	5.03	(1.92)	4.76	(1.57)
bunny	54	7.24	(1.32)	4.06	(2.61)	4.97	(2.18)
burdened	55	2.50	(1.32)	5.63	(2.07)	5.03	(2.35)
burglar	1230	2.52	(1.45)	6.62	(2.06)	3.41	(2.16)
burial	56	2.05	(1.41)	5.08	(2.4)	3.55	(1.95)
burn	586	2.73	(1.72)	6.22	(1.91)	4.22	(1.83)
bury	1231	3.68	(1.4)	5.68	(1.4)	4.16	(1.32)

Description	Word No.	Valence Mean(SD)		Arousal Mean(SD)		Dominance Mean (SD)	
bus	541	4.51	(1.57)	3.55	(1.8)	4.84	(1.75)
bustle	1232	5.43	(1.57)	5.80	(2.02)	4.93	(1.62)
busy	1233	4.19	(1.6)	4.58	(1.96)	4.69	(1.49)
busybody	674	5.17	(2.02)	4.84	(2.41)	5.45	(1.97)
butler	1234	5.93	(1.82)	4.70	(2.4)	5.48	(2.36)
butt	1235	6.47	(1.61)	5.70	(2.15)	5.97	(1.61)
butter	57	5.33	(1.2)	3.17	(1.84)	4.67	(1.69)
butterfly	58	7.17	(1.2)	3.47	(2.39)	4.65	(2.27)
button	1236	5.21	(1.13)	3.82	(2.34)	5.68	(2.18)
cab	1237	4.53	(1.07)	3.97	(1.88)	4.27	(1.93)
cabinet	675	5.05	(0.31)	3.43	(1.85)	4.73	(1.66)
cake	59	7.26	(1.27)	5.00	(2.37)	5.16	(2.05)
calender	676	5.42	(1.69)	3.84	(2.34)	4.95	(1.41)
calf	1238	5.39	(1.64)	4.11	(2.18)	6.05	(1.45)
calm	1239	6.73	(1.68)	3.60	(2.51)	6.37	(2.06)
camel	1240	5.62	(1.6)	4.34	(1.88)	5.09	(1.35)
campus	1241	6.07	(1.46)	4.79	(1.68)	5.03	(1.52)
cancel	1242	3.46	(1.36)	4.31	(2.04)	4.08	(2)
cancer	60	1.50	(0.85)	6.42	(2.83)	3.42	(2.99)
candlestick	1243	5.43	(1.48)	4.27	(2.21)	5.00	(1.34)
candy	61	6.54	(2.09)	4.58	(2.4)	5.33	(1.91)
cane	677	4.00	(1.8)	4.20	(1.93)	4.27	(1.95)
cannon	678	4.90	(2.2)	4.71	(2.84)	5.17	(2.29)
canoe	1244	6.29	(1.67)	4.79	(2.64)	5.86	(1.56)
canyon	1245	5.76	(1.55)	5.29	(2.48)	3.79	(1.91)
capability	1246	7.00	(1.65)	5.43	(2.32)	6.36	(1.91)
capable	62	7.16	(1.39)	5.08	(2.07)	6.47	(1.94)
cape	1247	6.33	(1.42)	5.03	(1.94)	6.07	(1.28)
car	551	7.73	(1.63)	6.24	(2.04)	6.98	(2.06)
carcass	679	3.34	(1.92)	4.83	(2.07)	4.90	(1.79)
card	1248	5.61	(1.23)	4.43	(2.15)	5.57	(1.64)
care	1249	7.53	(1.36)	6.03	(1.87)	6.37	(1.73)
carefree	63	7.54	(1.38)	4.17	(2.84)	5.78	(2.5)
caress	64	7.84	(1.16)	5.14	(3)	5.83	(2.13)
caring	1250	6.84	(1.92)	4.38	(2.23)	5.78	(1.79)

Description	Word No.	Valence Mean(SD)		Arousal Mean(SD)		Dominance Mean (SD)	
carnival	1251	6.77	(2.01)	6.20	(2.47)	5.60	(1.73)
carpet	1252	5.17	(1.17)	3.66	(1.72)	5.14	(1.09)
carrot	1253	5.43	(1.74)	3.90	(1.84)	5.89	(1.79)
cartoon	1254	6.94	(1.36)	5.52	(2.06)	5.77	(1.8)
carve	1255	5.08	(1.12)	4.72	(1.65)	4.88	(1.3)
carwash	1256	6.00	(1.74)	4.67	(2.11)	5.43	(1.74)
cash	503	8.37	(1)	7.37	(2.21)	6.96	(2.39)
casino	680	6.81	(1.66)	6.51	(2.12)	5.12	(2.15)
cast	1257	4.46	(1.36)	4.50	(1.86)	5.08	(1.55)
castle	1258	7.43	(1.55)	6.13	(2.15)	6.43	(1.99)
castrate	1259	2.16	(1.99)	5.75	(3.05)	2.75	(2.09)
cat	504	5.72	(2.43)	4.38	(2.24)	6.16	(2.05)
cattle	1260	5.50	(1.74)	4.20	(2.14)	5.57	(1.79)
cave	1261	4.78	(1.98)	5.44	(1.85)	4.44	(1.56)
cavern	1262	5.62	(1.63)	5.28	(2.23)	5.28	(1.67)
cavity	1263	2.59	(1.9)	6.14	(2.62)	3.50	(2.22)
cavort	1264	5.10	(1.05)	4.86	(1.94)	4.97	(1.27)
cease	1265	4.48	(1.15)	4.25	(1.71)	4.64	(1.57)
ceiling	1266	5.45	(0.95)	4.04	(2.22)	5.18	(1.47)
cell	587	3.82	(1.7)	4.08	(2.19)	4.12	(2.13)
cellar	681	4.32	(1.68)	4.39	(2.33)	4.66	(1.61)
cemetery	65	2.63	(1.4)	4.82	(2.66)	4.27	(2.14)
cereal	1267	7.35	(1.51)	5.08	(2.61)	6.28	(2.15)
chair	66	5.08	(0.98)	3.15	(1.77)	4.56	(1.6)
chalk	1268	4.89	(1.69)	3.48	(2.24)	4.70	(1.59)
challenge	1269	6.43	(1.57)	6.43	(1.81)	6.07	(2.52)
champ	682	7.18	(1.97)	6.00	(2.43)	6.77	(2)
champagne	1270	7.00	(1.77)	5.93	(2.33)	5.43	(2.06)
champion	67	8.44	(0.9)	5.85	(3.15)	6.50	(2.85)
chance	683	6.02	(1.77)	5.38	(2.58)	4.64	(1.93)
change	1271	5.24	(1.98)	5.25	(2.38)	4.79	(1.99)
chaos	684	4.17	(2.36)	6.67	(2.06)	3.86	(1.95)
character	1272	5.87	(1.68)	5.17	(1.49)	5.77	(1.65)
charm	68	6.77	(1.58)	5.16	(2.25)	5.57	(2.25)
chase	1273	5.07	(2.03)	6.50	(1.95)	4.64	(1.97)

Description	Word No.	Valence Mean(SD)		Arousal Mean(SD)		Dominance Mean (SD)	
checkerboard	1274	5.80	(1.47)	4.21	(1.95)	5.07	(1.94)
cheer	69	8.10	(1.17)	6.12	(2.45)	6.00	(2.06)
cheerful	1275	8.10	(1.35)	6.36	(2.41)	6.79	(1.71)
cheerleader	1276	6.48	(2.13)	5.67	(2.69)	5.81	(1.92)
cheese	1277	6.33	(1.9)	5.20	(2.01)	4.97	(1.85)
cheeseburger	1278	7.61	(1.83)	6.00	(2.26)	6.46	(1.97)
chef	1279	7.26	(1.56)	5.67	(2.56)	6.33	(1.88)
chess	1280	5.48	(1.24)	4.07	(2.1)	5.00	(1.6)
chest	1281	6.26	(1.56)	5.44	(2.39)	5.44	(1.95)
chicken	1282	6.87	(1.66)	5.10	(2.02)	5.83	(1.44)
child	70	7.08	(1.98)	5.55	(2.29)	5.10	(2.3)
chimney	1283	5.72	(1.28)	3.46	(1.84)	5.12	(0.85)
chin	685	5.29	(1.27)	3.31	(1.98)	5.26	(1.48)
chocolate	505	6.88	(1.89)	5.29	(2.55)	5.18	(1.97)
choice	1284	6.94	(2.08)	5.23	(2.54)	7.33	(2.31)
choir	1285	6.41	(1.84)	4.50	(2.41)	5.50	(1.26)
choke	1286	2.38	(1.43)	6.34	(2.6)	2.91	(1.96)
chore	1287	2.74	(1.38)	3.52	(2.47)	3.26	(1.95)
christmas	686	7.80	(1.55)	6.27	(2.56)	5.37	(2.09)
church	71	6.28	(2.31)	4.34	(2.45)	5.00	(2.42)
cider	1288	6.03	(1.78)	4.10	(1.99)	5.48	(1.84)
cigar	1289	4.61	(2.94)	4.14	(2.45)	5.18	(2.31)
cigarette	1290	2.46	(1.75)	5.35	(2.26)	4.54	(2.35)
cinema	1291	7.86	(1.16)	6.18	(2.23)	6.14	(1.76)
circle	687	5.67	(1.26)	3.86	(2.13)	5.03	(1.46)
circus	72	7.30	(1.84)	5.97	(2.59)	5.39	(2.25)
city	73	6.03	(1.37)	5.24	(2.53)	5.74	(2.08)
clan	1292	4.93	(2.2)	5.00	(1.92)	5.04	(2.41)
clarinet	1293	5.70	(1.88)	4.47	(1.93)	5.43	(1.52)
class	1294	4.37	(2.11)	3.93	(2.23)	4.80	(2.17)
clause	1295	4.93	(1.8)	4.20	(1.47)	4.40	(1.28)
claw	1296	4.23	(1.45)	4.81	(1.79)	4.69	(1.12)
clay	1297	5.33	(0.99)	3.83	(1.86)	5.50	(1.36)
clean	1298	7.23	(1.76)	4.80	(2.47)	6.90	(1.84)
cleavage	1299	6.33	(1.99)	5.53	(2.45)	6.03	(1.85)

Description	Word No.	Valence Mean(SD)		Arousal Mean(SD)		Dominance Mean (SD)	
cleft	1300	4.13	(1.72)	3.93	(2.18)	4.67	(1.65)
clench	1301	4.43	(1.38)	5.63	(1.77)	4.37	(1.75)
cliff	553	4.67	(2.08)	6.25	(2.15)	4.35	(2.11)
cliffdiver	1302	5.80	(1.9)	5.93	(2.43)	5.20	(1.95)
climax	1303	6.93	(2.16)	7.18	(2.4)	5.86	(2.21)
cling	1304	4.52	(1.23)	4.23	(1.94)	4.77	(1.65)
clinic	1305	4.72	(1.67)	4.81	(2.02)	4.19	(1.94)
clock	688	5.14	(1.54)	4.02	(2.54)	4.67	(1.97)
closet	1306	5.21	(1.45)	4.57	(1.83)	5.82	(1.68)
clot	1307	3.13	(1.68)	4.70	(2.37)	3.66	(1.93)
cloth	1308	5.27	(1.53)	3.55	(2.05)	5.14	(1.66)
clothe	1309	5.97	(1.71)	4.60	(2.11)	5.80	(1.63)
clothes	1310	6.66	(1.65)	5.86	(2)	6.52	(1.68)
clothing	74	6.54	(1.85)	4.78	(2.88)	5.33	(2.14)
clouds	533	6.18	(2.18)	3.30	(2.08)	5.22	(1.66)
clown	1311	5.39	(2.15)	5.43	(2.23)	4.86	(2.03)
clue	1312	5.57	(1.1)	5.52	(1.43)	5.03	(1.15)
clumsy	689	4.00	(2.22)	5.18	(2.4)	3.86	(1.79)
coach	1313	6.21	(1.66)	5.59	(1.92)	4.83	(2.04)
coarse	690	4.55	(1.42)	4.21	(1.84)	5.00	(1.43)
coast	691	5.98	(1.86)	4.59	(2.31)	5.67	(1.71)
coax	1314	4.76	(1.53)	4.83	(1.73)	5.17	(1.77)
cocaine	1315	3.37	(2.83)	4.93	(3.31)	4.70	(3.24)
cockpit	1316	6.31	(1.65)	5.53	(2.33)	4.78	(2.34)
cockroach	75	2.81	(2.11)	6.11	(2.78)	4.74	(2.58)
cocoa	1317	7.28	(1.22)	5.54	(2.47)	6.04	(1.35)
cod	1318	4.95	(1.66)	4.05	(1.87)	5.13	(1.36)
coddle	1319	4.81	(1.11)	4.43	(1.7)	5.07	(1.66)
coffin	76	2.56	(1.96)	5.03	(2.79)	4.08	(2.54)
cognition	1320	5.57	(1.26)	4.59	(2.1)	5.56	(1.31)
coin	692	6.02	(1.96)	4.29	(2.48)	5.66	(1.68)
cold	693	4.02	(1.99)	5.19	(2.23)	4.69	(1.73)
colony	1321	5.28	(1.56)	4.86	(2.37)	5.57	(1.62)
color	694	7.02	(1.57)	4.73	(2.64)	6.17	(1.82)
column	695	5.17	(0.85)	3.62	(1.91)	4.81	(1.58)

Description	Word No.	Valence Mean(SD)		Arousal Mean(SD)		Dominance Mean (SD)	
comedy	77	8.37	(0.94)	5.85	(2.81)	5.44	(2.08)
comet	1322	6.23	(2.1)	5.97	(2.11)	4.30	(2.38)
comfort	696	7.07	(2.14)	3.93	(2.85)	5.70	(2.05)
comfortable	1323	8.07	(1.14)	3.59	(2.5)	6.70	(2.13)
command	1324	4.57	(1.87)	5.64	(2.53)	4.25	(3.04)
communism	1325	2.80	(2.41)	5.55	(2.71)	3.07	(2.29)
compel	1326	4.97	(1.25)	5.43	(1.89)	5.53	(1.8)
competent	1327	7.07	(1.53)	5.34	(1.93)	6.72	(1.89)
complete	1328	8.17	(1.14)	5.75	(2.17)	7.36	(1.5)
complex	1329	5.25	(1.32)	5.62	(1.7)	5.34	(1.54)
compulsive	1330	4.14	(2.05)	5.70	(2.46)	4.04	(1.93)
computer	552	6.24	(1.61)	4.75	(1.93)	5.29	(1.99)
concentrate	78	5.20	(1.28)	4.65	(2.13)	4.97	(1.75)
concerned	1331	4.06	(1.61)	5.55	(2.13)	4.45	(1.98)
concert	1332	7.66	(1.4)	7.32	(2.06)	5.71	(2.02)
concrete	1333	5.32	(0.98)	4.03	(1.89)	5.77	(1.41)
condom	1334	6.30	(1.66)	6.43	(1.96)	6.30	(2.04)
confident	79	7.98	(1.29)	6.22	(2.41)	7.68	(1.94)
confused	80	3.21	(1.51)	6.03	(1.88)	4.24	(1.91)
confusion	1335	3.46	(1.93)	6.07	(2.34)	3.04	(1.91)
conquer	1336	6.86	(1.72)	6.79	(1.79)	7.25	(1.94)
conquest	1337	5.85	(1.71)	6.50	(1.9)	5.73	(2.31)
consoled	81	5.78	(1.64)	4.53	(2.22)	4.44	(1.84)
constructive	1338	6.13	(1.43)	5.13	(1.78)	5.83	(1.78)
contempt	82	3.85	(2.13)	5.28	(2.04)	5.13	(1.73)
contents	83	4.89	(0.89)	4.32	(2.14)	4.85	(1.49)
context	84	5.20	(1.38)	4.22	(2.24)	5.17	(1.39)
continuation	1339	5.70	(1.58)	4.93	(2.24)	5.13	(1.59)
control	1340	6.46	(1.35)	5.93	(2.12)	6.79	(2.44)
controlling	85	3.80	(2.25)	6.10	(2.19)	5.17	(3.15)
convey	1341	5.61	(1.37)	4.64	(2.04)	5.75	(1.67)
cook	697	6.16	(1.89)	4.44	(1.96)	5.14	(1.49)
cookie	1342	7.60	(1.43)	5.43	(2.28)	6.17	(1.78)
cookout	1343	7.31	(1.69)	6.14	(2.29)	5.97	(1.61)
cool	1344	6.83	(1.34)	4.17	(2.27)	5.97	(1.38)

Description	Word No.	Valence Mean(SD)		Arousal Mean(SD)		Dominance Mean (SD)	
cope	1345	4.60	(1.94)	4.66	(2.14)	4.76	(2.21)
copper	1346	5.35	(0.95)	3.61	(2.03)	5.29	(1.42)
cord	698	5.10	(1.09)	3.54	(2.09)	5.00	(1.22)
core	1347	4.80	(1.19)	4.18	(1.76)	5.50	(1.45)
cork	699	5.22	(1.13)	3.80	(2.18)	4.98	(1.04)
corn	1348	6.00	(1.69)	4.57	(1.93)	5.71	(1.86)
corner	700	4.36	(1.21)	3.91	(1.92)	4.12	(1.66)
corpse	86	2.18	(1.48)	4.74	(2.94)	3.59	(2.44)
corridor	701	4.88	(1.14)	3.63	(2.41)	5.00	(1.48)
corrupt	702	3.32	(2.32)	4.67	(2.35)	4.64	(2.3)
costume	1349	7.34	(1.26)	6.00	(1.91)	6.86	(1.56)
cottage	87	6.45	(1.52)	3.39	(2.54)	5.39	(1.78)
couch	1350	6.78	(1.64)	4.03	(2.41)	5.83	(2.13)
counselor	1351	4.68	(1.94)	4.54	(2.03)	4.07	(1.88)
country	1352	5.93	(1.91)	5.26	(1.93)	5.96	(1.93)
couple	506	7.41	(1.97)	6.39	(2.31)	6.02	(2.28)
coupon	1353	6.43	(1.57)	5.10	(2.29)	5.83	(2.07)
court	1354	3.44	(2.41)	5.67	(2.81)	3.56	(2.79)
courtyard	1355	5.75	(1.35)	4.00	(2.27)	5.30	(1.73)
cousin	1356	6.32	(2.44)	4.87	(2.33)	5.13	(1.81)
cow	554	5.57	(1.53)	3.49	(2.13)	5.32	(1.61)
coward	703	2.74	(1.64)	4.07	(2.19)	2.83	(1.61)
cowboy	1357	6.48	(1.88)	5.75	(2.07)	5.71	(1.74)
coyote	1358	4.86	(2.19)	5.19	(2.68)	3.93	(2.04)
cozy	88	7.39	(1.53)	3.32	(2.28)	4.89	(2.28)
crab	1359	5.28	(1.87)	4.72	(2.28)	5.45	(1.7)
crackers	1360	5.80	(1.4)	4.47	(1.96)	5.50	(1.28)
craft	1361	6.54	(1.67)	4.70	(2.27)	5.33	(2.02)
cram	1362	3.16	(2)	6.40	(2.82)	3.97	(2.59)
cranky	1363	2.90	(1.9)	5.36	(2.36)	4.39	(1.71)
crash	89	2.31	(1.44)	6.95	(2.44)	3.44	(2.21)
crave	1364	4.88	(1.58)	6.13	(1.43)	3.97	(1.78)
crazy	1365	5.93	(1.95)	6.28	(2.25)	5.13	(2.33)
create	1366	6.79	(1.95)	5.75	(1.6)	5.89	(2.2)
creek	1367	6.55	(1.68)	4.62	(2.44)	5.17	(1.61)

Description	Word No.	Valence Mean(SD)		Arousal Mean(SD)		Dominance Mean (SD)	
crime	704	2.89	(2.06)	5.41	(2.69)	4.12	(2.24)
criminal	705	2.93	(1.66)	4.79	(2.51)	3.34	(1.73)
cripple	1368	2.89	(1.66)	5.14	(2.14)	3.64	(1.62)
crisis	706	2.74	(2.23)	5.44	(3.07)	3.60	(2.47)
critic	1369	3.04	(1.48)	5.67	(2.27)	3.26	(2.3)
criticism	1370	2.75	(1.76)	6.18	(2.4)	3.11	(1.89)
critique	1371	4.39	(1.87)	5.37	(2.06)	4.07	(2.12)
crocodile	1372	5.00	(2.36)	6.03	(2.22)	4.17	(2)
crossword	1373	5.44	(1.58)	3.96	(1.58)	5.37	(1.18)
crowd	1374	4.93	(1.6)	5.30	(2.05)	3.87	(1.98)
crown	90	6.58	(1.42)	4.28	(2.53)	6.06	(2.15)
crucify	91	2.23	(1.72)	6.47	(2.47)	3.74	(2.48)
crude	707	3.12	(1.65)	5.07	(2.37)	4.27	(1.94)
cruel	92	1.97	(1.67)	5.68	(2.65)	4.24	(2.84)
cruise	1375	7.17	(1.68)	5.83	(2.69)	5.63	(1.65)
cruiseship	1376	7.66	(1.45)	6.76	(2.29)	5.31	(2.14)
crumbs	1377	4.27	(1.71)	4.50	(2.06)	5.31	(1.12)
crunch	1378	5.83	(1.34)	5.37	(1.94)	5.57	(1.68)
crush	1379	5.90	(2.07)	6.70	(1.82)	4.47	(2.18)
crushed	93	2.21	(1.74)	5.52	(2.87)	3.36	(2.69)
crutch	708	3.43	(1.62)	4.14	(2.05)	3.91	(1.79)
cuddle	94	7.72	(1.92)	4.40	(2.67)	5.85	(2.42)
cuisine	709	6.64	(1.48)	4.39	(1.99)	5.41	(1.19)
cult	1380	2.48	(1.85)	6.15	(2.71)	3.44	(2.68)
culture	1381	5.90	(2.34)	4.90	(2.63)	5.57	(2.69)
cup	1382	5.44	(1.05)	3.70	(2.16)	5.63	(1.9)
cupboard	1383	4.79	(1.21)	3.83	(2.16)	4.90	(1.45)
cupcake	1384	7.20	(1.85)	5.93	(2.38)	5.67	(2.01)
cure	1385	8.18	(1.25)	6.57	(2.23)	6.04	(2.28)
curious	95	6.08	(1.63)	5.82	(1.64)	5.42	(1.6)
curtains	710	4.83	(0.83)	3.67	(1.83)	5.05	(1.56)
curve	1386	5.54	(1.2)	5.07	(1.69)	5.19	(1.3)
custom	96	5.85	(1.53)	4.66	(2.12)	5.00	(1.87)
cut	711	3.64	(2.08)	5.00	(2.32)	4.70	(1.98)
cute	97	7.62	(1.01)	5.53	(2.71)	4.86	(2.32)

Description	Word No.	Valence Mean(SD)		Arousal Mean(SD)		Dominance Mean (SD)	
cyclone	98	3.60	(2.38)	6.36	(2.89)	4.89	(2.56)
dagger	99	3.38	(1.77)	6.14	(2.64)	4.52	(2.27)
daisy	1387	7.48	(1.53)	3.96	(2.61)	5.33	(2.5)
damage	712	3.05	(1.65)	5.57	(2.26)	3.88	(1.86)
damn	1388	4.52	(2.23)	5.43	(2.38)	5.18	(2.11)
damp	1389	3.68	(1.56)	4.07	(1.73)	4.44	(1.55)
dance	1390	7.38	(1.84)	6.71	(1.98)	6.04	(1.77)
dancer	507	7.14	(1.56)	6.00	(2.2)	6.02	(1.93)
danger	713	2.95	(2.22)	7.32	(2.07)	3.59	(2.31)
dare	1391	5.76	(2.2)	6.57	(2.46)	5.00	(2.72)
dark	714	4.71	(2.36)	4.28	(2.21)	4.84	(2.15)
daughter	1392	6.36	(1.99)	5.11	(1.99)	5.68	(1.93)
dawn	715	6.16	(2.33)	4.39	(2.81)	5.16	(2.23)
day	1393	6.66	(1.43)	5.00	(2.02)	5.88	(1.43)
daylight	716	6.80	(2.17)	4.77	(2.5)	5.48	(2.14)
daze	1394	5.04	(1.55)	4.00	(2.17)	4.59	(1.47)
dazzle	717	7.29	(1.09)	6.33	(2.02)	5.62	(1.81)
dead	588	1.94	(1.76)	5.73	(2.73)	2.84	(2.32)
deadly	1395	2.73	(1.89)	6.62	(2.25)	3.46	(2.14)
death	100	1.61	(1.4)	4.59	(3.07)	3.47	(2.5)
debt	101	2.22	(1.17)	5.68	(2.74)	3.02	(2.16)
decapitate	1396	2.45	(1.9)	6.41	(2.51)	3.28	(2.45)
decay	1397	2.68	(1.66)	4.44	(2.28)	3.63	(1.84)
deceit	718	2.90	(1.63)	5.68	(2.46)	3.95	(2.12)
decency	1398	5.83	(1.78)	4.50	(1.63)	5.70	(1.37)
decompose	102	3.20	(1.81)	4.65	(2.39)	4.02	(1.91)
decorate	719	6.93	(1.3)	5.14	(2.39)	6.05	(1.86)
deed	1399	5.85	(1.46)	5.15	(2.09)	5.73	(1.76)
defeat	1400	2.97	(2.34)	5.63	(2.43)	3.60	(2.34)
defeated	103	2.34	(1.66)	5.09	(3)	3.11	(2.34)
defecate	1401	3.33	(2.45)	5.14	(2.85)	4.77	(2.16)
defend	1402	6.07	(1.89)	5.97	(2.4)	6.27	(1.98)
defer	1403	4.10	(1.54)	4.48	(2.37)	4.76	(2.44)
defiant	104	4.26	(2.12)	6.10	(2.51)	5.77	(2.4)
deformed	720	2.41	(1.66)	4.07	(2.34)	3.95	(2.18)

Description	Word No.	Valence Mean(SD)		Arousal Mean(SD)		Dominance Mean (SD)	
defy	1404	5.40	(2.11)	5.63	(2.28)	6.00	(2.27)
delayed	721	3.07	(1.74)	5.62	(2.39)	3.64	(1.94)
delight	1405	7.48	(1.84)	6.68	(2.2)	5.68	(2.02)
delight	105	8.26	(1.04)	5.44	(2.88)	5.79	(2.24)
delude	1406	4.71	(1.04)	4.10	(1.79)	5.20	(1.16)
demand	1407	3.76	(1.57)	5.75	(1.71)	4.86	(2.53)
democracy	1408	6.55	(2.03)	5.39	(2.36)	6.04	(2.03)
demon	106	2.11	(1.56)	6.76	(2.68)	4.89	(2.89)
den	1409	5.72	(1.49)	3.50	(2)	6.17	(1.76)
denial	1410	3.28	(1.44)	6.03	(1.68)	4.06	(2.14)
denote	1411	4.63	(1.27)	4.40	(1.87)	4.83	(1.46)
dent	1412	2.93	(1.49)	5.69	(2.02)	4.03	(1.86)
dentist	589	4.02	(2.23)	5.73	(2.13)	3.80	(2.16)
deny	1413	3.79	(1.29)	4.86	(1.76)	4.36	(1.99)
depart	1414	3.63	(2.39)	4.37	(2.48)	3.63	(2.2)
depressed	107	1.83	(1.42)	4.72	(2.95)	2.74	(2.13)
depression	108	1.85	(1.67)	4.54	(3.19)	2.91	(2.27)
derelict	722	4.28	(1.84)	4.10	(1.94)	4.78	(1.56)
descent	1415	5.00	(1.47)	4.58	(2.06)	4.83	(1.83)
desert	1416	4.96	(2.5)	4.82	(2.34)	5.00	(2.09)
deserter	109	2.45	(1.8)	5.50	(2.55)	3.77	(2.29)
desire	508	7.69	(1.39)	7.35	(1.76)	6.49	(1.83)
desk	1417	4.66	(1.14)	4.03	(1.72)	5.55	(1.94)
despairing	110	2.43	(1.47)	5.68	(2.37)	3.43	(2.11)
despise	111	2.03	(1.38)	6.28	(2.43)	4.72	(2.8)
destroy	112	2.64	(2.03)	6.83	(2.38)	4.94	(2.86)
destruction	723	3.16	(2.44)	5.82	(2.71)	3.93	(2.29)
detach	1418	3.19	(1.57)	4.19	(2.32)	3.93	(2.34)
detached	113	3.86	(1.88)	4.26	(2.57)	3.63	(2.15)
detail	724	5.55	(1.58)	4.10	(2.24)	5.21	(1.6)
detain	1419	3.03	(1.35)	5.52	(2.13)	3.58	(1.91)
detest	114	2.17	(1.3)	6.06	(2.39)	5.83	(2.6)
devil	115	2.21	(1.99)	6.07	(2.61)	5.35	(2.75)
devote	1420	6.32	(1.49)	5.37	(1.88)	6.19	(1.82)
devoted	116	7.41	(1.37)	5.23	(2.21)	6.18	(2.36)

Description	Word No.	Valence Mean(SD)		Arousal Mean(SD)		Dominance Mean (SD)	
devour	1421	5.62	(2.13)	5.39	(2.02)	5.00	(2.09)
dew	1422	5.59	(1.52)	3.64	(1.79)	5.10	(1.74)
diamond	117	7.92	(1.2)	5.53	(2.96)	5.54	(2.28)
diary	1423	5.73	(1.04)	3.69	(1.95)	6.38	(2.02)
dice	1424	5.72	(1.28)	4.96	(2.15)	4.64	(1.93)
die	1425	2.48	(1.7)	6.38	(2.64)	2.55	(2.23)
difficult	1426	2.93	(1.59)	5.96	(2.1)	4.19	(2.59)
dignified	118	7.10	(1.26)	4.12	(2.29)	6.12	(2.4)
dinner	509	7.16	(1.5)	5.43	(2.14)	6.10	(1.87)
diploma	119	8.00	(1.39)	5.67	(2.8)	6.76	(2.5)
dirt	725	4.17	(1.77)	3.76	(2.26)	4.83	(1.82)
dirty	590	3.08	(2.05)	4.88	(2.29)	4.70	(2.12)
disability	1427	2.52	(1.27)	4.46	(2.49)	2.43	(1.5)
disappoint	120	2.39	(1.44)	4.92	(2.64)	3.29	(2.32)
disappointed	1428	2.21	(1.47)	4.82	(2.88)	3.11	(2.13)
disappointment	1429	2.37	(1.27)	4.60	(2.4)	3.20	(2.04)
disaster	121	1.73	(1.13)	6.33	(2.7)	3.52	(2.42)
discomfort	726	2.19	(1.23)	4.17	(2.44)	3.86	(2.26)
discount	1430	7.48	(1.48)	6.10	(2.02)	5.94	(1.91)
discouraged	122	3.00	(2.16)	4.53	(2.11)	3.61	(2.01)
disdainful	123	3.68	(1.9)	5.04	(2.14)	4.55	(1.92)
disease	1431	2.03	(1.24)	5.32	(2.47)	2.71	(1.84)
disgusted	124	2.45	(1.41)	5.42	(2.59)	4.34	(1.94)
disgusting	1432	2.96	(1.55)	5.18	(2.28)	3.64	(2.04)
dishes	1433	3.62	(1.56)	4.06	(2.23)	4.84	(1.76)
disk	1434	5.25	(0.89)	3.86	(1.82)	5.64	(1.45)
disloyal	125	1.93	(1.61)	6.56	(2.21)	3.79	(2.75)
disorder	1435	2.72	(1.46)	5.42	(2.32)	2.71	(1.55)
displeased	126	2.79	(2.23)	5.64	(2.48)	4.19	(2.19)
distracted	1436	4.48	(1.95)	4.56	(2.26)	4.07	(1.82)
distress	1437	2.67	(2.01)	5.70	(2.34)	3.03	(1.94)
distressed	127	1.94	(1.1)	6.40	(2.38)	3.76	(2.41)
disturb	727	3.66	(2)	5.80	(2.39)	4.55	(1.9)
diver	510	6.45	(1.55)	5.04	(2.1)	5.04	(1.91)
divert	1438	4.44	(1.72)	5.22	(2.44)	4.89	(2.26)
divorce	128	2.22	(1.88)	6.33	(2.71)	3.26	(2.24)
doctor	129	5.20	(2.54)	5.86	(2.7)	4.89	(2.75)
dog	511	7.57	(1.66)	5.76	(2.5)	6.25	(2.1)
doll	728	6.09	(1.96)	4.24	(2.43)	4.61	(2.07)
dollar	729	7.47	(1.72)	6.07	(2.67)	6.33	(2.42)
donate	1439	6.89	(1.69)	4.48	(2.15)	6.70	(1.79)
donkey	1440	5.54	(1.5)	4.22	(1.97)	5.67	(1.52)
donor	1441	6.43	(1.71)	5.11	(1.97)	6.07	(1.86)
door	130	5.13	(1.44)	3.80	(2.29)	4.69	(1.72)
doubt	1442	3.43	(1.97)	4.50	(2.15)	3.64	(1.93)
doubtful	1443	3.41	(1.18)	4.28	(1.83)	3.76	(1.72)
dough	1444	6.09	(1.59)	4.59	(1.93)	5.69	(1.79)
dove	730	6.90	(1.54)	3.79	(2.28)	5.48	(1.7)
dragon	1445	5.43	(1.85)	5.50	(2.33)	4.39	(1.93)
drama	1446	3.86	(2.59)	6.39	(1.93)	5.32	(2.36)
dreadful	131	2.26	(1.91)	5.84	(2.62)	4.10	(2.36)
dream	132	6.73	(1.75)	4.53	(2.72)	5.53	(1.98)
dreams	1447	7.14	(1.46)	6.48	(1.88)	5.38	(2.38)
dreary	731	3.05	(1.58)	2.98	(2.18)	3.81	(1.64)
dress	133	6.41	(1.34)	4.05	(1.89)	5.00	(1.89)
drill	1448	4.40	(1.57)	5.40	(1.69)	4.80	(1.73)
driver	1449	6.13	(1.28)	4.87	(1.8)	6.35	(1.87)
droop	1450	3.21	(1.74)	3.39	(1.79)	3.96	(0.96)
drown	1451	2.20	(1.68)	6.84	(2.17)	2.68	(2.32)
drown	591	1.92	(1.48)	6.57	(2.33)	2.86	(1.99)
drugs	1452	3.76	(2.2)	6.00	(2.39)	4.75	(2.62)
drunk	1453	6.17	(2.5)	6.11	(2.46)	4.54	(2.59)
dryer	1454	5.03	(1.12)	3.79	(1.76)	5.50	(1.5)
ducks	1455	6.20	(1.56)	4.34	(2.18)	5.24	(1.43)
duct	1456	4.86	(1.06)	4.17	(1.81)	5.17	(1.67)
duet	1457	6.27	(1.26)	5.43	(1.94)	5.27	(1.51)
dumb	1458	3.00	(2.15)	4.24	(2.21)	3.69	(1.91)
dummy	732	3.38	(1.7)	4.35	(2.25)	3.67	(2.02)
dump	733	3.21	(1.87)	4.12	(2.36)	3.83	(1.87)
dune	1459	5.03	(1.57)	4.17	(2.02)	5.10	(1.4)

Description	Word No.	Valence Mean(SD)		Arousal Mean(SD)		Dominance Mean (SD)	
dusk	1460	5.40	(1.5)	4.37	(1.87)	4.77	(1.41)
dust	1461	3.90	(1.89)	4.10	(1.99)	4.50	(1.57)
dustpan	555	3.98	(1.68)	3.43	(2)	5.45	(1.81)
duty	1462	5.81	(1.83)	5.06	(2.08)	5.19	(1.82)
dwarf	1463	5.11	(1.85)	4.32	(1.74)	5.14	(1.82)
dwell	1464	4.87	(1.65)	4.00	(2.02)	5.35	(1.89)
eager	1465	7.32	(1.28)	6.57	(2.04)	5.14	(1.78)
eagerness	1466	6.21	(1.89)	6.29	(1.84)	5.39	(1.5)
eagle	1467	6.03	(1.66)	4.76	(1.86)	4.79	(1.76)
earth	134	7.15	(1.67)	4.24	(2.49)	5.61	(2.3)
ease	1468	6.93	(1.46)	3.46	(2.41)	6.71	(2.02)
easel	1469	5.41	(1.43)	4.43	(1.71)	5.43	(1.97)
easy	734	7.10	(1.91)	4.48	(2.82)	7.00	(1.63)
easygoing	135	7.20	(1.5)	4.30	(2.52)	5.25	(1.75)
eat	136	7.47	(1.73)	5.69	(2.51)	5.60	(2.12)
echo	1470	6.17	(1.62)	5.07	(1.74)	5.17	(1.39)
ecstasy	735	7.98	(1.52)	7.38	(1.92)	6.68	(2.08)
ecstatic	1471	8.41	(1.27)	7.71	(1.82)	6.14	(2.03)
education	137	6.69	(1.77)	5.74	(2.46)	6.15	(2.35)
effective	1472	7.06	(1.69)	5.03	(2.44)	6.13	(2.5)
efficient	1473	7.07	(1.63)	5.57	(2.15)	6.43	(2.36)
egg	736	5.29	(1.82)	3.76	(2.39)	4.49	(2.16)
ego	1474	5.07	(1.31)	4.66	(1.45)	6.66	(1.86)
ejaculate	1475	5.92	(1.44)	6.62	(1.58)	5.65	(2.21)
elated	138	7.45	(1.77)	6.21	(2.3)	5.53	(2.35)
elbow	737	5.12	(0.92)	3.81	(2.14)	4.88	(1.52)
elderly	1476	4.86	(2.05)	4.03	(2.41)	3.83	(1.85)
elegant	139	7.43	(1.26)	4.53	(2.65)	5.95	(2.09)
elephant	1477	6.48	(1.72)	5.59	(2.45)	4.70	(2.46)
elevator	738	5.44	(1.18)	4.16	(1.99)	4.32	(1.69)
embark	1478	6.47	(1.57)	5.83	(2.45)	6.17	(1.66)
embarrass	1479	2.50	(1.45)	6.14	(2.4)	2.78	(2.36)
embarrassed	140	3.03	(1.85)	5.87	(2.55)	2.87	(1.99)
embarrassment	1480	2.34	(1.82)	6.54	(2.28)	2.71	(2.14)
embattled	141	4.39	(1.63)	5.36	(2.37)	4.81	(1.79)

Description	Word No.	Valence Mean(SD)		Arousal Mean(SD)		Dominance Mean (SD)	
emotional	1481	4.36	(1.91)	5.75	(2.37)	4.29	(1.63)
empathy	1482	5.32	(2.42)	4.81	(2.2)	4.33	(2.09)
empire	1483	6.26	(1.67)	5.65	(1.84)	6.29	(2.55)
employment	147	6.47	(1.81)	5.28	(2.13)	5.73	(2.08)
enable	1484	5.93	(1.44)	5.10	(1.61)	5.90	(1.78)
encourage	1485	7.27	(1.53)	5.55	(1.99)	6.48	(1.35)
end	1486	3.44	(1.64)	4.44	(2.17)	4.41	(1.78)
endure	1487	5.50	(1.81)	5.56	(1.92)	5.91	(2.05)
energy	1488	7.23	(1.55)	6.90	(1.94)	6.93	(1.62)
enforcement	1489	4.17	(1.49)	5.73	(1.55)	4.27	(2.29)
engage	1490	6.31	(1.81)	5.96	(2.11)	5.23	(1.88)
engaged	143	8.00	(1.38)	6.77	(2.07)	6.49	(2.22)
engine	148	5.20	(1.18)	3.98	(2.34)	5.00	(1.78)
engulf	1491	4.74	(1.7)	4.93	(2.32)	4.37	(2.54)
enjoy	1492	8.17	(1.29)	6.33	(1.94)	6.40	(1.22)
enjoyment	145	7.80	(1.2)	5.20	(2.72)	6.46	(1.77)
enlist	1493	4.03	(1.93)	5.47	(1.88)	4.41	(2)
ennui	146	5.09	(1.76)	4.40	(2.33)	4.67	(1.8)
enraged	149	2.46	(1.65)	7.97	(2.17)	6.33	(2.92)
enthusiastic	1494	8.17	(1.23)	7.38	(2.24)	6.75	(1.71)
entry	1495	5.69	(1.12)	4.72	(1.94)	5.81	(1.47)
envious	1496	3.57	(1.76)	5.57	(1.85)	4.54	(2.03)
envy	1497	3.41	(1.54)	5.50	(1.74)	4.12	(1.91)
enzyme	1498	4.90	(1.63)	4.34	(2.3)	4.17	(1.26)
erase	1499	4.17	(1.82)	4.10	(2.09)	4.87	(1.8)
erasure	1500	4.84	(0.68)	4.44	(1.7)	5.12	(1.21)
erect	1501	6.26	(2.29)	6.40	(2.34)	5.87	(1.96)
erode	1502	3.67	(1.65)	5.17	(1.91)	3.97	(1.7)
erotic	512	7.43	(1.53)	7.24	(1.97)	6.39	(2.16)
err	1503	4.16	(2.02)	4.88	(1.76)	4.31	(1.55)
errand	150	4.58	(1.74)	3.85	(1.92)	4.78	(1.51)
erupt	1504	5.43	(1.57)	6.57	(2.14)	3.80	(1.97)
essence	1505	6.75	(1.62)	5.18	(2.25)	5.61	(1.52)
estate	1506	6.59	(1.96)	4.86	(2.33)	5.66	(2)
evade	1507	4.47	(1.83)	5.60	(2.24)	5.53	(2.27)

Description	Word No.	Valence Mean(SD)		Arousal Mean(SD)		Dominance Mean (SD)	
event	740	6.21	(1.63)	5.10	(2.4)	5.52	(1.57)
evict	1508	3.04	(1.4)	5.68	(2.12)	2.92	(1.91)
evil	741	3.23	(2.64)	6.39	(2.44)	5.25	(2.6)
exalt	1509	6.87	(1.59)	6.00	(2.42)	5.76	(1.66)
exam	1510	2.76	(1.99)	7.03	(2.43)	3.34	(2.18)
exasperation	1511	4.18	(2)	5.64	(2.59)	4.57	(2.03)
excel	1512	7.45	(1.55)	5.45	(2.16)	7.00	(1.75)
excellence	151	8.38	(0.96)	5.54	(2.67)	7.28	(2.32)
excite	1513	7.60	(1.29)	7.16	(1.49)	6.16	(1.43)
excitement	152	7.50	(2.2)	7.67	(1.91)	6.18	(2.17)
excrement	1514	4.10	(2.37)	4.83	(2.1)	4.53	(1.89)
excuse	153	4.05	(1.41)	4.48	(2.29)	4.07	(2.1)
execution	154	2.37	(2.06)	5.71	(2.74)	4.11	(2.66)
exercise	155	7.13	(1.58)	6.84	(2.06)	5.68	(2.44)
exert	1515	5.35	(1.92)	5.19	(2.35)	6.04	(1.48)
exhale	1516	5.93	(1.78)	4.15	(1.85)	5.96	(1.53)
exhaust	1517	4.00	(1.66)	4.27	(2.2)	4.43	(1.45)
exhilaration	1518	7.43	(1.48)	7.20	(2.09)	6.67	(1.88)
exist	1519	6.68	(1.76)	5.39	(2.59)	4.79	(2.18)
expel	1520	2.77	(1.43)	5.90	(2.17)	3.29	(2)
expensive	1521	4.59	(2.16)	6.04	(1.71)	4.57	(1.89)
expert	1522	6.40	(1.85)	5.17	(2.26)	6.23	(2.5)
expire	1523	3.13	(1.41)	4.60	(2.08)	3.87	(1.81)
explosion	1524	5.18	(2.93)	7.93	(1.9)	4.46	(2.92)
extent	1525	5.20	(0.61)	3.83	(1.9)	5.00	(1.46)
extreme	1526	6.39	(1.93)	7.11	(1.97)	5.71	(1.61)
eye	1527	5.86	(1.38)	4.52	(2.15)	5.07	(1.41)
fabric	742	5.30	(1.2)	4.14	(1.98)	5.03	(1.61)
face	556	6.39	(1.6)	5.04	(2.18)	5.67	(1.58)
fact	1528	5.55	(1.68)	4.29	(1.92)	5.57	(1.71)
factory	1529	4.59	(1.8)	3.93	(1.76)	4.93	(1.98)
fad	1530	5.08	(1.74)	4.46	(1.98)	4.15	(1.83)
fail	1531	1.79	(1.74)	6.31	(2.84)	3.31	(2.77)
failure	156	1.70	(1.07)	4.95	(2.81)	2.40	(2.18)
fair	1532	6.67	(1.95)	5.40	(2.08)	5.77	(1.89)

Description	Word No.	Valence Mean(SD)		Arousal Mean(SD)		Dominance Mean (SD)	
fairy	1533	6.24	(2.01)	4.25	(1.9)	5.43	(1.79)
faith	1534	6.57	(2.16)	5.73	(2.42)	5.60	(1.85)
fake	1535	3.10	(1.49)	5.26	(2.18)	4.58	(2.16)
falcon	1536	5.79	(1.78)	5.14	(2.07)	5.21	(1.17)
fall	743	4.09	(2.21)	4.70	(2.48)	4.00	(2.15)
false	1537	3.74	(2.03)	4.40	(2.04)	4.20	(1.97)
false	744	3.27	(1.4)	3.43	(2.09)	4.10	(1.56)
falter	1538	3.77	(1.61)	5.00	(2.7)	4.00	(1.76)
fame	157	7.93	(1.29)	6.55	(2.46)	6.85	(2.14)
family	158	7.65	(1.55)	4.80	(2.71)	6.00	(1.87)
famine	1539	1.86	(1.15)	5.04	(2.43)	2.48	(1.93)
famous	745	6.98	(2.07)	5.73	(2.68)	6.32	(2.18)
fan	1540	6.36	(1.47)	4.89	(2.34)	5.33	(1.62)
fantasy	746	7.41	(1.9)	5.14	(2.82)	6.43	(2.05)
farm	557	5.53	(1.85)	3.90	(1.95)	5.59	(1.81)
farmer	1541	4.93	(1.98)	3.55	(2.06)	5.50	(1.89)
fascinate	159	7.34	(1.68)	5.83	(2.73)	6.15	(1.89)
fashion	1542	7.04	(1.73)	6.00	(2.11)	6.00	(1.85)
fat	160	2.28	(1.92)	4.81	(2.8)	4.47	(3.06)
fate	1543	5.43	(1.83)	5.36	(2.06)	4.07	(2.85)
father	161	7.08	(2.2)	5.92	(2.6)	5.63	(2.89)
fatigued	162	3.28	(1.43)	2.64	(2.19)	3.78	(1.97)
fault	747	3.43	(1.38)	4.07	(1.69)	4.02	(1.66)
favor	748	6.46	(1.52)	4.54	(1.86)	5.67	(1.76)
fawn	1544	6.06	(1.48)	3.72	(1.9)	4.84	(1.32)
fear	592	2.76	(2.12)	6.96	(2.17)	3.22	(2.2)
fearful	163	2.25	(1.18)	6.33	(2.28)	3.64	(2.18)
feat	1545	5.62	(1.63)	5.10	(1.72)	5.55	(1.8)
feeble	164	3.26	(1.47)	4.10	(2.07)	2.71	(1.64)
feeling	1546	6.23	(1.56)	5.00	(2.23)	5.62	(1.98)
feet	1547	4.60	(1.81)	3.67	(1.95)	5.60	(1.73)
female	1548	7.83	(1.42)	5.83	(2.39)	5.57	(1.55)
fence	1549	4.93	(0.59)	3.72	(1.94)	4.97	(1.52)
fender	1550	4.48	(2.03)	4.86	(2.45)	4.97	(1.55)
fern	1551	5.10	(1.12)	3.83	(1.9)	5.30	(1.44)

Description	Word No.	Valence Mean(SD)		Arousal Mean(SD)		Dominance Mean (SD)	
fervor	1552	5.00	(1.13)	4.81	(2.08)	4.44	(1.89)
festive	749	7.30	(2.26)	6.58	(2.29)	5.77	(2.34)
fetus	1553	5.21	(1.42)	4.97	(2.04)	4.97	(2.06)
feud	1554	3.07	(1.64)	6.14	(2.01)	4.13	(1.72)
fever	750	2.76	(1.64)	4.29	(2.31)	3.52	(2.15)
field	558	6.20	(1.37)	4.08	(2.41)	5.84	(1.94)
fig	1555	5.63	(1.25)	3.87	(2.29)	4.87	(1.68)
fight	751	3.76	(2.63)	7.15	(2.19)	5.27	(2.69)
film	1556	6.93	(1.7)	5.32	(1.96)	5.14	(1.74)
filth	165	2.47	(1.68)	5.12	(2.32)	3.81	(2.06)
financial	1557	4.15	(2.09)	5.70	(2.22)	3.81	(1.98)
finger	752	5.29	(1.42)	3.78	(2.42)	5.05	(1.7)
fingerprint	1558	4.87	(0.73)	4.67	(1.9)	4.50	(1.55)
finish	1559	7.80	(1.67)	5.37	(2.86)	6.93	(2.16)
fire	166	3.22	(2.06)	7.17	(2.06)	4.49	(2.49)
firefighter	1560	6.90	(1.61)	5.93	(2.16)	5.68	(2.36)
fireplace	1561	6.93	(1.76)	4.48	(2.32)	5.69	(1.97)
fireworks	513	7.55	(1.5)	6.67	(2.12)	5.51	(1.98)
first	1562	6.89	(2.22)	6.14	(2.48)	7.21	(1.93)
fish	559	6.04	(1.94)	4.00	(2.19)	6.02	(1.68)
fisherman	1563	5.94	(1.95)	4.97	(2.19)	5.46	(1.79)
fitting	1564	5.60	(1.66)	4.50	(1.82)	5.33	(1.17)
flabby	167	2.66	(1.87)	4.82	(2.81)	3.31	(1.9)
flag	753	6.02	(1.66)	4.60	(2.35)	5.50	(1.66)
flare	1565	5.70	(1.97)	5.23	(2.18)	4.93	(1.41)
flaunt	1566	5.04	(2.3)	5.70	(2.22)	5.22	(2.39)
flaw	1567	3.28	(1.44)	4.66	(1.72)	3.45	(1.66)
flea	1568	3.47	(2.06)	5.23	(2.22)	4.13	(1.63)
flee	1569	3.29	(1.94)	6.18	(2.13)	3.79	(2.08)
flight	1570	6.69	(1.38)	6.25	(1.92)	4.81	(1.49)
flirt	754	7.52	(1.19)	6.91	(1.69)	6.24	(2.33)
flock	1571	5.50	(1.22)	4.50	(1.63)	4.80	(1.27)
flood	755	3.19	(1.66)	6.00	(2.02)	3.24	(2.14)
flow	1572	5.77	(1.19)	3.60	(1.79)	5.43	(1.55)
flower	168	6.64	(1.78)	4.00	(2.44)	4.98	(2.17)

Description	Word No.	Valence Mean(SD)		Arousal Mean(SD)		Dominance Mean (SD)	
flu	1573	2.52	(2.2)	4.83	(2.18)	2.67	(1.99)
fluffy	1574	7.17	(1.56)	4.57	(2.52)	5.43	(1.14)
flute	1575	5.72	(1.44)	4.25	(1.98)	5.12	(1.07)
fly	1576	7.29	(1.76)	6.64	(2.08)	5.18	(2.54)
foam	756	6.07	(2.03)	5.26	(2.54)	5.24	(1.97)
foe	1577	3.39	(2.1)	6.00	(2.65)	5.25	(2.17)
folly	1578	4.40	(1.45)	4.30	(2.04)	4.30	(1.34)
food	514	7.65	(1.37)	5.92	(2.11)	6.18	(2.48)
foolish	1579	3.54	(1.79)	4.46	(2.06)	4.12	(1.77)
foot	757	5.02	(0.93)	3.27	(1.98)	4.98	(1.42)
football	1580	7.64	(1.75)	6.54	(2.81)	6.21	(2.26)
forbid	1581	3.13	(1.74)	5.47	(2.26)	3.57	(2.01)
forceful	1582	3.84	(1.57)	5.72	(1.82)	4.31	(2.21)
forest	1583	6.39	(1.57)	4.86	(2.01)	5.11	(2.08)
forget	1584	2.97	(1.54)	5.00	(2.05)	3.55	(1.57)
forgive	1585	6.33	(1.8)	4.89	(2.01)	4.93	(2.22)
fork	560	5.29	(0.97)	3.96	(1.94)	5.74	(1.52)
fornicate	1586	5.53	(2.73)	6.60	(1.99)	5.80	(2.02)
foul	169	2.81	(1.52)	4.93	(2.23)	4.51	(1.89)
fragrance	170	6.07	(1.97)	4.79	(2.54)	5.14	(1.91)
fraud	171	2.67	(1.66)	5.75	(2.45)	3.58	(2.5)
free	172	8.26	(1.31)	5.15	(3.04)	6.35	(2.4)
freedom	173	7.58	(2.04)	5.52	(2.72)	6.76	(2.29)
freeway	1587	6.07	(2)	5.87	(2.06)	5.50	(1.8)
freezer	1588	4.96	(1.87)	4.30	(2.27)	4.85	(1.68)
frenzy	1589	4.97	(1.4)	6.86	(1.83)	4.41	(1.97)
friend	174	7.74	(1.24)	5.74	(2.57)	6.74	(1.89)
friendly	175	8.43	(1.08)	5.11	(2.96)	5.92	(2.42)
fries	1590	6.81	(1.91)	4.97	(2.43)	5.97	(1.98)
fright	1591	3.19	(1.41)	5.77	(1.99)	4.12	(1.73)
frightened	1592	2.59	(1.5)	6.90	(2.11)	3.21	(2.29)
frigid	758	3.50	(1.85)	4.75	(2.56)	4.27	(1.98)
frog	176	5.71	(1.74)	4.54	(2.03)	5.34	(1.96)
frolic	1593	6.64	(1.83)	5.04	(2.5)	5.29	(2.09)
frosting	1594	7.23	(1.59)	5.47	(1.41)	5.53	(1.59)

Description	Word No.	Valence Mean(SD)		Arousal Mean(SD)		Dominance Mean (SD)	
frown	1595	1.87	(1.31)	4.27	(2.24)	3.70	(1.58)
fruit	1596	6.93	(1.82)	4.63	(2.31)	5.67	(2.02)
frustrated	177	2.48	(1.64)	5.61	(2.76)	3.50	(2.12)
frustration	1597	2.42	(1.55)	6.19	(2.58)	3.62	(2.32)
fulfilled	1598	7.27	(1.8)	5.10	(2.6)	6.86	(1.85)
fun	759	8.37	(1.11)	7.22	(2.01)	6.80	(1.85)
funeral	178	1.39	(0.87)	4.94	(3.21)	2.97	(2.55)
fungus	179	3.06	(1.75)	4.68	(2.33)	4.06	(1.94)
funny	1599	8.56	(0.85)	7.00	(1.86)	6.15	(1.92)
fur	180	4.51	(1.88)	4.18	(2.44)	4.32	(1.97)
furious	1600	1.96	(1.43)	7.64	(2.13)	5.32	(2.93)
furnace	1601	4.81	(1.49)	4.81	(1.86)	4.97	(1.12)
furniture	1602	5.93	(1.68)	4.17	(2.42)	5.52	(1.45)
fury	1603	3.10	(1.82)	6.82	(2.23)	4.29	(2.37)
fuse	1604	5.45	(1.24)	4.97	(2.01)	5.52	(1.35)
future	1605	6.71	(2.12)	6.48	(2.23)	4.89	(2.56)
gadget	1606	5.97	(1.48)	4.96	(1.69)	5.71	(1.72)
gaiety	1607	6.00	(1.41)	4.96	(2.03)	5.32	(1.63)
gain	1608	7.17	(1.32)	5.63	(1.67)	6.37	(1.87)
galaxy	1609	6.21	(1.26)	5.96	(1.71)	4.29	(2.49)
gallery	1610	6.03	(2.01)	4.07	(2.41)	4.73	(1.87)
gallon	1611	5.03	(1.47)	4.63	(1.65)	4.83	(1.39)
game	760	6.98	(1.97)	5.89	(2.37)	5.70	(1.65)
gang	1612	2.59	(1.42)	6.52	(2.49)	3.33	(2.45)
gangrene	181	2.28	(1.91)	5.70	(2.96)	3.36	(2.34)
garbage	182	2.98	(1.96)	5.04	(2.5)	4.24	(2.02)
garden	761	6.71	(1.74)	4.39	(2.35)	6.02	(1.71)
garlic	1613	5.38	(2.29)	4.45	(2.2)	5.34	(1.74)
garment	762	6.07	(1.61)	4.49	(2.5)	5.30	(1.96)
garter	534	6.22	(1.59)	5.47	(2.15)	5.82	(1.62)
gas	1614	4.43	(1.77)	5.20	(1.88)	4.60	(2.28)
gate	1615	5.52	(1.15)	4.52	(1.92)	4.73	(2.16)
gem	1616	6.65	(1.74)	5.71	(1.99)	5.35	(1.72)
gender	763	5.73	(1.55)	4.38	(2.13)	5.60	(1.84)
genius	1617	7.39	(1.45)	6.11	(1.83)	6.39	(1.99)

Description	Word No.	Valence Mean(SD)		Arousal Mean(SD)		Dominance Mean (SD)	
gentle	183	7.31	(1.3)	3.21	(2.57)	5.10	(2.16)
germ	1618	3.35	(2)	5.62	(2.33)	4.58	(2.39)
germs	764	2.86	(1.39)	4.49	(2.24)	3.79	(1.59)
ghost	1619	4.42	(1.96)	6.19	(2.23)	3.55	(1.71)
gift	184	7.77	(2.24)	6.14	(2.76)	5.52	(2.54)
giggle	1620	8.06	(1.08)	6.25	(2.13)	6.31	(1.64)
giraffe	1621	6.64	(1.42)	5.00	(2.26)	5.04	(1.4)
girl	185	6.87	(1.64)	4.29	(2.69)	5.80	(2.16)
give	1622	7.13	(1.38)	5.33	(1.88)	6.27	(1.82)
glacier	186	5.50	(1.25)	4.24	(2.29)	4.92	(2.12)
glad	1623	7.77	(1.22)	5.70	(2.17)	6.53	(1.5)
glamour	187	6.76	(1.6)	4.68	(2.23)	5.76	(2.49)
glance	1624	5.53	(1.17)	5.10	(1.79)	5.07	(1.56)
gland	1625	4.83	(1.47)	4.18	(2.18)	4.79	(1.2)
glass	765	4.75	(1.38)	4.27	(2.07)	5.00	(1.46)
glasses	1626	5.93	(1.48)	4.36	(1.85)	5.32	(1.33)
glaze	1627	6.80	(1.24)	5.10	(2.02)	5.30	(1.49)
glide	1628	6.38	(1.32)	4.82	(2.51)	5.89	(2.04)
gloat	1629	4.30	(2.11)	4.52	(2.33)	5.41	(1.78)
gloom	188	1.88	(1.23)	3.83	(2.33)	3.55	(2.07)
glory	189	7.55	(1.68)	6.02	(2.71)	6.85	(2.23)
gloss	1630	6.00	(1.58)	4.71	(1.72)	4.89	(1.52)
gnaw	1631	3.73	(1.8)	4.40	(2.21)	4.57	(1.87)
goal	1632	7.12	(1.31)	6.44	(1.96)	6.52	(2.16)
goat	1633	5.26	(1.75)	4.07	(1.84)	5.47	(1.8)
god	190	8.15	(1.27)	5.95	(2.84)	5.88	(2.89)
gold	191	7.54	(1.63)	5.76	(2.79)	5.85	(2.46)
golf	1634	4.83	(2.63)	3.90	(2.52)	4.83	(2.35)
golfer	535	5.61	(1.93)	3.73	(2.26)	5.55	(1.79)
good	766	7.47	(1.45)	5.43	(2.85)	6.41	(2.05)
goodness	1635	7.41	(1.6)	4.87	(2.45)	6.29	(1.47)
gorilla	1636	5.56	(1.95)	5.52	(2.42)	4.15	(2.6)
gossip	767	3.48	(2.33)	5.74	(2.38)	3.57	(2.26)
govern	1637	4.50	(1.43)	4.53	(1.41)	4.33	(1.73)
government	1638	4.28	(1.89)	5.25	(2)	3.62	(2.08)

Description	Word No.	Valence Mean(SD)		Arousal Mean(SD)		Dominance Mean (SD)	
grace	1639	7.00	(1.59)	4.12	(2.12)	5.62	(2.02)
graduate	192	8.19	(1.13)	7.25	(2.25)	6.94	(2.44)
grafitti	1640	4.82	(2.11)	5.32	(1.83)	5.11	(2.13)
grapes	1641	6.61	(2.25)	4.67	(2.56)	5.40	(2.19)
grass	768	6.12	(1.44)	4.14	(2.11)	5.44	(1.36)
grateful	193	7.37	(0.97)	4.58	(2.14)	6.18	(1.77)
grave	1642	2.18	(1.54)	4.78	(2.85)	2.52	(1.89)
gravy	1643	6.00	(1.74)	4.63	(1.96)	5.43	(1.48)
greed	769	3.51	(1.93)	4.71	(2.26)	4.88	(2.03)
green	194	6.18	(2.05)	4.28	(2.46)	4.82	(2.05)
greet	770	7.00	(1.52)	5.27	(2.31)	5.95	(2.07)
grenade	771	3.60	(1.88)	5.70	(2.52)	4.29	(2.5)
greyhound	1644	5.42	(1.78)	4.39	(1.65)	4.81	(1.87)
grief	195	1.69	(1.04)	4.78	(2.84)	3.50	(2.35)
grieve	1645	2.27	(1.36)	5.60	(2.39)	2.90	(1.93)
grime	772	3.37	(1.34)	3.98	(2.29)	4.47	(1.28)
grin	773	7.40	(1.87)	5.27	(2.64)	6.00	(1.86)
grind	1646	5.08	(1.92)	5.27	(2.27)	5.12	(1.56)
gripe	774	3.14	(1.56)	5.00	(2.19)	4.67	(1.79)
groceries	1647	5.90	(1.75)	3.77	(2.25)	6.23	(2.16)
groom	1648	6.53	(1.8)	5.23	(2.34)	5.90	(1.95)
gross	1649	3.57	(1.93)	4.82	(2.34)	4.36	(1.89)
ground	1650	5.36	(0.95)	4.14	(1.78)	5.14	(1.65)
grove	1651	5.60	(1.28)	4.47	(1.48)	5.23	(0.97)
grovel	1652	3.50	(1.77)	4.46	(2.35)	4.00	(2.51)
growl	1653	4.11	(2.06)	5.93	(1.9)	5.52	(2.74)
guaranteed	1654	7.14	(1.94)	4.93	(2.64)	6.39	(2.11)
guillotine	196	2.48	(2.11)	6.56	(2.54)	4.64	(2.63)
guilt	1655	2.14	(1.33)	5.36	(2.95)	2.96	(1.83)
guilty	197	2.63	(1.98)	6.04	(2.76)	3.09	(2.22)
guitar	1656	6.86	(1.66)	5.82	(2.52)	5.29	(1.8)
gulf	1657	5.90	(1.76)	4.93	(1.73)	5.48	(1.6)
gun	593	3.47	(2.48)	7.02	(1.84)	3.53	(2.72)
guzzle	1658	4.77	(1.72)	5.23	(1.92)	4.77	(1.57)
gym	1659	6.21	(1.87)	6.14	(1.96)	5.89	(1.95)

Description	Word No.	Valence Mean(SD)		Arousal Mean(SD)		Dominance Mean (SD)	
gymnast	515	6.35	(1.79)	5.02	(2.2)	5.31	(1.79)
habit	775	4.11	(1.77)	3.95	(2.11)	4.30	(1.79)
hair	1660	5.56	(1.95)	4.48	(2.36)	5.41	(2.06)
hairdryer	561	4.84	(0.84)	3.71	(1.75)	5.57	(1.27)
hairpin	776	5.26	(1.45)	3.27	(2.41)	5.05	(1.32)
ham	1661	5.31	(1.93)	4.07	(1.94)	4.96	(1.32)
hamburger	777	6.27	(1.5)	4.55	(2.14)	5.32	(1.21)
hammer	198	4.88	(1.16)	4.58	(2.02)	4.75	(1.88)
hand	778	5.95	(1.38)	4.40	(2.07)	5.35	(1.49)
handicap	779	3.29	(1.69)	3.81	(2.27)	4.00	(2.24)
handicapped	1662	3.23	(1.56)	4.55	(1.89)	3.40	(1.85)
hands	1663	5.93	(1.51)	5.21	(1.87)	5.74	(1.26)
handsome	199	7.93	(1.47)	5.95	(2.73)	5.19	(2.22)
hang	1664	4.33	(2.01)	5.40	(2.42)	4.33	(1.79)
haphazard	780	4.02	(1.41)	4.07	(2.18)	4.29	(1.67)
happiness	1665	7.96	(1.4)	6.56	(2.21)	6.70	(1.64)
happy	200	8.21	(1.82)	6.49	(2.77)	6.63	(2.43)
harass	1666	2.48	(1.18)	5.64	(2.28)	3.93	(2.62)
harassment	1667	2.41	(1.62)	5.75	(2.55)	3.16	(2.08)
hard	781	5.22	(1.82)	5.12	(2.19)	5.59	(1.63)
hardship	782	2.45	(1.61)	4.76	(2.55)	4.22	(2.4)
harp	1668	6.37	(1.61)	4.41	(2.5)	4.86	(1.71)
harsh	1669	2.93	(1.64)	5.60	(1.85)	4.83	(2.28)
harvest	1670	5.74	(1.55)	4.37	(1.79)	5.76	(1.5)
haste	1671	4.15	(1.7)	5.04	(2.49)	4.19	(1.86)
hasten	1672	4.90	(0.99)	4.90	(1.95)	4.67	(1.52)
hat	783	5.46	(1.36)	4.10	(2)	5.39	(1.43)
hate	201	2.12	(1.72)	6.95	(2.56)	5.05	(2.95)
hatred	202	1.98	(1.92)	6.66	(2.56)	4.30	(2.76)
haunt	1673	3.48	(1.96)	6.60	(1.94)	3.72	(1.9)
haven	1674	6.97	(1.92)	4.00	(2.38)	5.37	(2.09)
hawk	536	5.88	(1.62)	4.39	(2.29)	5.50	(1.69)
hay	784	5.24	(1.24)	3.95	(2.58)	5.37	(1.64)
head	1675	6.63	(1.43)	5.70	(2.2)	6.03	(1.56)
headache	203	2.02	(1.06)	5.07	(2.74)	3.60	(1.98)

Description	Word No.	Valence Mean(SD)		Arousal Mean(SD)		Dominance Mean (SD)	
headlight	785	5.24	(1.51)	3.81	(2.22)	4.88	(1.47)
heal	786	7.09	(1.46)	4.77	(2.23)	5.79	(1.8)
health	204	6.81	(1.88)	5.13	(2.35)	5.83	(1.91)
heart	787	7.39	(1.53)	6.34	(2.25)	5.49	(2.11)
heat	1676	4.30	(1.95)	5.70	(2.02)	4.17	(1.86)
heaven	205	7.30	(2.39)	5.61	(3.2)	6.15	(2.56)
heavy	1677	3.69	(1.38)	4.58	(1.93)	4.10	(1.62)
heir	1678	6.57	(1.55)	5.22	(1.95)	5.78	(1.95)
helium	1679	5.29	(2.17)	4.71	(2.55)	4.85	(1.96)
hell	788	2.24	(1.62)	5.38	(2.62)	3.24	(2.36)
helpless	206	2.20	(1.42)	5.34	(2.52)	2.27	(1.83)
hen	1680	5.10	(0.98)	3.79	(1.9)	5.10	(1.63)
herbal	1681	5.92	(1.29)	3.62	(1.97)	5.29	(1)
herd	1682	5.00	(1.29)	4.50	(1.8)	4.73	(1.74)
hero	1683	7.59	(1.72)	6.41	(1.8)	5.90	(1.95)
heroin	789	4.36	(2.73)	5.11	(2.72)	4.80	(2.54)
herring	1684	5.43	(1.25)	4.37	(1.71)	5.20	(1.16)
hide	207	4.32	(1.91)	5.28	(2.51)	3.40	(2.12)
high	1685	6.62	(1.98)	5.56	(2.09)	5.38	(1.84)
highway	562	5.92	(1.72)	5.16	(2.44)	5.66	(1.81)
hiker	1686	5.79	(1.74)	5.00	(2.16)	4.68	(2.29)
hill	1687	5.32	(1.41)	3.75	(2.11)	5.00	(1.64)
hinder	790	3.81	(1.42)	4.12	(2.01)	4.21	(1.54)
hippotamus	1688	6.41	(1.62)	4.36	(2.15)	4.93	(1.56)
hire	1689	5.77	(1.67)	5.63	(2.04)	4.80	(1.79)
history	208	5.24	(2.01)	3.93	(2.29)	4.83	(2.08)
hit	594	4.33	(2.35)	5.73	(2.09)	4.88	(2.01)
hitch	1690	5.03	(1.21)	4.25	(2.17)	5.00	(1.15)
hive	1691	3.18	(1.52)	5.82	(2.02)	4.21	(1.95)
hobby	1692	7.24	(1.48)	5.36	(2.59)	7.24	(1.83)
hockey	1693	5.39	(1.45)	5.00	(2.21)	5.18	(1.49)
holiday	791	7.55	(2.14)	6.59	(2.73)	6.30	(2.17)
holy	1694	6.36	(1.93)	5.36	(2.15)	4.68	(2.25)
home	209	7.91	(1.63)	4.21	(2.94)	5.90	(2.3)
homeless	1695	2.06	(1.13)	4.59	(2.28)	3.31	(1.99)

Description	Word No.	Valence Mean(SD)		Arousal Mean(SD)		Dominance Mean (SD)	
honest	210	7.70	(1.43)	5.32	(1.92)	6.24	(2.13)
honesty	1696	7.88	(1.24)	5.25	(2.45)	6.88	(1.5)
honey	792	6.73	(1.7)	4.51	(2.25)	5.44	(1.47)
honor	211	7.66	(1.24)	5.90	(1.83)	6.70	(2.04)
hooker	793	3.34	(2.31)	4.93	(2.82)	4.73	(2.48)
hope	794	7.05	(1.96)	5.44	(2.47)	5.52	(2.2)
hopeful	212	7.10	(1.46)	5.78	(2.09)	5.41	(1.92)
horrible	1697	2.28	(1.98)	5.75	(2.41)	3.04	(1.95)
horror	213	2.76	(2.25)	7.21	(2.14)	4.63	(2.7)
horse	214	5.89	(1.55)	3.89	(2.17)	4.67	(1.6)
hose	1698	5.25	(1.6)	4.82	(2.09)	5.36	(2.02)
hospital	215	5.04	(2.45)	5.98	(2.54)	4.69	(2.16)
hostage	216	2.20	(1.8)	6.76	(2.63)	2.83	(2.32)
hostile	217	2.73	(1.5)	6.44	(2.28)	4.85	(2.58)
hotel	795	6.00	(1.77)	4.80	(2.53)	5.12	(1.84)
hour	1699	5.10	(1.23)	4.21	(1.77)	4.25	(1.71)
house	563	7.26	(1.72)	4.56	(2.41)	6.08	(2.12)
howl	1700	4.90	(1.88)	5.27	(2.48)	4.43	(1.87)
hub	1701	5.52	(1.58)	4.00	(2.11)	5.07	(1.71)
hug	218	8.00	(1.55)	5.35	(2.76)	5.79	(2.41)
huge	1702	5.39	(1.97)	5.75	(2.14)	5.68	(2.5)
humane	796	6.89	(1.7)	4.50	(1.91)	5.70	(1.91)
humble	219	5.86	(1.42)	3.74	(2.33)	4.76	(2.25)
humiliate	797	2.24	(1.34)	6.14	(2.42)	2.60	(1.94)
humiliation	1703	1.90	(1.27)	6.97	(1.97)	2.20	(1.42)
humor	220	8.56	(0.81)	5.50	(2.91)	6.08	(2.14)
hunger	1704	2.76	(1.38)	5.36	(2.31)	3.93	(1.65)
hungry	221	3.58	(2.01)	5.13	(2.44)	4.68	(2.05)
hunter	1705	4.90	(2.4)	5.86	(2.4)	5.70	(2.48)
hurl	1706	3.12	(1.72)	5.40	(1.76)	4.36	(2.14)
hurricane	798	3.34	(2.12)	6.83	(2.06)	3.07	(2.18)
hurt	222	1.90	(1.26)	5.85	(2.49)	3.33	(2.22)
hydrant	564	5.02	(0.93)	3.71	(1.75)	5.53	(1.3)
hymn	1707	6.13	(2.08)	4.33	(2.73)	5.30	(1.82)
hysterical	1708	5.29	(3.31)	7.36	(2.04)	4.18	(2.39)

Description	Word No.	Valence Mean(SD)		Arousal Mean(SD)		Dominance Mean (SD)	
ice	1709	5.92	(1.55)	4.67	(2.28)	5.04	(1.4)
icebox	799	4.95	(1)	4.17	(2.11)	5.05	(1.05)
idea	800	7.00	(1.34)	5.86	(1.81)	6.26	(2)
identity	801	6.57	(1.99)	4.95	(2.24)	6.40	(1.89)
idiot	223	3.16	(1.91)	4.21	(2.47)	3.18	(2.13)
idol	802	6.12	(1.86)	4.95	(2.14)	5.37	(2.17)
ignite	1710	6.16	(2.03)	6.27	(1.87)	5.87	(1.76)
ignorance	803	3.07	(2.25)	4.39	(2.49)	4.41	(2.38)
ignore	1711	3.15	(1.96)	4.67	(2.37)	3.56	(2.45)
illness	804	2.48	(1.4)	4.71	(2.24)	3.21	(1.85)
imagine	805	7.32	(1.52)	5.98	(2.14)	7.07	(1.99)
immature	806	3.39	(1.7)	4.15	(1.96)	4.85	(2.2)
immoral	807	3.50	(2.16)	4.98	(2.48)	4.66	(2.33)
impair	808	3.18	(1.86)	4.04	(2.14)	4.09	(2.18)
impart	1712	4.97	(1.12)	3.86	(2.03)	5.45	(1.59)
impatient	1713	3.63	(1.9)	5.57	(2.47)	4.23	(2.25)
impede	1714	4.19	(1.74)	4.90	(1.99)	3.90	(1.63)
impose	1715	3.66	(1.59)	5.28	(1.79)	4.45	(2.06)
impotent	224	2.81	(1.92)	4.57	(2.59)	3.43	(2.43)
impressed	225	7.33	(1.84)	5.42	(2.65)	5.51	(2.21)
improve	226	7.65	(1.16)	5.69	(2.15)	6.08	(2.25)
incentive	809	7.00	(1.72)	5.69	(2.45)	5.93	(2.02)
incubator	1716	4.17	(1.98)	4.86	(1.94)	4.17	(1.93)
indict	1717	4.29	(1.56)	4.93	(2.21)	4.36	(2.09)
indifferent	810	4.61	(1.28)	3.18	(1.85)	4.84	(1.67)
industry	227	5.30	(1.61)	4.47	(2.43)	4.91	(2.04)
infant	811	6.95	(2.08)	5.05	(2.66)	5.67	(2.48)
infatuated	1718	5.77	(1.77)	6.27	(2.09)	4.77	(1.9)
infatuation	516	6.73	(2.08)	7.02	(1.87)	4.90	(2.28)
infect	1719	2.42	(1.17)	5.38	(2.25)	3.42	(1.96)
infection	228	1.66	(1.34)	5.03	(2.77)	3.61	(2.64)
inferior	812	3.07	(1.57)	3.83	(2.05)	2.78	(2.08)
infest	1720	2.85	(1.41)	5.19	(1.9)	3.62	(1.65)
inform	1721	5.92	(0.95)	4.57	(2.11)	5.26	(1.66)
ingest	1722	5.00	(1.64)	5.27	(1.74)	5.03	(1.85)

Description	Word No.	Valence Mean(SD)		Arousal Mean(SD)		Dominance Mean (SD)	
inhabitant	813	5.05	(1.34)	3.95	(1.97)	5.37	(1.43)
inject	1723	3.34	(1.74)	5.54	(2.43)	3.89	(2.1)
injection	1724	3.03	(1.59)	6.55	(1.8)	3.48	(1.81)
injure	1725	2.55	(1.89)	5.53	(2.27)	3.86	(2.33)
injury	595	2.49	(1.76)	5.69	(2.06)	3.57	(1.62)
ink	229	5.05	(0.81)	3.84	(1.88)	4.61	(2.13)
innocence	1726	6.54	(1.95)	4.79	(2.42)	5.75	(2.29)
innocent	814	6.51	(1.34)	4.21	(1.99)	5.28	(2.08)
insane	815	2.85	(1.94)	5.83	(2.45)	4.12	(2.23)
insect	816	4.07	(2.16)	4.07	(2.46)	4.56	(2.47)
insecure	230	2.36	(1.33)	5.56	(2.34)	2.33	(1.95)
insist	1727	4.64	(1.95)	5.18	(2.2)	4.93	(2.02)
insolent	231	4.35	(1.76)	5.38	(2.37)	4.50	(2.06)
inspire	232	6.97	(1.91)	5.00	(2.53)	6.34	(2.11)
inspired	233	7.15	(1.85)	6.02	(2.67)	6.67	(2.31)
instrument	1728	6.68	(1.74)	5.32	(2.48)	5.71	(2.03)
insult	817	2.29	(1.33)	6.00	(2.46)	3.62	(2.05)
intellect	818	6.82	(1.96)	4.75	(2.5)	6.30	(1.98)
intelligence	1729	7.93	(1.36)	6.21	(2.04)	6.25	(2.17)
intent	1730	5.00	(1.41)	4.73	(1.66)	4.96	(1.71)
intercourse	819	7.36	(1.57)	7.00	(2.07)	6.40	(1.78)
interest	234	6.97	(1.54)	5.66	(2.26)	5.88	(1.78)
interested	1731	6.58	(1.45)	5.42	(2.37)	5.95	(1.68)
intimate	821	7.61	(1.51)	6.98	(2.21)	5.86	(2.29)
intoxicated	1732	5.00	(2.54)	6.19	(2.18)	4.28	(2.4)
intruder	822	2.77	(2.32)	6.86	(2.41)	4.00	(2.68)
invade	1733	3.41	(1.66)	6.00	(2.42)	4.25	(2.68)
invader	823	3.05	(2.01)	5.50	(2.4)	4.00	(2.6)
invent	1734	6.73	(2.07)	6.20	(2.04)	6.47	(2.26)
invest	824	5.93	(2.1)	5.12	(2.42)	5.88	(1.95)
iron	565	4.90	(1.02)	3.76	(2.06)	5.10	(1.27)
irony	1735	6.17	(1.67)	4.66	(2.21)	4.83	(1.79)
irritate	235	3.11	(1.67)	5.76	(2.15)	5.03	(2.05)
irritated	1736	2.23	(1.55)	6.13	(2.24)	4.37	(1.97)
island	1737	7.59	(1.8)	6.14	(2.18)	6.21	(1.78)

Description	Word No.	Valence Mean(SD)		Arousal Mean(SD)		Dominance Mean (SD)	
isolation	1738	2.17	(1.6)	4.70	(2.67)	2.60	(1.65)
issue	1739	4.13	(1.57)	5.40	(2.24)	4.63	(1.87)
item	825	5.26	(0.86)	3.24	(2.08)	5.26	(1.67)
ivy	1740	4.11	(1.71)	5.11	(2.11)	4.67	(1.84)
jaguar	1741	6.62	(1.78)	5.50	(2.27)	4.79	(2.15)
jail	236	1.95	(1.27)	5.49	(2.67)	3.81	(2.71)
jar	1742	5.21	(0.56)	3.75	(2.17)	5.75	(1.48)
jealous	1743	2.86	(1.73)	6.48	(2.18)	3.72	(2.3)
jealousy	237	2.51	(1.83)	6.36	(2.66)	3.80	(2.41)
jeans	1744	6.43	(1.77)	4.74	(2.49)	5.89	(1.67)
jelly	238	5.66	(1.44)	3.70	(2.29)	4.53	(1.77)
jellyfish	1745	4.76	(2.47)	6.04	(2.2)	4.29	(2.05)
jeopardy	1746	6.45	(1.57)	5.18	(2.45)	4.93	(1.86)
jerk	1747	2.86	(1.66)	5.21	(2.25)	4.64	(1.93)
jet	1748	6.29	(1.7)	5.89	(2.04)	4.89	(2.01)
jewel	239	7.00	(1.72)	5.38	(2.54)	5.59	(2.19)
job	1749	5.83	(2.15)	5.20	(2.23)	5.70	(1.82)
joke	826	8.10	(1.36)	6.74	(1.84)	6.15	(1.86)
jolly	827	7.41	(1.92)	5.57	(2.8)	6.39	(1.72)
journal	828	5.14	(1.49)	4.05	(1.96)	5.26	(1.42)
joy	240	8.60	(0.71)	7.22	(2.13)	6.28	(2.15)
joyful	241	8.22	(1.22)	5.98	(2.54)	6.60	(1.8)
judge	1750	4.53	(1.7)	5.10	(2.02)	3.67	(1.9)
judgment	1751	4.41	(1.78)	5.78	(1.91)	4.38	(2.2)
jug	829	5.24	(1.65)	3.88	(2.15)	5.05	(1.62)
jugs	1752	5.65	(1.98)	5.23	(2.41)	5.00	(1.6)
juice	1753	6.79	(1.88)	5.69	(1.54)	5.66	(1.23)
jumble	1754	4.41	(1.89)	5.30	(2.35)	4.30	(1.98)
jungle	1755	5.77	(2.05)	5.85	(1.49)	4.81	(1.55)
justice	242	7.78	(1.35)	5.47	(2.54)	6.47	(2.26)
keg	1756	6.21	(2.23)	5.37	(2.1)	5.33	(1.92)
kerchief	830	5.11	(1.33)	3.43	(2.08)	5.25	(1.28)
kerosene	243	4.80	(1.59)	4.34	(2.51)	4.63	(1.99)
ketchup	831	5.60	(1.35)	4.09	(2.08)	5.29	(1.81)
kettle	832	5.22	(0.91)	3.22	(2.23)	5.00	(1.4)
key	833	5.68	(1.62)	3.70	(2.18)	4.98	(2.04)
kick	834	4.31	(2.18)	4.90	(2.35)	5.50	(1.93)
kid	1757	6.42	(1.61)	5.35	(2.06)	6.03	(2.14)
kidnap	1758	2.62	(1.83)	6.31	(2.43)	3.08	(2.59)
kidney	1759	4.86	(1.92)	4.48	(2.29)	4.22	(1.97)
kids	835	6.91	(1.99)	5.27	(2.36)	5.07	(2.03)
killer	244	1.89	(1.39)	7.86	(1.89)	4.54	(3.11)
kind	245	7.59	(1.67)	4.46	(2.55)	5.95	(1.93)
kindness	246	7.82	(1.39)	4.30	(2.62)	5.67	(2.63)
king	247	7.26	(1.67)	5.51	(2.77)	7.38	(2.1)
kiss	248	8.26	(1.54)	7.32	(2.03)	6.93	(2.28)
kitten	517	6.86	(2.13)	5.08	(2.45)	6.86	(2.01)
knee	1760	5.03	(1.1)	4.40	(1.67)	5.20	(1.37)
knife	596	3.62	(2.18)	5.80	(2)	4.12	(2.18)
knitting	1761	4.94	(2.44)	2.88	(2.27)	5.41	(2)
knob	1762	5.14	(1.19)	4.00	(1.98)	5.29	(1.12)
knot	836	4.64	(1.36)	4.07	(2.15)	4.67	(1.65)
know	1763	6.93	(1.72)	5.77	(1.92)	6.90	(2.29)
knowledge	249	7.58	(1.32)	5.92	(2.32)	6.78	(2.41)
label	1764	4.36	(1.08)	3.96	(1.99)	4.83	(1.76)
lack	1765	3.21	(1.37)	4.21	(2.47)	4.11	(1.97)
lad	1766	5.44	(1.13)	4.06	(1.54)	5.28	(1.37)
ladder	1767	5.06	(1.09)	4.97	(2.12)	5.10	(1.45)
lake	250	6.82	(1.54)	3.95	(2.44)	4.90	(2.1)
lamb	837	5.89	(1.73)	3.36	(2.18)	4.91	(1.96)
lame	1768	3.66	(1.82)	3.69	(2)	4.83	(1.63)
lamp	838	5.41	(1)	3.80	(2.12)	5.27	(1.61)
land	1769	5.66	(1.42)	4.16	(2.11)	5.53	(1.59)
lane	1770	5.39	(1.42)	4.64	(1.79)	5.54	(1.2)
lantern	839	5.57	(1.19)	4.05	(2.28)	5.07	(1.82)
laugh	1771	8.36	(1.13)	7.39	(2.01)	6.64	(1.87)
laughter	251	8.45	(1.08)	6.75	(2.5)	6.45	(2.45)
lava	1772	4.43	(2.15)	5.57	(2.64)	3.54	(1.99)
lavish	840	6.21	(2.03)	4.93	(2.4)	5.64	(1.61)
law	1773	5.10	(2.37)	4.25	(2.43)	4.11	(2.08)

Description	Word No.	Valence Mean(SD)		Arousal Mean(SD)		Dominance Mean (SD)	
lawn	841	5.24	(0.86)	4.00	(1.79)	5.37	(1.11)
lawsuit	842	3.37	(2)	4.93	(2.44)	3.92	(2.02)
lawyer	1774	5.70	(2.27)	5.85	(2.61)	4.89	(2.81)
lazy	843	4.38	(2.02)	2.65	(2.06)	4.07	(1.93)
leader	844	7.63	(1.59)	6.27	(2.18)	7.88	(1.6)
leak	1775	3.07	(1.41)	4.63	(1.96)	3.87	(1.68)
learn	252	7.15	(1.49)	5.39	(2.22)	6.34	(2.17)
lease	1776	4.45	(1.36)	4.70	(1.7)	4.70	(1.56)
leave	1777	3.83	(1.44)	4.36	(1.91)	4.36	(2)
leaves	1778	6.07	(1.93)	3.80	(2.44)	4.80	(2.06)
ledge	1779	4.13	(1.31)	5.35	(1.96)	4.10	(1.37)
leg	1780	5.71	(1.12)	4.50	(2.12)	5.36	(1.66)
legend	845	6.39	(1.34)	4.88	(1.76)	5.54	(1.64)
legs	1781	5.93	(1.36)	4.69	(2.35)	5.97	(1.67)
leisurely	253	6.88	(1.81)	3.80	(2.38)	5.15	(1.9)
leopard	1782	5.97	(1.69)	5.57	(2.18)	4.57	(1.7)
leprosy	254	2.09	(1.4)	6.29	(2.23)	4.00	(2.3)
lesbian	597	4.67	(2.45)	5.12	(2.27)	5.35	(2.2)
lesson	1783	5.07	(1.63)	4.04	(1.75)	5.04	(1.48)
letter	846	6.61	(1.59)	4.90	(2.37)	5.73	(1.48)
liar	1784	2.41	(1.64)	6.38	(2.47)	3.34	(2.42)
liberty	255	7.98	(1.22)	5.60	(2.65)	6.29	(2.44)
lice	256	2.31	(1.78)	5.00	(2.26)	3.95	(2.29)
lick	1785	6.27	(1.51)	5.70	(2.12)	5.90	(1.49)
lid	1786	5.03	(0.5)	3.66	(1.88)	5.14	(1.62)
lie	257	2.79	(1.92)	5.96	(2.63)	3.30	(2.42)
life	258	7.27	(1.88)	6.02	(2.62)	5.72	(2.51)
lightbulb	566	5.61	(1.28)	4.10	(2.02)	5.82	(1.56)
lighthouse	847	5.89	(2.08)	4.41	(2.44)	5.25	(2.02)
lightning	598	4.57	(2.66)	6.61	(1.77)	3.67	(2.19)
like	1787	7.52	(1.72)	6.63	(2.2)	5.41	(2.69)
limb	1788	5.03	(0.81)	4.53	(1.53)	5.30	(1.12)
limber	848	5.68	(1.49)	4.57	(2.26)	5.34	(1.84)
limp	1789	3.71	(1.88)	4.29	(1.78)	4.43	(2.18)
linger	1790	4.44	(1.39)	4.92	(1.63)	5.12	(1.69)
lion	518	5.57	(1.99)	6.20	(2.16)	4.12	(2.33)
lips	1791	7.13	(1.5)	6.27	(1.87)	5.60	(2.11)
liquor	1792	4.93	(2.43)	5.66	(2.19)	4.86	(2.33)
listen	1793	5.93	(1.16)	4.45	(1.7)	5.76	(1.46)
listless	259	4.12	(1.73)	4.10	(2.31)	4.14	(1.73)
liter	1794	5.00	(1.74)	4.33	(2.18)	5.37	(1.84)
litter	1795	3.96	(2.22)	4.77	(1.95)	4.85	(1.76)
lively	849	7.20	(1.97)	5.53	(2.9)	6.09	(1.95)
lizard	1796	5.23	(1.83)	5.13	(1.57)	4.97	(1.5)
location	1797	5.36	(0.68)	4.11	(1.83)	5.39	(1.34)
locker	850	5.19	(1.31)	3.38	(2.13)	5.36	(1.87)
loneliness	260	1.61	(1.02)	4.56	(2.97)	2.51	(2.27)
lonely	261	2.17	(1.76)	4.51	(2.68)	2.95	(2.12)
loosen	1798	5.59	(1.76)	4.50	(2.41)	6.11	(2.11)
lose	1799	2.81	(1.64)	4.93	(2.42)	3.33	(2.17)
loser	851	2.25	(1.48)	4.95	(2.57)	3.02	(2.17)
loss	1800	1.89	(1.25)	5.78	(2.95)	2.38	(2.3)
lost	852	2.82	(1.83)	5.82	(2.62)	2.86	(1.64)
lotion	1801	5.90	(2.04)	5.52	(2.21)	5.70	(1.86)
lottery	853	6.57	(2.04)	5.36	(2.45)	4.81	(2.11)
loud	1802	4.77	(1.73)	6.10	(1.6)	5.07	(1.44)
lounge	1803	6.60	(1.38)	3.97	(2.33)	5.90	(1.54)
louse	262	2.81	(1.92)	4.98	(2.03)	3.57	(2.26)
love	263	8.72	(0.7)	6.44	(3.35)	7.11	(2.56)
loved	264	8.64	(0.71)	6.38	(2.68)	6.62	(2.53)
loving	1804	8.28	(0.88)	6.21	(2.45)	6.29	(1.9)
lower	1805	4.00	(1.58)	4.36	(1.91)	4.18	(1.63)
loyal	265	7.55	(1.9)	5.16	(2.42)	6.91	(2.23)
luck	1806	7.41	(1.46)	6.45	(1.73)	4.97	(2.37)
lucky	266	8.17	(1.06)	6.53	(2.34)	6.05	(2.25)
luggage	1807	6.33	(1.4)	5.70	(1.6)	5.23	(1.77)
lump	854	4.16	(2.34)	4.80	(2.82)	4.32	(2.18)
lunch	1808	7.21	(1.84)	5.43	(2.46)	6.10	(2.4)
lung	1809	5.21	(1.32)	4.50	(1.73)	4.64	(1.62)
lurk	1810	4.20	(1.54)	5.73	(1.96)	4.00	(1.86)

Description	Word No.	Valence Mean(SD)		Arousal Mean(SD)		Dominance Mean (SD)	
luscious	267	7.50	(1.08)	5.34	(2.51)	5.68	(1.84)
lust	519	7.12	(1.62)	6.88	(1.85)	5.49	(2.27)
luxury	268	7.88	(1.49)	4.75	(2.91)	6.40	(2.45)
machine	855	5.09	(1.67)	3.82	(2.4)	5.23	(2.06)
mad	856	2.44	(1.72)	6.76	(2.26)	5.86	(2.2)
madman	857	3.91	(2.49)	5.56	(2.78)	4.79	(2.55)
madness	1811	4.37	(2.19)	5.80	(2.62)	4.53	(2.33)
magazine	1812	5.79	(1.7)	4.82	(1.98)	4.93	(1.84)
maggot	269	2.06	(1.47)	5.28	(2.96)	4.03	(2.09)
magical	858	7.46	(1.64)	5.95	(2.36)	5.73	(2.19)
magnet	1813	5.31	(0.93)	4.72	(2.02)	5.21	(1.63)
maid	1814	5.56	(1.36)	5.20	(1.8)	5.72	(1.49)
mail	859	6.88	(1.74)	5.63	(2.36)	5.67	(1.79)
makeup	1815	5.64	(2.09)	4.48	(2.39)	5.59	(2.08)
malaria	860	2.40	(1.38)	4.40	(2.54)	3.22	(1.9)
male	1816	5.84	(1.7)	4.90	(2.7)	5.32	(1.68)
malice	270	2.69	(1.84)	5.86	(2.75)	4.74	(2.72)
malnutrition	1817	2.06	(1.27)	5.68	(2.26)	4.00	(2.13)
malt	1818	6.17	(1.69)	4.83	(2.24)	5.38	(1.82)
man	537	6.73	(1.7)	5.24	(2.31)	5.53	(2.23)
mane	1819	5.40	(1.38)	4.33	(2.2)	4.90	(1.52)
mangle	861	3.90	(2.01)	5.44	(2.1)	4.61	(1.84)
maniac	862	3.76	(2)	5.39	(2.46)	4.22	(2.07)
manner	863	5.64	(1.34)	4.56	(1.78)	5.05	(1.83)
manor	1820	5.61	(1.1)	4.74	(1.68)	5.41	(1.19)
mansion	1821	7.24	(1.99)	6.03	(2.13)	5.86	(1.78)
mantel	864	4.93	(1.4)	3.27	(2.23)	4.95	(1.61)
manure	865	3.10	(1.74)	4.17	(2.09)	4.67	(1.36)
map	1822	5.14	(1.64)	4.07	(1.75)	5.28	(2.12)
margin	1823	4.79	(0.73)	3.61	(1.87)	4.85	(1.03)
market	866	5.66	(1.02)	4.12	(1.83)	5.27	(1.4)
marrow	1824	4.46	(1.5)	4.26	(1.93)	4.41	(1.62)
marry	1825	6.68	(2.36)	6.14	(2.76)	5.18	(2.54)
marsh	1826	4.67	(1.44)	4.15	(2.18)	4.67	(1.24)
marvel	1827	7.06	(1.77)	5.20	(2.31)	4.93	(2.07)

Description	Word No.	Valence Mean(SD)		Arousal Mean(SD)		Dominance Mean (SD)	
mascot	1828	6.29	(1.6)	5.10	(2.61)	5.39	(1.93)
mask	1829	5.23	(1.31)	4.58	(1.75)	5.42	(1.72)
massacre	867	2.28	(1.74)	5.33	(2.63)	3.50	(2.26)
mastectomy	1830	2.89	(1.81)	5.11	(2.17)	3.75	(1.62)
masterful	271	7.09	(1.78)	5.20	(2.85)	7.18	(2.56)
mastery	1831	6.69	(1.61)	5.62	(1.92)	7.28	(1.67)
masturbate	599	5.45	(2.02)	5.67	(2.18)	5.63	(2.25)
matador	1832	5.10	(1.82)	5.57	(1.93)	4.89	(1.45)
mate	1833	6.87	(1.74)	6.30	(2.34)	5.48	(2.2)
material	868	5.26	(1.29)	4.05	(2.34)	5.12	(1.45)
math	1834	3.81	(2.13)	4.62	(2.57)	4.44	(2.34)
me	1835	8.06	(1.29)	5.97	(2.66)	7.88	(1.6)
meal	1836	7.59	(1.43)	5.79	(1.97)	6.41	(1.66)
measles	272	2.74	(1.97)	5.06	(2.44)	4.13	(2.16)
meat	1837	6.66	(1.8)	5.41	(1.97)	6.03	(1.61)
meddle	1838	4.07	(2)	5.10	(1.88)	4.87	(2)
medicine	869	5.67	(2.06)	4.40	(2.36)	4.70	(1.91)
meek	273	3.87	(1.69)	3.80	(2.13)	3.67	(2.23)
melody	870	7.07	(1.79)	4.98	(2.52)	5.46	(1.78)
melt	1839	5.28	(1.94)	4.93	(1.94)	4.86	(1.9)
member	1840	5.83	(1.51)	5.03	(1.94)	5.30	(1.37)
memorial	1841	4.68	(2.14)	4.87	(2.38)	4.42	(1.57)
memories	871	7.48	(1.61)	6.10	(2.1)	5.88	(1.92)
memory	274	6.62	(1.5)	5.42	(2.25)	5.11	(2.12)
men	1842	6.66	(1.7)	5.69	(2.24)	6.17	(2.02)
menace	275	2.88	(1.64)	5.52	(2.45)	4.98	(2.25)
mend	1843	6.17	(1.67)	4.93	(2.18)	5.82	(1.81)
menu	1844	6.37	(1.71)	4.77	(2.13)	6.00	(1.78)
mercy	1845	5.71	(2.41)	5.43	(2.54)	3.70	(2.72)
merger	1846	5.06	(0.95)	4.31	(1.64)	5.16	(0.95)
merry	872	7.90	(1.49)	5.90	(2.42)	6.64	(1.66)
messy	873	3.15	(1.73)	3.34	(2.37)	4.75	(2.15)
metal	874	4.95	(1.17)	3.79	(1.96)	5.38	(1.4)
meteor	1847	5.57	(2.16)	5.83	(2.78)	3.33	(1.77)
method	875	5.56	(1.76)	3.85	(2.58)	5.67	(1.58)

Description	Word No.	Valence Mean(SD)		Arousal Mean(SD)		Dominance Mean (SD)	
midnight	1848	7.18	(1.7)	6.50	(2.1)	5.96	(1.93)
mighty	276	6.54	(2.19)	5.61	(2.38)	7.23	(2.11)
mildew	277	3.17	(1.36)	4.08	(1.79)	4.40	(1.79)
mile	1849	5.24	(2.18)	4.69	(2.12)	5.31	(2)
military	1850	5.54	(1.9)	5.35	(2)	4.65	(2.3)
milk	876	5.95	(2.16)	3.68	(2.57)	5.83	(1.5)
millionaire	278	8.03	(1.42)	6.14	(2.7)	6.97	(2.4)
mind	877	6.68	(1.84)	5.00	(2.68)	6.37	(2.19)
mingle	1851	6.66	(1.33)	5.88	(1.83)	5.97	(1.6)
miracle	279	8.60	(0.71)	7.65	(1.67)	5.35	(2.58)
mischief	878	5.57	(2.05)	5.76	(1.95)	5.56	(1.88)
miserable	1852	1.55	(1.15)	5.00	(2.68)	2.43	(1.85)
misery	879	1.93	(1.6)	5.17	(2.69)	2.55	(1.45)
missiles	1853	3.17	(1.81)	6.69	(1.91)	3.14	(2.03)
missles	600	3.33	(2.26)	5.94	(2.21)	3.25	(2.31)
mistake	880	2.86	(1.79)	5.18	(2.42)	3.86	(2.42)
mitten	1854	5.59	(1.25)	3.78	(2.24)	4.63	(1.36)
mob	1855	3.27	(1.66)	6.11	(2.1)	3.32	(2)
mobility	881	6.83	(1.79)	5.00	(2.18)	6.43	(1.48)
model	1856	7.10	(1.78)	6.00	(2.51)	4.86	(1.92)
modest	280	5.76	(1.28)	3.98	(2.24)	4.96	(2.16)
moist	1857	5.52	(1.68)	4.55	(2.38)	5.03	(1.43)
mold	882	3.55	(1.7)	4.07	(1.98)	4.33	(1.83)
molest	1858	1.90	(1.35)	5.20	(2.86)	3.23	(2.36)
moment	281	5.76	(1.65)	3.83	(2.29)	4.81	(1.92)
money	282	7.59	(1.4)	5.70	(2.66)	6.25	(2.33)
monk	1859	5.18	(1.85)	3.39	(2.18)	4.89	(1.89)
monopoly	1860	6.40	(1.94)	5.24	(1.6)	5.70	(1.95)
month	283	5.15	(1.09)	4.03	(1.77)	4.85	(1.14)
mood	1861	5.60	(1.38)	4.73	(1.78)	5.60	(1.65)
moody	883	3.20	(1.58)	4.18	(2.38)	4.39	(1.71)
moon	1862	6.74	(1.32)	4.60	(1.9)	4.80	(1.81)
moral	884	6.20	(1.85)	4.49	(2.28)	5.90	(2.2)
morale	1863	6.57	(1.75)	5.00	(2.74)	5.75	(2.01)
morbid	284	2.87	(2.14)	5.06	(2.68)	4.34	(2.5)
morgue	285	1.92	(1.32)	4.84	(2.96)	3.61	(1.94)
moron	1864	3.77	(2.13)	5.70	(1.58)	4.60	(2.24)
morsel	1865	5.47	(2.32)	4.83	(2.13)	5.17	(2.18)
mosquito	885	2.80	(1.91)	4.78	(2.72)	4.51	(2.15)
moss	1866	4.59	(1.32)	3.57	(2.03)	4.54	(1.35)
motel	1867	4.43	(2.17)	4.93	(2.14)	4.32	(1.91)
moth	1868	4.66	(1.59)	4.00	(2.19)	5.00	(1.87)
mother	286	8.39	(1.15)	6.13	(2.71)	5.74	(2.37)
motorcycle	1869	5.72	(2.68)	6.46	(2.19)	5.14	(2.21)
mountain	287	6.59	(1.66)	5.49	(2.43)	5.46	(2.36)
mourn	1870	1.80	(1.73)	5.87	(2.6)	2.87	(1.91)
mouth	1871	5.46	(1.53)	4.79	(2.48)	5.93	(1.86)
movie	288	6.86	(1.81)	4.93	(2.54)	5.00	(1.79)
mucus	886	3.34	(2.29)	3.41	(2.17)	4.80	(1.83)
mud	1872	4.81	(1.88)	3.81	(1.96)	5.30	(1.77)
muddy	887	4.44	(2.07)	4.13	(2.13)	4.73	(1.77)
muffin	888	6.57	(2.04)	4.76	(2.42)	5.51	(1.63)
muffle	1873	4.47	(1.43)	4.90	(1.84)	4.53	(1.78)
mug	1874	5.42	(1.84)	4.61	(2.19)	5.13	(1.93)
mule	1875	5.15	(1.57)	4.27	(1.78)	5.08	(1.72)
murderer	289	1.53	(0.96)	7.47	(2.18)	3.77	(3.06)
muscular	290	6.82	(1.63)	5.47	(2.2)	6.58	(2.28)
museum	889	5.54	(1.86)	3.60	(2.13)	5.32	(1.68)
mushroom	567	5.78	(2.22)	4.72	(2.33)	5.52	(2.1)
music	291	8.13	(1.09)	5.32	(3.19)	6.39	(2.44)
musician	1876	7.21	(1.52)	6.10	(2.16)	5.93	(1.79)
mutation	890	3.91	(2.44)	4.84	(2.52)	4.07	(2.1)
mutilate	292	1.82	(1.45)	6.41	(2.94)	3.41	(2.71)
mutilation	1877	1.97	(1.27)	6.07	(2.5)	2.97	(2.01)
mystic	891	6.00	(2.21)	4.84	(2.57)	5.52	(1.93)
myth	1878	5.86	(2.14)	5.50	(2.67)	4.82	(1.36)
nab	1879	4.16	(1.57)	5.32	(1.44)	5.20	(1.8)
nag	1880	2.90	(1.64)	5.40	(2.16)	4.27	(1.98)
nail	1881	5.14	(1.79)	4.57	(2.01)	5.25	(1.29)
naked	892	6.34	(2.42)	5.80	(2.8)	6.00	(2.05)

Description	Word No.	Valence Mean(SD)		Arousal Mean(SD)		Dominance Mean (SD)	
name	893	5.55	(2.24)	4.25	(2.47)	5.16	(2.08)
napkin	1882	4.84	(1.1)	4.00	(2.12)	5.13	(1.56)
narcotic	894	4.29	(2.3)	4.93	(2.57)	4.44	(2.43)
nasty	895	3.58	(2.38)	4.89	(2.5)	5.00	(2.17)
nation	1883	6.17	(1.91)	4.60	(2.24)	5.27	(2.33)
natural	896	6.59	(1.57)	4.09	(2.37)	5.57	(1.69)
nature	293	7.65	(1.37)	4.37	(2.51)	4.95	(2.72)
necklace	1884	6.39	(1.69)	4.87	(2.09)	5.50	(1.8)
nectar	294	6.90	(1.53)	3.89	(2.48)	4.54	(2.06)
needle	897	3.82	(1.73)	5.36	(2.89)	3.95	(2.17)
neglect	898	2.63	(1.64)	4.83	(2.31)	3.85	(2.29)
nerves	1885	4.28	(1.75)	6.31	(2.18)	4.72	(2.05)
nervous	899	3.29	(1.47)	6.59	(2.07)	3.56	(1.73)
nestle	1886	6.63	(1.59)	4.10	(1.99)	5.97	(1.73)
net	1887	4.80	(1.65)	4.83	(2.02)	4.93	(1.87)
neurotic	900	4.45	(2.23)	5.13	(2.76)	4.41	(2.05)
news	901	5.30	(1.67)	5.17	(2.11)	4.60	(1.88)
newspaper	1888	5.52	(1.29)	4.42	(1.86)	5.26	(1.37)
nice	902	6.55	(2.44)	4.38	(2.69)	5.58	(2.2)
niece	1889	6.33	(1.71)	4.60	(2.08)	5.40	(1.59)
night	1890	6.06	(1.95)	5.94	(2.41)	4.74	(2.11)
nightmare	295	1.91	(1.54)	7.59	(2.23)	3.68	(2.76)
nine	1891	5.07	(0.66)	4.04	(2.12)	5.18	(1.36)
nip	1892	4.59	(1.6)	5.04	(1.81)	5.04	(1.19)
nipple	903	6.27	(1.81)	5.56	(2.55)	5.57	(2)
noise	1893	4.93	(1.9)	4.86	(2.3)	4.61	(1.95)
noisy	904	5.02	(2.02)	6.38	(1.78)	4.93	(1.76)
nonchalant	296	4.74	(1.11)	3.12	(1.93)	4.31	(1.54)
nonsense	905	4.61	(1.63)	4.17	(2.02)	4.90	(1.55)
noon	1894	6.30	(1.54)	3.65	(1.96)	5.08	(1.67)
noose	906	3.76	(1.64)	4.39	(2.08)	4.17	(1.92)
nose	1895	4.71	(1.51)	4.32	(1.91)	4.93	(1.63)
nosey	1896	3.97	(1.72)	4.48	(1.95)	4.63	(1.9)
nourish	907	6.46	(1.69)	4.29	(2.51)	5.80	(1.62)
novel	1897	6.07	(1.62)	4.61	(2.3)	5.82	(1.7)

Description	Word No.	Valence Mean(SD)		Arousal Mean(SD)		Dominance Mean (SD)	
novice	1898	4.28	(1.58)	3.69	(1.95)	4.62	(1.95)
nozzle	1899	4.83	(1.42)	4.60	(1.57)	5.00	(1.7)
nuclear	1900	3.25	(2.2)	5.86	(2.56)	3.41	(2.14)
nude	520	6.82	(1.63)	6.41	(2.09)	5.96	(2.29)
nudist	1901	5.89	(1.86)	6.18	(2.09)	5.39	(1.88)
nuisance	908	3.27	(1.86)	4.49	(2.69)	4.36	(1.73)
number	1902	5.55	(1.15)	4.34	(2.22)	5.24	(2.01)
nun	909	4.93	(1.89)	2.93	(1.8)	4.93	(1.69)
nurse	538	6.08	(2.08)	4.84	(2.04)	4.84	(2.2)
nursery	910	5.73	(2.3)	4.04	(2.74)	5.18	(2.23)
oasis	1903	7.79	(1.42)	5.04	(2.87)	6.11	(1.91)
oath	1904	5.57	(1.52)	4.80	(1.69)	5.07	(2.1)
obesity	911	2.73	(1.85)	3.87	(2.82)	3.74	(2.45)
obey	912	4.52	(1.88)	4.23	(1.72)	4.26	(2.4)
object	1905	5.20	(1.04)	4.04	(2.18)	5.29	(1.57)
obnoxious	913	3.50	(2.18)	4.74	(2.42)	5.39	(2.2)
obscene	914	4.23	(2.3)	5.04	(2.3)	4.48	(1.91)
obsessed	1906	3.60	(1.96)	6.24	(1.86)	4.24	(2.23)
obsession	915	4.52	(2.13)	6.41	(2.13)	4.77	(2.38)
obstinate	1907	4.60	(1.59)	3.97	(1.75)	4.86	(1.25)
ocean	297	7.12	(1.72)	4.95	(2.79)	5.53	(2.75)
octopus	1908	6.03	(1.59)	5.20	(1.85)	4.87	(2.01)
odd	916	4.82	(2.04)	4.27	(2.46)	4.77	(1.89)
odor	1909	2.52	(1.57)	5.13	(2.1)	4.23	(1.96)
offend	917	2.76	(1.5)	5.56	(2.06)	3.73	(2.03)
office	568	5.24	(1.59)	4.08	(1.92)	5.59	(1.89)
officer	1910	3.72	(1.89)	5.55	(2.26)	4.07	(2.7)
oil	1911	4.60	(1.48)	4.72	(2.28)	4.72	(2.02)
old	1912	3.31	(1.89)	3.50	(1.95)	3.29	(1.8)
omelet	1913	6.48	(1.85)	4.52	(1.9)	5.40	(1.5)
omit	1914	4.19	(1.47)	4.00	(1.98)	4.73	(2.29)
onion	1915	4.43	(1.91)	4.60	(2.27)	4.97	(1.16)
opera	1916	4.84	(2.28)	3.97	(2.43)	5.07	(1.87)
opinion	298	6.28	(1.45)	4.89	(2.46)	5.53	(1.93)
opportunity	1917	7.41	(1.52)	6.47	(1.83)	6.59	(1.68)

Description	Word No.	Valence Mean(SD)		Arousal Mean(SD)		Dominance Mean (SD)	
oppose	1918	4.14	(1.43)	5.29	(1.82)	5.14	(2.29)
optimism	918	6.95	(2.24)	5.34	(2.58)	6.61	(2.06)
optimistic	1919	7.59	(1.32)	5.83	(2.24)	7.00	(1.51)
option	919	6.49	(1.31)	4.74	(2.23)	6.34	(1.8)
orange	1920	6.47	(1.66)	4.97	(2.28)	5.31	(2.04)
orangutan	1921	6.59	(1.68)	4.64	(2.23)	4.68	(1.44)
orchestra	299	6.02	(1.89)	3.52	(2.29)	5.17	(2.14)
ordeal	1922	3.77	(1.31)	4.92	(1.75)	4.42	(1.42)
organ	1923	5.24	(1.57)	4.54	(1.86)	4.96	(1.75)
orgasm	920	8.32	(1.31)	8.10	(1.45)	6.83	(2.18)
orphan	1924	2.29	(1.6)	5.35	(1.82)	3.87	(1.86)
ounce	1925	5.50	(1.07)	4.41	(1.94)	4.97	(1.45)
outdoors	521	7.47	(1.8)	5.92	(2.55)	6.27	(2.24)
outlet	1926	5.52	(1.53)	3.97	(2.16)	5.55	(1.92)
outrage	921	3.52	(2.12)	6.83	(2.26)	5.26	(2.72)
outstanding	922	7.75	(1.75)	6.24	(2.59)	6.40	(2.29)
oven	1927	5.71	(1.33)	3.78	(1.76)	5.41	(1.67)
overcast	923	3.65	(1.61)	3.46	(1.92)	4.20	(1.79)
overweight	1928	2.00	(1.21)	5.52	(2.44)	4.19	(2.77)
overwhelmed	300	4.19	(2.61)	7.00	(2.37)	3.89	(2.58)
owe	1929	3.25	(1.78)	5.68	(2.16)	3.07	(1.72)
owl	522	5.80	(1.31)	3.98	(1.87)	5.82	(1.62)
pacify	1930	6.10	(1.73)	4.23	(2.1)	5.33	(2.01)
packet	1931	4.69	(1.23)	4.27	(2.25)	4.73	(1.66)
pact	1932	6.20	(1.3)	5.20	(2.06)	6.30	(1.39)
pail	1933	5.04	(1.23)	3.64	(1.87)	5.43	(1.75)
pain	301	2.13	(1.81)	6.50	(2.49)	3.71	(2.53)
paint	924	5.62	(1.72)	4.10	(2.36)	5.75	(1.71)
painting	1934	6.59	(1.65)	5.00	(2.04)	5.81	(1.49)
palace	302	7.19	(1.78)	5.10	(2.75)	5.69	(2.17)
pale	1935	3.17	(1.47)	3.50	(1.71)	4.32	(1.22)
pamphlet	925	4.79	(1.05)	3.62	(2.02)	4.63	(1.48)
pan	1936	5.46	(1.23)	4.54	(1.9)	5.54	(1.45)
pancakes	523	6.08	(1.83)	4.06	(2.13)	5.76	(1.61)
pang	1937	4.27	(1.8)	5.90	(2.14)	4.48	(1.4)

Description	Word No.	Valence Mean(SD)		Arousal Mean(SD)		Dominance Mean (SD)	
panic	601	3.12	(1.84)	7.02	(2.02)	3.20	(1.67)
pant	1938	4.61	(1.64)	5.14	(2.16)	4.86	(2.01)
pantry	1939	6.58	(1.69)	4.57	(2.1)	5.63	(2.16)
pantyhose	1940	5.86	(1.78)	5.07	(1.98)	5.29	(1.86)
paper	303	5.20	(1.21)	2.50	(1.85)	4.47	(1.67)
paradise	304	8.72	(0.6)	5.12	(3.38)	6.03	(2.79)
paralysis	926	1.98	(1.44)	4.73	(2.83)	2.56	(1.82)
parch	1941	3.27	(1.78)	4.72	(1.94)	4.25	(1.96)
parlor	1942	5.40	(1.28)	3.83	(1.74)	5.47	(1.11)
parrot	1943	6.94	(1.61)	5.37	(2.13)	4.53	(2.03)
part	927	5.11	(1.78)	3.82	(2.24)	4.75	(1.59)
party	305	7.86	(1.83)	6.69	(2.84)	5.83	(2.46)
passage	928	5.28	(1.44)	4.36	(2.13)	5.02	(1.62)
passion	306	8.03	(1.27)	7.26	(2.57)	6.13	(2.24)
passive	1944	4.17	(1.97)	3.69	(2.16)	4.17	(2.27)
pasta	524	6.69	(1.64)	4.94	(2.04)	5.80	(1.47)
pastry	1945	7.18	(1.83)	5.79	(2.32)	5.64	(2.04)
patent	307	5.29	(1.08)	3.50	(1.84)	4.90	(1.79)
path	1946	5.74	(1.32)	4.84	(2.21)	5.97	(1.68)
patient	929	5.29	(1.89)	4.21	(2.37)	4.90	(2.31)
patio	1947	5.83	(1.42)	3.75	(2.07)	5.21	(1.83)
patriot	930	6.71	(1.69)	5.17	(2.53)	5.90	(1.54)
patriotic	1948	6.62	(2)	5.54	(2.23)	6.35	(2)
patriotism	1949	6.85	(1.94)	5.56	(2.76)	6.52	(2.23)
pea	1950	5.26	(0.93)	3.45	(2.05)	5.39	(1.61)
peace	308	7.72	(1.75)	2.95	(2.55)	5.45	(2.84)
peaceful	1951	7.77	(1.28)	2.87	(2.39)	5.70	(1.93)
peanut	1952	5.56	(2.1)	3.74	(2.18)	5.11	(1.8)
pear	1953	6.52	(1.65)	3.96	(2.38)	5.37	(1.92)
pecan	1954	5.68	(1.72)	4.04	(1.93)	5.07	(1.49)
pee	1955	5.41	(2.06)	4.84	(2.22)	6.06	(1.93)
peep	1956	4.64	(1.95)	4.71	(1.9)	4.79	(1.85)
penalty	931	2.83	(1.56)	5.10	(2.31)	3.95	(1.97)
pencil	309	5.22	(0.68)	3.14	(1.9)	4.78	(1.73)
penis	932	5.90	(1.72)	5.54	(2.63)	5.92	(2.54)

Description	Word No.	Valence Mean(SD)		Arousal Mean(SD)		Dominance Mean (SD)	
penny	1957	5.06	(1.7)	3.52	(1.88)	5.06	(1.53)
penthouse	933	6.81	(1.64)	5.52	(2.49)	6.52	(1.82)
people	525	7.33	(1.7)	5.94	(2.09)	6.14	(2.02)
perfection	310	7.25	(2.05)	5.95	(2.73)	6.71	(2.26)
perfume	934	6.76	(1.48)	5.05	(2.36)	5.93	(1.69)
period	1958	3.80	(1.65)	4.30	(1.86)	3.80	(1.86)
perish	1959	2.90	(1.99)	5.30	(2.49)	3.20	(1.92)
permit	1960	5.47	(2.2)	5.08	(1.92)	5.17	(2.38)
person	311	6.32	(1.74)	4.19	(2.45)	5.35	(2.02)
personality	1961	7.48	(1.5)	5.88	(2.29)	5.50	(2.34)
pervert	312	2.79	(2.12)	6.26	(2.61)	4.72	(2.83)
pessimism	1962	3.10	(2.15)	4.74	(2.14)	4.48	(2)
pest	313	3.13	(1.82)	5.62	(2.15)	5.29	(2.13)
pester	1963	2.77	(1.45)	5.77	(2.13)	4.60	(1.65)
pet	935	6.79	(2.32)	5.10	(2.59)	5.85	(2.28)
phase	936	5.17	(0.79)	3.98	(1.82)	4.65	(1.72)
photo	1964	6.77	(1.5)	5.63	(1.81)	5.70	(1.21)
picket	1965	4.97	(1.02)	4.45	(1.84)	5.55	(1.68)
picnic	1966	7.07	(1.25)	5.18	(2.16)	5.93	(1.56)
pie	314	6.41	(1.89)	4.20	(2.4)	5.35	(1.78)
pier	1967	6.61	(1.56)	5.32	(2.48)	5.71	(1.51)
pierce	1968	4.35	(1.84)	5.81	(2.18)	4.97	(2.01)
pig	937	5.07	(1.97)	4.20	(2.42)	5.34	(1.88)
pigeon	1969	4.93	(2.06)	4.92	(2.08)	5.68	(2.04)
pill	1970	4.81	(1.28)	4.63	(1.71)	5.13	(1.66)
pillow	315	7.92	(1.4)	2.97	(2.52)	4.56	(2.17)
pilot	1971	6.11	(1.62)	5.07	(2.43)	5.39	(2.5)
pin	1972	4.50	(1.46)	5.17	(1.82)	4.77	(1.72)
pinch	938	3.83	(1.7)	4.59	(2.1)	4.76	(1.73)
pint	1973	5.41	(1.74)	5.04	(1.73)	5.18	(1.49)
pipe	1974	5.52	(1.74)	4.69	(2)	5.66	(1.67)
pistol	939	4.20	(2.58)	6.15	(2.19)	5.05	(2.77)
pity	940	3.37	(1.57)	3.72	(2.02)	4.12	(1.82)
pizza	526	6.65	(2.23)	5.24	(2.09)	5.69	(1.9)
plain	941	4.39	(1.46)	3.52	(2.05)	4.71	(1.68)

Description	Word No.	Valence Mean(SD)		Arousal Mean(SD)		Dominance Mean (SD)	
plan	1975	6.47	(1.36)	4.77	(2.49)	6.55	(1.53)
plane	539	6.43	(1.98)	6.14	(2.39)	4.78	(2.19)
planet	1976	5.69	(1.6)	4.34	(2.16)	4.47	(1.54)
plant	316	5.98	(1.83)	3.62	(2.25)	4.71	(2.12)
plate	1977	5.30	(1.68)	4.00	(1.89)	5.55	(1.96)
play	1978	8.10	(1.16)	6.93	(1.8)	6.97	(1.5)
plaza	1979	5.59	(1.32)	3.89	(1.93)	5.21	(1.13)
plea	1980	4.04	(1.57)	5.11	(2.01)	3.96	(2.47)
plead	1981	3.74	(1.55)	5.00	(2.13)	4.06	(2.08)
pleased	1982	7.79	(1.75)	5.68	(2.63)	6.29	(2.03)
pleasure	317	8.28	(0.92)	5.74	(2.81)	6.15	(2.31)
pledge	1983	5.62	(1.65)	4.92	(2.1)	4.50	(2.2)
plod	1984	4.74	(1.09)	3.80	(1.81)	4.77	(1.5)
poach	1985	3.76	(1.41)	5.24	(1.84)	4.59	(1.72)
poem	1986	6.48	(1.94)	4.59	(2.37)	5.34	(1.65)
poet	1987	6.46	(1.55)	4.67	(2.37)	4.81	(1.71)
poetry	318	5.86	(1.91)	4.00	(2.85)	5.31	(1.81)
poise	1988	6.90	(1.6)	5.33	(2.04)	6.63	(1.63)
poison	319	1.98	(1.44)	6.05	(2.82)	3.10	(2.44)
poker	1989	6.31	(1.85)	5.64	(2.13)	5.46	(1.29)
pole	1990	5.13	(0.72)	4.35	(1.33)	5.32	(1.22)
police	1991	4.17	(2.15)	6.27	(2.13)	3.30	(1.97)
policy	1992	4.50	(1.55)	4.30	(2)	4.03	(1.81)
politeness	320	7.18	(1.5)	3.74	(2.37)	5.74	(1.7)
politics	1993	3.97	(1.88)	5.48	(2.1)	4.00	(2.15)
pollen	1994	3.31	(1.57)	4.69	(2.22)	3.91	(1.65)
pollute	321	1.85	(1.11)	6.08	(2.42)	4.92	(2.51)
pollution	1995	2.43	(1.79)	5.77	(2.37)	3.66	(2.36)
pond	1996	6.14	(1.51)	4.10	(2.14)	5.38	(1.59)
pony	1997	6.00	(1.74)	5.07	(2)	5.29	(1.49)
pool	1998	7.70	(1.37)	5.90	(2.52)	5.93	(2.1)
poor	1999	2.28	(1.73)	5.21	(2.86)	2.68	(2.39)
popcorn	2000	6.93	(1.61)	4.89	(2.06)	5.42	(1.33)
porch	2001	6.14	(1.53)	3.82	(2.21)	5.39	(1.29)
pork	2002	6.20	(1.73)	4.77	(2.47)	5.30	(1.78)

Description	Word No.	Valence Mean(SD)		Arousal Mean(SD)		Dominance Mean (SD)	
porpoise	2003	5.93	(1.34)	4.37	(1.88)	5.07	(1.41)
position	2004	5.74	(1.18)	5.10	(2.29)	6.13	(1.61)
positive	2005	8.07	(1.1)	5.57	(2.18)	6.19	(1.83)
poster	942	5.34	(1.75)	3.93	(2.56)	4.91	(1.87)
potential	2006	7.00	(1.83)	5.93	(2.39)	6.50	(2.29)
pout	2007	2.83	(1.86)	5.07	(1.75)	4.10	(2.06)
poverty	322	1.67	(0.9)	4.87	(2.66)	3.21	(2.21)
power	323	6.54	(2.21)	6.67	(1.87)	7.28	(2.35)
powerful	324	6.84	(1.8)	5.83	(2.69)	7.19	(2.52)
prairie	325	5.75	(1.43)	3.41	(2.17)	4.62	(2.13)
praise	2008	7.24	(1.88)	6.28	(2.07)	6.59	(2.29)
pray	2009	6.23	(2.01)	4.57	(2.25)	5.40	(2.28)
prayer	2010	6.24	(2.34)	5.14	(2.52)	5.64	(2.48)
preach	2011	5.41	(2.44)	5.89	(2.36)	4.31	(2.24)
prefer	2012	6.00	(1.86)	4.50	(1.78)	5.47	(1.55)
pregnant	2013	4.30	(2.69)	6.37	(2.86)	4.11	(2.74)
preoccupied	2014	4.03	(1.82)	4.93	(1.96)	4.31	(2.14)
present	943	6.95	(1.85)	5.12	(2.39)	5.83	(1.78)
president	2015	5.20	(2.17)	5.63	(2.14)	4.77	(2.6)
press	2016	4.81	(0.93)	4.61	(1.82)	4.58	(1.2)
pressure	944	3.38	(1.61)	6.07	(2.26)	3.45	(2.07)
prestige	945	7.26	(1.9)	5.86	(2.08)	6.90	(1.96)
pretty	326	7.75	(1.26)	6.03	(2.22)	5.50	(1.97)
price	2017	3.83	(1.26)	4.87	(1.91)	4.10	(2.01)
prick	946	3.98	(1.73)	4.70	(2.59)	4.47	(1.88)
pride	327	7.00	(2.11)	5.83	(2.48)	7.06	(2.15)
priest	328	6.42	(2)	4.41	(2.71)	4.88	(2.07)
prince	2018	7.03	(1.71)	6.07	(2.19)	5.59	(2.34)
princess	2019	7.08	(1.66)	5.46	(2.22)	6.32	(2.07)
prison	329	2.05	(1.34)	5.70	(2.56)	4.20	(2.58)
privacy	330	5.88	(1.5)	4.12	(1.83)	5.66	(1.78)
problem	2020	2.74	(1.61)	5.81	(2.22)	4.04	(2.53)
procedure	2021	4.55	(1.66)	4.68	(2.13)	4.71	(1.72)
profit	331	7.63	(1.3)	6.68	(1.78)	5.85	(2.47)
progress	947	7.73	(1.34)	6.02	(2.58)	6.76	(2.05)
promotion	332	8.20	(1.15)	6.44	(2.58)	6.79	(2.28)
propeller	2022	5.43	(1.55)	4.43	(2.7)	5.33	(1.24)
proper	2023	5.93	(2.02)	3.56	(1.6)	5.04	(2.19)
prose	2024	5.56	(1.28)	4.41	(1.8)	5.26	(1.35)
prostitute	2025	4.24	(2.49)	5.60	(2.27)	5.64	(1.93)
protected	333	7.29	(1.79)	4.09	(2.77)	5.80	(2.54)
proud	334	8.03	(1.56)	5.56	(3.01)	6.74	(2.73)
pry	2026	3.47	(1.48)	5.03	(2.3)	4.33	(1.99)
pub	2027	6.35	(1.92)	5.48	(2.2)	6.19	(1.66)
puddle	2028	5.07	(1.65)	4.14	(1.82)	4.68	(1.87)
pulp	2029	5.00	(2.27)	4.03	(1.9)	5.00	(1.46)
pulpit	2030	5.45	(1.45)	4.11	(1.87)	5.11	(0.92)
pun	2031	6.20	(1.67)	4.60	(1.98)	5.27	(1.41)
pungent	948	3.95	(2.09)	4.24	(2.17)	4.78	(1.52)
punish	2032	1.92	(1.06)	6.69	(2.22)	2.65	(2.08)
punishment	335	2.22	(1.41)	5.93	(2.4)	3.50	(2.43)
punk	2033	4.37	(2.2)	4.59	(2.31)	5.23	(2.45)
puppy	336	7.56	(1.9)	5.85	(2.78)	5.51	(2.39)
purify	2034	6.63	(1.63)	4.90	(2.11)	5.63	(1)
pus	602	2.86	(1.91)	4.82	(2.06)	4.35	(1.82)
pushy	2035	3.30	(1.7)	6.00	(1.76)	3.73	(2.1)
putrid	337	2.38	(1.71)	5.74	(2.26)	4.89	(2.09)
python	949	4.05	(2.48)	6.18	(2.25)	4.52	(2.56)
quality	950	6.25	(1.59)	4.48	(2.12)	5.64	(1.59)
quarrel	338	2.93	(2.06)	6.29	(2.56)	4.02	(2.16)
quart	951	5.39	(2.01)	3.59	(2.51)	5.20	(1.86)
queen	952	6.44	(1.43)	4.76	(2.18)	5.49	(2.12)
quench	2036	5.52	(1.86)	3.93	(1.65)	5.71	(1.76)
quick	953	6.64	(1.61)	6.57	(1.78)	6.57	(1.91)
quiet	339	5.58	(1.83)	2.82	(2.13)	4.42	(2.3)
quilt	2037	6.38	(1.76)	4.31	(1.85)	5.48	(1.53)
quit	2038	2.46	(1.4)	4.82	(2.52)	3.64	(2.45)
rabbit	527	6.57	(1.92)	4.02	(2.19)	6.08	(1.72)
rabies	340	1.77	(0.97)	6.10	(2.62)	3.85	(2.34)
race	2039	6.07	(2)	5.43	(2.47)	5.17	(1.98)

Description	Word No.	Valence Mean(SD)		Arousal Mean(SD)		Dominance Mean (SD)	
rack	2040	6.19	(1.78)	5.15	(2.92)	5.37	(1.82)
radar	2041	4.54	(1.79)	5.57	(2.01)	4.64	(1.37)
radiant	954	6.73	(2.17)	5.39	(2.82)	5.61	(2.17)
radiator	955	4.67	(1.05)	4.02	(1.94)	4.81	(1.38)
radio	341	6.73	(1.47)	4.78	(2.82)	5.28	(1.85)
raft	2042	5.72	(1.13)	5.04	(2.08)	4.89	(1.23)
rag	2043	3.70	(1.51)	3.70	(2.11)	4.22	(2.17)
rage	342	2.41	(1.86)	8.17	(1.4)	5.68	(3.01)
rain	569	5.08	(2.51)	3.65	(2.35)	4.78	(1.68)
rainbow	343	8.14	(1.23)	4.64	(2.88)	4.72	(2.37)
raise	2044	6.67	(1.99)	5.63	(2.37)	5.77	(1.76)
rally	2045	6.23	(1.79)	6.50	(2.06)	5.57	(1.81)
ramble	2046	4.23	(1.87)	4.53	(2.36)	4.47	(1.74)
rancid	956	4.34	(2.28)	5.04	(2.27)	4.59	(1.86)
randy	2047	5.77	(1.28)	4.71	(2.16)	5.13	(1.94)
rape	344	1.25	(0.91)	6.81	(3.17)	2.97	(2.94)
rash	2048	2.54	(1.79)	5.43	(2.28)	3.68	(2.18)
rat	345	3.02	(1.66)	4.95	(2.36)	4.55	(2.14)
rattle	346	5.03	(1.23)	4.36	(2.18)	4.17	(1.56)
raw	2049	4.32	(1.68)	5.04	(2.16)	5.22	(1.5)
razor	957	4.81	(2.16)	5.36	(2.44)	4.91	(1.95)
react	2050	5.63	(1)	5.87	(1.74)	5.53	(1.74)
ready	2051	6.23	(1.26)	5.23	(2)	6.55	(1.79)
realize	2052	6.25	(1.43)	5.26	(1.97)	5.30	(1.98)
reap	2053	5.50	(2.01)	4.89	(1.91)	5.43	(1.93)
rebuff	2054	5.00	(1.97)	4.64	(2.37)	4.86	(1.69)
recede	2055	4.19	(1.02)	4.08	(1.76)	4.62	(1.17)
recipe	2056	6.70	(1.53)	5.60	(1.87)	6.10	(1.4)
red	570	6.41	(1.61)	5.29	(2.04)	5.78	(1.59)
redeem	2057	7.47	(1.28)	5.43	(2.11)	6.20	(1.52)
reef	2058	6.97	(1.72)	5.52	(2.38)	4.97	(1.76)
refine	2059	5.50	(1.41)	4.48	(1.7)	5.71	(1.78)
reflection	2060	5.97	(1.83)	4.38	(2.01)	6.10	(1.7)
refreshment	347	7.44	(1.29)	4.45	(2.7)	5.00	(1.92)
refrigerator	2061	6.14	(1.27)	4.66	(1.78)	6.28	(1.79)
refugee	2062	3.65	(1.44)	5.04	(2.01)	3.69	(2.43)
refute	2063	4.57	(1.65)	5.07	(2.05)	4.90	(1.54)
regime	2064	4.88	(1.45)	4.88	(1.96)	4.97	(2.19)
regret	2065	2.26	(1.44)	5.67	(2.17)	3.23	(1.65)
regretful	348	2.28	(1.42)	5.74	(2.32)	3.43	(2.52)
reign	2066	5.82	(1.74)	4.93	(2.34)	6.57	(2.28)
rejected	349	1.50	(1.09)	6.37	(2.56)	2.72	(2.58)
rejection	2067	1.77	(1.22)	6.00	(2.48)	2.17	(1.49)
relate	2068	6.04	(1.7)	4.33	(2.5)	5.41	(1.58)
relax	2069	7.87	(1.7)	2.47	(2)	6.37	(2.75)
relaxed	350	7.00	(1.77)	2.39	(2.13)	5.55	(1.9)
relent	2070	4.30	(1.27)	4.74	(1.83)	4.15	(1.88)
relic	2071	5.76	(1.23)	5.16	(1.82)	5.84	(1.49)
relief	2072	6.81	(1.78)	4.17	(1.97)	5.60	(1.79)
religion	2073	5.07	(2.21)	5.85	(1.79)	5.30	(2.43)
rely	2074	5.27	(1.17)	4.66	(2.02)	4.38	(2.29)
remorse	2075	2.48	(1.55)	5.66	(2.26)	3.31	(2.19)
remove	2076	4.13	(1.59)	4.73	(2)	4.70	(1.76)
render	2077	4.50	(1.33)	4.65	(1.83)	4.85	(1.67)
rent	2078	4.16	(1.21)	5.04	(1.1)	4.76	(1.88)
repent	2079	4.93	(2.39)	5.11	(2.67)	4.21	(2.59)
repentant	351	5.53	(1.86)	4.69	(1.98)	5.42	(2.06)
report	2080	4.11	(1.75)	4.18	(2.61)	4.29	(2.12)
reptile	958	4.77	(2)	5.18	(2.19)	4.77	(2.02)
repulsed	2081	2.48	(1.6)	5.93	(2.07)	4.10	(1.72)
rescue	352	7.70	(1.24)	6.53	(2.56)	6.45	(2.29)
resent	959	3.76	(1.9)	4.47	(2.12)	4.46	(2.09)
reserved	353	4.88	(1.83)	3.27	(2.05)	4.30	(1.93)
resign	2082	3.47	(1.63)	4.21	(2.1)	4.57	(1.73)
resist	2083	5.12	(1.76)	5.35	(1.78)	5.80	(1.65)
respect	354	7.64	(1.29)	5.19	(2.39)	6.89	(2.11)
respectful	355	7.22	(1.27)	4.60	(2.67)	5.67	(2.38)
response	2084	5.67	(1.14)	5.00	(1.94)	4.96	(1.83)
restaurant	960	6.76	(1.85)	5.41	(2.55)	5.73	(1.41)
retain	2085	5.38	(1.66)	4.57	(2.25)	5.79	(1.93)

Description	Word No.	Valence Mean(SD)		Arousal Mean(SD)		Dominance Mean (SD)	
retard	2086	2.89	(2.08)	4.96	(2.19)	3.43	(1.91)
retire	2087	6.37	(2.51)	4.70	(2.63)	6.22	(2.44)
retreat	2088	4.50	(1.68)	4.43	(2.05)	4.60	(2.18)
reunion	961	6.48	(2.45)	6.34	(2.35)	5.64	(1.95)
reveal	2089	6.29	(1.98)	6.32	(2.04)	5.21	(1.91)
revere	2090	5.45	(2.05)	4.29	(2.09)	5.04	(1.79)
reverent	356	5.35	(1.21)	4.00	(1.6)	4.67	(1.68)
revert	2091	4.53	(1.61)	4.73	(1.74)	4.23	(1.98)
revise	2092	4.33	(1.75)	4.67	(1.79)	4.93	(1.83)
revive	2093	6.68	(1.95)	5.54	(2.43)	5.58	(2.15)
revolt	357	4.13	(1.78)	6.56	(2.34)	6.18	(2.11)
revolver	962	4.02	(2.44)	5.55	(2.39)	4.39	(2.47)
reward	358	7.53	(1.67)	4.95	(2.62)	6.00	(2.14)
rhythm	2094	7.06	(1.63)	5.73	(2.07)	6.23	(1.76)
rich	2095	7.17	(2.2)	5.80	(2.59)	6.57	(1.79)
riches	359	7.70	(1.95)	6.17	(2.7)	6.74	(2.43)
riddle	2096	5.93	(1.34)	5.20	(1.77)	4.90	(1.65)
ridicule	360	3.13	(2.24)	5.83	(2.73)	3.87	(2.7)
rifle	603	4.02	(2.76)	6.35	(2.04)	4.16	(2.71)
right	2097	6.45	(1.55)	4.79	(2.18)	6.38	(1.78)
rigid	963	3.66	(2.12)	4.66	(2.47)	4.61	(2.04)
rigor	2098	4.96	(1.82)	5.57	(2.5)	5.18	(2.13)
ring	2099	6.33	(1.58)	5.37	(2.17)	4.73	(1.74)
rink	2100	5.70	(1.66)	4.33	(2.06)	5.73	(1.76)
riot	361	2.96	(1.93)	6.39	(2.63)	4.18	(2.47)
risk	2101	4.64	(2.04)	6.54	(2.12)	3.82	(2.23)
river	362	6.85	(1.69)	4.51	(2.42)	5.10	(1.86)
roach	363	2.35	(1.7)	6.64	(2.64)	4.82	(2.94)
road	2102	5.53	(1.28)	4.90	(1.65)	5.33	(1.88)
roast	2103	5.80	(1.71)	5.10	(2.14)	5.93	(1.39)
rob	2104	2.80	(1.67)	6.40	(2.59)	2.80	(1.81)
robber	964	2.61	(1.69)	5.62	(2.72)	3.62	(2.38)
robbery	2105	2.42	(1.52)	6.90	(2.01)	2.26	(1.55)
robot	2106	5.36	(1.31)	4.71	(2.14)	4.46	(1.97)
rock	965	5.56	(1.38)	4.52	(2.37)	5.15	(2.01)

Description	Word No.	Valence Mean(SD)		Arousal Mean(SD)		Dominance Mean (SD)	
rodeo	2107	5.62	(1.74)	5.00	(2.19)	4.69	(1.49)
role	2108	6.11	(1.31)	4.61	(2.17)	5.68	(1.85)
rollercoaster	528	8.02	(1.63)	8.06	(1.71)	5.10	(2.76)
romance	2109	7.61	(1.58)	6.90	(2.22)	6.03	(1.96)
romantic	364	8.32	(1)	7.59	(2.07)	6.08	(2.29)
roof	2110	5.40	(1.5)	4.07	(2.09)	5.27	(2)
room	2111	5.52	(1.12)	3.77	(1.96)	5.52	(1.61)
rope	2112	5.07	(1.41)	4.57	(1.65)	5.00	(1.74)
rot	2113	2.68	(1.87)	5.18	(2.74)	3.07	(1.92)
rotten	365	2.26	(1.37)	4.53	(2.38)	4.32	(2.09)
rough	966	4.74	(2)	5.33	(2.04)	4.81	(1.7)
rower	2114	5.31	(1.31)	4.97	(1.77)	5.47	(0.95)
royalty	2115	6.57	(1.79)	5.59	(2.04)	5.76	(2.06)
ruby	2116	6.89	(1.34)	5.18	(2.34)	6.07	(1.68)
rude	366	2.50	(2.11)	6.31	(2.47)	4.91	(2.49)
rug	2117	4.93	(1.23)	3.63	(1.79)	5.57	(1.43)
rugby	2118	5.77	(1.33)	5.00	(2.02)	5.03	(1.4)
ruins	2119	4.84	(2.21)	5.48	(1.86)	4.61	(1.23)
ruler	2120	5.10	(1.7)	4.33	(2.51)	5.78	(2.38)
rules	2121	4.23	(1.41)	4.38	(1.52)	3.34	(1.86)
rum	2122	6.00	(2.19)	5.39	(2.5)	4.75	(2.52)
runner	571	5.67	(1.91)	4.76	(2.4)	5.47	(1.84)
rush	2123	3.97	(2.01)	6.38	(2.24)	3.88	(1.54)
rusty	367	3.86	(1.47)	3.77	(2.16)	4.53	(1.62)
rye	2124	5.24	(0.78)	3.96	(2.09)	5.28	(1.74)
sad	368	1.61	(0.95)	4.13	(2.38)	3.45	(2.18)
sadness	2125	2.21	(1.75)	5.21	(2.44)	2.82	(1.63)
safe	967	7.07	(1.9)	3.86	(2.72)	5.81	(2.06)
safety	2126	6.70	(1.9)	4.78	(2.55)	5.56	(2.31)
sailboat	529	7.25	(1.71)	4.88	(2.73)	5.86	(1.71)
saint	968	6.49	(1.7)	4.49	(1.9)	5.37	(2.11)
salad	369	5.74	(1.62)	3.81	(2.29)	5.47	(1.68)
salary	2127	6.75	(1.76)	5.85	(2.32)	5.30	(2.11)
sale	2128	7.07	(1.69)	6.00	(2.37)	6.33	(2.06)
salon	2129	5.69	(1.69)	4.92	(2.17)	5.35	(1.5)

Description	Word No.	Valence Mean(SD)		Arousal Mean(SD)		Dominance Mean (SD)	
salt	2130	5.56	(1.04)	4.54	(2.04)	5.75	(1.22)
salute	370	5.92	(1.57)	5.31	(2.23)	5.46	(2.05)
sand	2131	6.34	(2.04)	4.79	(2.35)	5.83	(1.85)
sandwich	2132	6.28	(1.56)	5.59	(2.21)	5.46	(1.9)
sapphire	371	7.00	(1.88)	5.00	(2.72)	5.55	(2.24)
satellite	2133	6.10	(1.5)	4.90	(2.08)	5.00	(1.87)
satire	2134	6.23	(1.91)	5.34	(2.04)	5.62	(1.54)
satisfaction	2135	7.69	(1.42)	6.83	(2.22)	6.76	(1.57)
satisfied	372	7.94	(1.19)	4.94	(2.63)	6.14	(2.37)
saucer	2136	5.09	(0.96)	4.41	(1.68)	5.06	(1.19)
save	969	6.45	(1.93)	4.95	(2.19)	6.00	(1.79)
savior	373	7.73	(1.56)	5.80	(3.01)	6.64	(2.18)
scalding	970	2.82	(2.12)	5.95	(2.55)	3.82	(2.3)
scandal	971	3.32	(1.81)	5.12	(2.22)	4.34	(1.73)
scapegoat	972	3.67	(1.65)	4.53	(2.13)	3.52	(1.7)
scar	973	3.38	(1.7)	4.79	(2.11)	3.88	(1.71)
scare	2137	3.62	(1.93)	6.56	(2.26)	3.66	(1.81)
scared	604	2.78	(1.99)	6.82	(2.03)	2.94	(2.19)
scarf	2138	5.64	(1.47)	3.46	(1.93)	5.46	(1.56)
scary	2139	3.24	(1.83)	6.69	(1.98)	3.66	(2.13)
scene	2140	5.59	(1.38)	4.93	(1.94)	5.14	(1.22)
scent	2141	6.30	(1.26)	5.30	(1.9)	5.27	(1.14)
scholar	374	7.26	(1.42)	5.12	(2.46)	6.59	(2.02)
school	2142	4.36	(2.13)	4.44	(2.22)	5.00	(2.15)
science	2143	4.76	(2.28)	4.71	(2.31)	4.82	(2.13)
scientific	2144	5.66	(2)	4.86	(1.94)	5.36	(1.93)
scissors	974	5.05	(0.96)	4.47	(1.76)	5.16	(1.84)
scoff	2145	3.94	(2.29)	5.25	(1.95)	4.78	(1.9)
scold	2146	2.81	(1.49)	6.06	(1.95)	3.39	(2.08)
scorch	2147	3.23	(1.99)	4.97	(2.49)	3.90	(1.6)
scorching	975	3.76	(1.83)	5.00	(2.74)	4.10	(2.01)
scorn	375	2.84	(2.07)	5.48	(2.52)	3.93	(2.64)
scornful	376	3.02	(2.03)	5.04	(2.56)	4.59	(2.18)
scorpion	976	3.69	(2.63)	5.38	(3.08)	3.98	(2.44)
scream	605	3.88	(2.07)	7.04	(1.96)	4.75	(2.21)

Description	Word No.	Valence Mean(SD)		Arousal Mean(SD)		Dominance Mean (SD)	
scrotum	2148	5.85	(1.38)	5.04	(2.39)	6.00	(1.57)
scuff	2149	3.93	(1.64)	5.10	(1.6)	4.43	(2.03)
scum	377	2.43	(1.56)	4.88	(2.36)	4.26	(1.99)
scurvy	378	3.19	(2)	4.71	(2.72)	4.48	(2.48)
sea	2150	7.00	(1.66)	4.83	(2.99)	5.28	(2.02)
seagull	2151	5.89	(1.91)	4.57	(2.33)	4.93	(1.62)
seal	2152	5.81	(1.44)	5.42	(1.79)	5.58	(1.5)
seasick	379	2.05	(1.2)	5.80	(2.88)	3.41	(2.39)
seat	380	4.95	(0.98)	2.95	(1.72)	4.84	(1.88)
secretary	2153	5.76	(1.3)	4.66	(2.04)	5.86	(1.62)
secure	381	7.57	(1.76)	3.14	(2.47)	5.93	(2.57)
security	2154	7.28	(1.51)	4.22	(2.45)	5.53	(2.27)
seduction	2155	7.53	(1.66)	7.43	(1.83)	5.63	(2.5)
seek	2156	5.59	(1.43)	5.12	(1.84)	5.28	(1.78)
seize	2157	3.52	(2.1)	6.52	(2.42)	3.26	(2.47)
self	2158	7.00	(1.58)	5.46	(2.47)	7.21	(1.96)
selfish	382	2.42	(1.62)	5.50	(2.62)	4.64	(2.31)
semen	2159	5.07	(1.72)	5.67	(1.67)	5.57	(1.72)
sensitive	2160	5.50	(1.87)	4.27	(2.21)	4.55	(1.55)
sentiment	977	5.98	(1.71)	4.41	(2.3)	5.09	(1.46)
sentry	2161	5.00	(1.17)	4.19	(1.74)	5.00	(1.5)
serene	2162	6.90	(1.99)	3.40	(2.43)	5.93	(1.74)
serious	383	5.08	(1.59)	4.00	(1.87)	5.12	(1.65)
sermon	2163	5.72	(2.03)	4.45	(2.47)	4.45	(2.34)
sever	2164	3.08	(1.68)	5.72	(2.19)	3.76	(1.98)
severe	978	3.20	(1.74)	5.26	(2.36)	3.83	(1.91)
sewage	2165	2.61	(2.16)	4.57	(2.4)	4.47	(1.48)
sex	384	8.05	(1.53)	7.36	(1.91)	5.75	(2.25)
sexy	530	8.02	(1.12)	7.36	(1.91)	6.82	(2.13)
shadow	385	4.35	(1.23)	4.30	(2.26)	4.19	(1.82)
shaky	2166	3.42	(1.17)	5.08	(1.83)	3.46	(1.45)
shame	2167	2.13	(1.14)	6.33	(2.04)	2.97	(1.73)
shamed	386	2.50	(1.34)	4.88	(2.27)	2.98	(1.94)
shark	606	3.65	(2.47)	7.16	(1.96)	2.63	(2.16)
shawl	2168	5.24	(1.64)	3.90	(2.14)	5.24	(1.77)

Description	Word No.	Valence Mean(SD)		Arousal Mean(SD)		Dominance Mean (SD)	
sheep	2169	6.44	(1.63)	3.78	(2.29)	5.41	(1.67)
shelf	2170	5.28	(0.77)	4.22	(1.52)	5.44	(1.32)
sheltered	387	5.75	(1.92)	4.28	(1.77)	3.76	(1.91)
shield	2171	6.54	(1.93)	5.36	(2.34)	6.43	(1.99)
shine	2172	7.07	(1.46)	5.25	(1.94)	6.29	(1.86)
ship	388	5.55	(1.4)	4.38	(2.29)	5.12	(2.31)
shock	2173	4.03	(2.25)	7.45	(1.97)	3.34	(2.19)
shoes	2174	6.19	(1.36)	4.52	(2.38)	5.89	(1.95)
shoplifter	2175	2.93	(1.85)	6.00	(2.27)	4.24	(2.34)
shopping	2176	6.48	(2.53)	5.79	(2.62)	6.50	(2.25)
short	2177	4.64	(1.79)	3.75	(2.15)	3.96	(2.24)
shotgun	979	4.37	(2.75)	6.27	(1.94)	5.29	(2.67)
shower	2178	7.04	(1.23)	5.54	(2.27)	6.32	(1.63)
shred	2179	4.46	(1.43)	5.00	(1.87)	4.89	(1.87)
shriek	980	3.93	(2.22)	5.36	(2.91)	4.30	(1.86)
shrimp	2180	6.00	(2.3)	4.52	(2.25)	6.45	(2.1)
shrink	2181	4.00	(1.2)	5.07	(2.05)	3.59	(2.11)
shrub	2182	4.92	(0.4)	4.04	(1.7)	5.52	(1.33)
shun	2183	2.97	(1.71)	5.24	(2.25)	3.66	(2)
shut	2184	4.53	(1.46)	4.87	(1.85)	4.40	(1.71)
shy	389	4.64	(1.83)	3.77	(2.29)	3.44	(1.96)
sick	607	1.90	(1.14)	4.29	(2.45)	3.04	(1.65)
sickness	390	2.25	(1.71)	5.61	(2.67)	3.84	(2.5)
sidle	2185	5.29	(1.01)	4.89	(1.4)	5.18	(0.61)
sigh	2186	4.15	(1.75)	2.78	(1.78)	4.44	(1.85)
sight	2187	6.58	(1.69)	4.73	(2.23)	5.47	(2.08)
silent	2188	4.79	(1.97)	3.46	(2.19)	4.96	(2.19)
silk	391	6.90	(1.27)	3.71	(2.51)	4.81	(1.93)
silly	981	7.41	(1.8)	5.88	(2.38)	6.00	(2.09)
silver	2189	6.85	(1.59)	5.19	(1.86)	5.65	(1.55)
simple	2190	6.50	(1.55)	3.73	(2.15)	6.10	(2.02)
sin	392	2.80	(1.67)	5.78	(2.21)	3.62	(2.29)
sinful	393	2.93	(2.15)	6.29	(2.43)	4.24	(2.73)
sing	2191	6.77	(2.24)	5.73	(2.1)	5.37	(2.34)
sissy	394	3.14	(1.96)	5.17	(2.57)	3.58	(2.74)

Description	Word No.	Valence Mean(SD)		Arousal Mean(SD)		Dominance Mean (SD)	
sister	2192	7.46	(1.77)	5.43	(2.66)	4.93	(2.14)
sizzle	2193	6.70	(1.42)	5.90	(1.94)	5.80	(1.54)
skate	2194	6.60	(1.48)	6.33	(1.71)	5.73	(1.86)
skeleton	2195	3.83	(1.97)	4.79	(2.06)	4.39	(2.08)
skeptical	395	4.52	(1.63)	4.91	(1.92)	4.50	(1.61)
ski	2196	6.80	(1.9)	6.62	(2.01)	5.21	(1.93)
skier	2197	5.72	(1.89)	5.36	(2.25)	5.75	(1.69)
skijump	531	7.06	(1.73)	7.06	(2.1)	4.90	(2.32)
skill	2198	6.90	(1.95)	5.70	(2.02)	6.33	(2.38)
skin	2199	5.55	(1.53)	4.86	(2.31)	5.21	(1.72)
skinhead	2200	3.54	(2.25)	4.62	(2.47)	4.19	(1.36)
skip	2201	6.15	(1.51)	5.70	(2)	5.37	(1.92)
skit	2202	6.44	(1.76)	5.22	(2.26)	5.89	(1.83)
skull	608	4.27	(1.83)	4.75	(1.85)	4.86	(1.62)
sky	572	7.37	(1.4)	4.27	(2.17)	5.16	(2)
skyline	2203	6.90	(1.74)	5.28	(2.03)	5.31	(1.97)
skyscraper	573	5.88	(1.87)	5.71	(2.17)	4.33	(2.36)
slang	2204	4.67	(1.49)	4.80	(1.71)	5.23	(1.63)
slap	396	2.95	(1.79)	6.46	(2.58)	4.21	(2.29)
slaughter	397	1.64	(1.18)	6.77	(2.42)	3.82	(2.75)
slave	398	1.84	(1.13)	6.21	(2.93)	3.29	(2.76)
slayer	2205	4.00	(2.38)	6.24	(2.2)	4.76	(2.65)
sleep	399	7.20	(1.77)	2.80	(2.66)	5.41	(2.41)
slim	2206	6.97	(1.43)	5.62	(1.72)	5.93	(1.91)
slime	400	2.68	(1.66)	5.36	(2.63)	4.17	(1.82)
slip	2207	3.59	(1.82)	5.46	(2.43)	3.14	(1.92)
slob	2208	3.12	(1.77)	4.23	(1.8)	4.27	(1.87)
slogan	2209	5.83	(1.39)	4.18	(2.07)	5.14	(0.8)
slouch	2210	4.07	(1.26)	3.83	(1.89)	4.83	(1.83)
slow	982	3.93	(1.6)	3.39	(2.22)	4.35	(1.61)
sludge	2211	4.00	(1.77)	3.80	(1.69)	4.77	(1.61)
slum	401	2.39	(1.25)	4.78	(2.52)	3.83	(2.18)
slush	983	4.66	(1.88)	3.73	(2.23)	4.91	(1.48)
smack	2212	3.50	(1.71)	5.64	(2.3)	4.57	(2.53)
smallpox	402	2.52	(2.08)	5.58	(2.13)	4.29	(2.17)

Description	Word No.	Valence Mean(SD)		Arousal Mean(SD)		Dominance Mean (SD)	
smart	2213	7.93	(1.48)	5.90	(2.22)	6.77	(2.18)
smear	2214	3.92	(1.62)	5.15	(1.78)	4.46	(1.61)
smell	2215	5.20	(1.83)	5.20	(1.89)	5.44	(1.8)
smile	2216	8.16	(1.77)	5.93	(2.64)	6.60	(2.22)
smirk	2217	5.52	(1.84)	4.97	(1.76)	5.55	(1.62)
smoke	2218	3.39	(3.02)	4.48	(2.87)	4.67	(2.48)
smooth	984	6.58	(1.78)	4.91	(2.57)	5.09	(2.09)
smudge	2219	4.00	(1.34)	4.43	(1.67)	4.46	(1.32)
snail	2220	4.31	(1.67)	3.86	(2.27)	5.68	(2.04)
snake	609	3.31	(2.2)	6.82	(2.1)	3.78	(2.05)
snare	2221	4.48	(2.12)	5.41	(1.82)	4.78	(2.03)
snatch	2222	4.66	(2.18)	5.47	(2.16)	4.53	(2.05)
sneakers	2223	6.34	(1.59)	5.07	(2.31)	6.36	(1.5)
sneer	2224	3.76	(1.55)	4.93	(2.27)	4.66	(1.65)
sneeze	2225	4.76	(1.77)	4.96	(2.03)	3.93	(1.68)
snob	403	3.36	(1.81)	5.65	(2.36)	5.11	(2.25)
snow	575	7.08	(1.83)	5.75	(2.47)	5.80	(1.97)
snuggle	404	7.92	(1.24)	4.16	(2.8)	5.66	(2.47)
soak	2226	5.38	(1.97)	5.36	(2.51)	5.11	(1.55)
soap	2227	5.97	(1.64)	4.52	(1.64)	5.97	(1.38)
soar	2228	6.90	(1.63)	6.17	(2.25)	5.83	(2.21)
sob	2229	2.03	(1.32)	5.86	(2.32)	3.36	(1.5)
soccer	2230	5.90	(1.95)	5.39	(2.3)	5.61	(1.73)
social	985	6.88	(1.82)	4.98	(2.59)	5.91	(2.07)
society	2231	5.03	(1.3)	4.82	(2.44)	4.39	(2.31)
sock	2232	5.04	(1.14)	3.46	(2.12)	5.57	(1.53)
soda	2233	6.50	(2.08)	5.77	(2.05)	5.83	(1.95)
sodomize	2234	3.70	(1.97)	5.13	(2.39)	3.80	(2.02)
sofa	2235	6.53	(1.5)	3.10	(1.92)	6.17	(1.56)
soft	986	7.12	(1.34)	4.63	(2.61)	6.00	(1.8)
soil	2236	4.67	(1.47)	3.57	(1.76)	4.70	(1.95)
soldier	2237	5.79	(2.02)	5.48	(2.1)	5.69	(1.95)
solemn	405	4.32	(1.51)	3.56	(1.95)	4.61	(1.87)
son	2238	6.39	(1.52)	5.71	(1.8)	5.64	(1.79)
song	987	7.10	(1.97)	6.07	(2.42)	5.85	(2.12)
soothe	2239	7.58	(1.24)	4.96	(2.62)	6.00	(1.26)
soothe	988	7.30	(1.85)	4.40	(3.08)	5.36	(2.24)
sorrow	2240	2.32	(1.68)	4.48	(2.38)	3.67	(2.18)
sorry	2241	3.62	(2.29)	5.11	(2.35)	3.82	(1.98)
soul	2242	6.72	(1.33)	4.39	(1.85)	5.71	(1.76)
soup	2243	6.25	(1.94)	4.32	(2.09)	5.61	(1.85)
sour	989	3.93	(1.98)	5.10	(1.95)	4.64	(1.5)
spa	2244	7.86	(1.27)	4.82	(2.92)	5.46	(1.95)
space	574	6.78	(1.66)	5.14	(2.54)	5.20	(2.44)
spanking	990	3.55	(2.54)	5.41	(2.73)	3.91	(2.51)
speech	2245	4.54	(2.17)	5.68	(2.33)	4.86	(2.73)
spend	2246	5.60	(2.47)	5.60	(1.87)	5.90	(1.97)
sphere	991	5.33	(0.87)	3.88	(1.99)	5.00	(0.92)
spice	2247	6.21	(1.74)	5.62	(2.34)	5.62	(1.5)
spider	610	3.33	(1.72)	5.71	(2.21)	4.75	(2.11)
spike	2248	5.34	(1.14)	4.96	(1.62)	5.11	(1.26)
spine	2249	5.12	(1.13)	4.48	(2.06)	5.32	(1.46)
spirit	406	7.00	(1.32)	5.56	(2.62)	5.82	(2.42)
sponge	2250	5.11	(1.15)	3.52	(1.95)	4.96	(1.76)
spoon	2251	5.93	(1.39)	4.15	(2.13)	5.37	(1.67)
sports	2252	6.45	(1.95)	6.23	(2.31)	5.77	(2.13)
spouse	407	7.58	(1.48)	5.21	(2.75)	5.53	(1.97)
spray	992	5.45	(1.63)	4.14	(2.28)	5.12	(1.43)
spring	993	7.76	(1.51)	5.67	(2.51)	6.26	(1.98)
spur	2253	5.17	(2.17)	5.07	(2.12)	5.50	(1.55)
spurn	2254	4.38	(1.54)	4.38	(2.11)	4.41	(1.52)
squad	2255	5.58	(1.42)	5.19	(1.88)	4.77	(1.82)
square	408	4.74	(1.02)	3.18	(1.76)	4.51	(1.45)
squash	2256	4.97	(1.79)	3.93	(2.12)	5.48	(1.88)
squeal	2257	4.67	(2.53)	6.26	(2.41)	4.52	(1.95)
stagnant	994	4.15	(1.57)	3.93	(1.94)	4.71	(1.36)
stairs	2258	4.67	(1.45)	5.00	(1.64)	4.87	(1.87)
stalk	2259	2.80	(1.65)	5.41	(2.37)	3.62	(2.11)
star	409	7.27	(1.66)	5.83	(2.44)	4.68	(2.15)
startled	410	4.50	(1.67)	6.93	(2.24)	4.48	(1.57)

Description	Word No.	Valence Mean(SD)		Arousal Mean(SD)		Dominance Mean (SD)	
starvation	2260	1.82	(1.68)	6.46	(2.47)	3.11	(2.17)
starve	2261	2.50	(1.7)	4.93	(2.32)	3.93	(2.27)
starving	611	2.39	(1.82)	5.61	(2.53)	3.63	(2.1)
statue	995	5.17	(0.7)	3.46	(1.72)	4.95	(1.4)
status	2262	5.62	(1.27)	4.93	(1.79)	5.41	(1.59)
steak	2263	6.86	(2.46)	5.32	(2.55)	6.18	(2.02)
steal	2264	3.18	(1.91)	5.11	(2.51)	4.93	(2.42)
steam	2265	5.00	(1.13)	4.35	(2.3)	5.15	(0.78)
stench	996	2.19	(1.37)	4.36	(2.46)	4.29	(1.91)
stereo	2266	6.89	(1.47)	6.04	(2.19)	6.07	(1.54)
stiff	997	4.68	(1.97)	4.02	(2.41)	4.93	(2.04)
stifle	2267	3.50	(1.5)	4.93	(1.98)	3.71	(1.88)
sting	2268	2.48	(1.76)	6.78	(2.59)	3.67	(2.47)
stingy	2269	3.10	(1.6)	5.37	(1.96)	4.53	(1.89)
stink	411	3.00	(1.79)	4.26	(2.1)	4.16	(1.98)
stolen	2270	2.69	(1.51)	6.03	(2.11)	3.41	(1.92)
stomach	998	4.82	(2.06)	3.93	(2.49)	4.68	(1.85)
stool	999	4.56	(1.72)	4.00	(2.14)	4.98	(1.85)
stop	2271	3.96	(1.37)	4.26	(2.01)	4.89	(2.01)
store	2272	5.93	(1.31)	5.00	(2.04)	6.00	(1.46)
storm	1000	4.95	(2.22)	5.71	(2.34)	4.54	(2.04)
story	2273	6.63	(1.85)	5.37	(2.2)	5.47	(1.25)
stove	1001	4.98	(1.69)	4.51	(2.14)	5.36	(1.87)
stow	2274	5.23	(0.67)	3.74	(1.71)	5.19	(1.49)
street	412	5.22	(0.72)	3.39	(1.87)	4.81	(1.21)
strength	2275	7.41	(1.5)	5.76	(1.7)	7.21	(1.61)
stress	413	2.09	(1.41)	7.45	(2.38)	3.93	(2.75)
stricken	2276	3.29	(1.82)	6.04	(2.1)	3.14	(1.6)
stride	2277	5.72	(1.31)	4.75	(1.87)	6.38	(1.47)
string	2278	5.34	(1.76)	4.36	(2.33)	5.57	(1.64)
strive	2279	6.78	(1.34)	6.19	(2.02)	6.19	(1.75)
strong	414	7.11	(1.48)	5.92	(2.28)	6.92	(2.43)
structure	2280	5.21	(1.42)	4.04	(1.85)	5.41	(1.55)
stubborn	2281	3.86	(1.94)	5.30	(2.4)	5.33	(2.25)
studio	2282	6.03	(1.45)	5.11	(1.65)	5.85	(1.56)
stun	2283	4.93	(1.77)	6.29	(2.05)	3.66	(1.93)
stupid	415	2.31	(1.37)	4.72	(2.71)	2.98	(2.18)
stupor	2284	4.39	(1.99)	4.48	(2.06)	3.67	(2.04)
style	2285	6.82	(1.63)	5.36	(2.08)	5.64	(1.85)
subdue	2286	4.69	(1.65)	4.30	(2.05)	4.56	(2.06)
subdued	416	4.67	(1.31)	2.90	(1.81)	4.08	(1.56)
subject	2287	5.04	(0.79)	3.93	(1.82)	4.68	(1.68)
submit	2288	4.37	(1.86)	5.22	(1.95)	3.96	(2.21)
suburb	2289	5.89	(1.43)	4.54	(1.86)	5.84	(1.07)
subway	2290	6.29	(1.72)	5.52	(2.39)	5.37	(2.17)
success	417	8.29	(0.93)	6.11	(2.65)	6.89	(2.4)
suffer	2291	1.72	(1.34)	6.12	(2.89)	2.54	(2.13)
suffocate	418	1.56	(0.96)	6.03	(3.19)	3.44	(2.81)
sugar	1002	6.74	(1.73)	5.64	(2.18)	5.50	(1.5)
suggestion	2292	5.66	(1.04)	4.72	(1.55)	4.88	(1.31)
suicide	419	1.25	(0.69)	5.73	(3.14)	3.58	(3.02)
suite	2293	7.25	(1.78)	5.59	(2.55)	5.44	(1.99)
sun	532	7.55	(1.85)	5.04	(2.66)	6.16	(2.09)
sunflower	2294	7.41	(1.34)	4.30	(2.2)	5.33	(2.11)
sunglasses	2295	6.47	(1.43)	4.90	(2.37)	5.97	(1.43)
sunlight	1003	7.76	(1.43)	6.10	(2.3)	5.63	(2.15)
sunrise	420	7.86	(1.35)	5.06	(3.05)	5.29	(2.41)
sunset	421	7.68	(1.72)	4.20	(2.99)	5.66	(2.08)
supper	2296	7.23	(1.55)	6.30	(2.18)	6.03	(1.77)
supply	2297	5.27	(1.12)	4.38	(1.7)	5.27	(1.31)
sure	2298	6.53	(1.96)	5.00	(2.39)	6.03	(2.16)
surfer	2299	6.55	(1.86)	5.45	(2.25)	5.45	(1.62)
surgeon	2300	5.36	(1.87)	5.86	(2.22)	4.61	(2.1)
surgery	612	2.86	(2.19)	6.35	(2.32)	2.75	(1.86)
surprise	2301	7.73	(1.66)	7.07	(1.95)	3.87	(2.22)
surprised	422	7.47	(1.56)	7.47	(2.09)	6.11	(2.19)
sushi	2302	5.72	(2.67)	5.22	(2.21)	5.81	(2.09)
suspicious	423	3.76	(1.42)	6.25	(1.59)	4.47	(1.99)
swamp	1004	5.14	(2.24)	4.86	(2.36)	5.29	(1.63)
swan	2303	6.48	(2.01)	3.52	(2.16)	5.32	(1.87)

Description	Word No.	Valence Mean(SD)		Arousal Mean(SD)		Dominance Mean (SD)	
swear	2304	4.43	(1.45)	5.46	(2.05)	4.79	(1.79)
sweet	2305	7.64	(1.42)	5.96	(2.5)	5.36	(2.02)
sweetheart	424	8.42	(0.83)	5.50	(2.73)	6.03	(2.24)
swift	1005	6.46	(1.76)	5.39	(2.53)	6.29	(1.85)
swimmer	576	6.54	(1.64)	4.82	(2.49)	5.96	(1.91)
swing	2306	7.20	(1.49)	5.53	(2.13)	5.33	(1.81)
swipe	2307	4.86	(1.46)	4.33	(1.82)	5.11	(1.97)
sword	2308	5.52	(2.14)	6.08	(1.82)	5.62	(1.88)
sympathy	2309	5.33	(1.97)	5.03	(1.96)	4.73	(1.7)
syphilis	425	1.68	(1.23)	5.69	(3.25)	3.33	(2.67)
syrup	2310	6.07	(1.28)	4.69	(2.16)	5.86	(1.3)
system	2311	5.37	(1.3)	3.93	(1.91)	5.37	(1.88)
table	426	5.22	(0.72)	2.92	(2.16)	4.47	(1.66)
taint	2312	3.92	(1.65)	4.85	(1.71)	4.35	(1.7)
tale	2313	6.43	(1.89)	4.13	(2)	5.50	(2.01)
talent	427	7.56	(1.25)	6.27	(1.8)	6.49	(1.75)
tall	2314	6.64	(1.85)	4.82	(2.6)	5.57	(2.57)
tamper	1006	4.10	(1.88)	4.95	(2.01)	4.58	(2.1)
tangle	2315	3.97	(1.45)	5.66	(1.79)	4.66	(1.54)
tank	613	5.16	(1.87)	4.88	(1.86)	4.78	(1.93)
tar	2316	4.31	(1.46)	4.46	(1.58)	4.50	(1.88)
task	2317	4.79	(1.52)	4.72	(1.65)	5.93	(2.03)
taste	1007	6.66	(1.57)	5.22	(2.38)	5.50	(1.65)
tatter	2318	4.53	(1.7)	3.90	(1.93)	4.50	(1.23)
tattoo	2319	5.62	(2.11)	6.06	(1.88)	5.69	(1.99)
taunt	2320	3.61	(1.71)	5.55	(1.8)	4.90	(1.97)
tavern	2321	5.81	(1.42)	5.00	(1.98)	5.48	(1.29)
taxi	1008	5.00	(1.96)	3.41	(2.14)	4.64	(1.83)
tea	2322	5.87	(2.39)	4.13	(2.26)	5.71	(1.96)
teach	2323	5.89	(1.95)	5.59	(1.89)	5.88	(2.3)
teacher	1009	5.68	(2.12)	4.05	(2.61)	5.11	(2.2)
team	2324	7.43	(1.3)	5.93	(2.23)	5.90	(2.23)
tease	1010	4.84	(2.51)	5.87	(2.56)	4.67	(2.37)
teenager	2325	6.41	(2)	6.33	(2.13)	4.67	(2.25)
teeter	2326	5.18	(1.72)	5.30	(2.18)	4.56	(2.22)

Description	Word No.	Valence Mean(SD)		Arousal Mean(SD)		Dominance Mean (SD)	
teeth	2327	5.46	(1.36)	4.77	(2.29)	4.85	(2.24)
tempo	2328	5.89	(1.74)	5.04	(2.25)	5.96	(1.66)
temptation	2329	4.94	(1.98)	5.88	(1.96)	4.50	(2.26)
tend	2330	5.55	(1.62)	3.93	(2.46)	5.43	(2.35)
tender	1011	6.93	(1.28)	4.88	(2.3)	5.33	(1.75)
tendon	2331	4.90	(1.22)	4.90	(2.22)	4.83	(2.2)
tennis	540	6.02	(1.97)	4.61	(2.6)	5.61	(2.12)
tense	428	3.56	(1.36)	6.53	(2.1)	5.22	(2.02)
termite	429	3.58	(2.08)	5.39	(2.43)	3.87	(1.87)
terrace	2332	5.55	(1.5)	4.71	(1.76)	5.00	(1.59)
terrible	430	1.93	(1.44)	6.27	(2.44)	3.58	(2.34)
terrific	431	8.16	(1.12)	6.23	(2.73)	6.60	(2.15)
terrified	432	1.72	(1.14)	7.86	(2.27)	3.08	(2.75)
terror	2333	2.75	(2.2)	7.07	(2.32)	2.71	(2.16)
terrorist	614	1.69	(1.42)	7.27	(2.38)	2.65	(2.3)
testicles	2334	5.38	(1.1)	5.25	(1.92)	5.81	(1.51)
thankful	433	6.89	(2.29)	4.34	(2.31)	5.32	(2)
theater	2335	6.69	(1.42)	5.86	(1.57)	5.45	(1.48)
theft	2336	2.67	(1.49)	5.96	(2.56)	3.27	(2.07)
theory	434	5.30	(1.49)	4.62	(1.94)	4.88	(1.81)
therapy	2337	4.42	(2.25)	4.57	(2.29)	4.03	(2.33)
thermometer	1012	4.73	(1.05)	3.79	(2.02)	4.39	(1.51)
thief	435	2.13	(1.69)	6.89	(2.13)	3.79	(2.55)
thigh	2338	5.77	(1.36)	5.47	(1.98)	5.60	(1.43)
thin	2339	6.43	(1.71)	5.57	(2.08)	5.36	(2.16)
thing	2340	4.83	(0.83)	4.17	(1.72)	4.77	(1.28)
think	2341	6.41	(1.88)	5.34	(2.48)	6.38	(2.19)
thirst	2342	3.37	(1.81)	5.03	(1.97)	3.93	(1.95)
thirsty	2343	3.61	(1.63)	5.67	(1.45)	3.77	(1.36)
thong	2344	6.69	(1.99)	5.78	(2.52)	6.69	(1.69)
thorn	436	3.64	(1.76)	5.14	(2.14)	4.45	(1.5)
thought	1013	6.39	(1.58)	4.83	(2.46)	6.02	(1.7)
thoughtful	437	7.65	(1.03)	5.72	(2.3)	5.61	(2.11)
threat	2345	2.50	(1.21)	6.08	(2.48)	3.27	(2.03)
thrift	2346	4.90	(1.73)	4.43	(1.72)	5.23	(1.36)

Description	Word No.	Valence Mean(SD)		Arousal Mean(SD)		Dominance Mean (SD)	
thrill	438	8.05	(1.48)	8.02	(1.65)	6.54	(2.3)
thrilled	2347	7.81	(1.19)	7.47	(1.72)	6.13	(1.25)
thrive	2348	7.45	(1.57)	6.03	(2.24)	6.29	(1.76)
thud	2349	4.47	(0.97)	5.20	(1.35)	4.87	(0.97)
thug	2350	3.86	(1.63)	5.32	(2.02)	3.96	(2.01)
thumb	2351	5.52	(1.24)	4.54	(1.91)	5.25	(1.69)
tick	2352	3.72	(1.56)	4.14	(2.27)	4.18	(1.61)
ticket	2353	5.46	(2.56)	5.79	(2.59)	5.14	(2.26)
tickle	2354	6.86	(2.14)	6.70	(2.16)	4.07	(2.37)
tidy	1014	6.30	(1.56)	3.98	(2.22)	5.49	(1.93)
tiger	2355	5.89	(2.45)	6.41	(2.42)	3.44	(2.15)
time	439	5.31	(2.02)	4.64	(2.75)	4.63	(2.24)
timid	440	3.86	(1.55)	4.11	(2.09)	3.09	(1.91)
tire	2356	4.97	(1.5)	4.00	(2)	5.03	(1.12)
tired	2357	3.28	(1.69)	2.64	(1.5)	3.96	(2.1)
tissue	2358	4.93	(1.62)	3.63	(2.11)	5.07	(1.8)
title	2359	5.80	(1.81)	4.93	(1.84)	5.89	(2.15)
toad	2360	4.59	(1.68)	4.76	(2.34)	4.45	(1.82)
tobacco	441	3.28	(2.16)	4.83	(2.9)	4.08	(2.27)
toddler	2361	6.97	(1.54)	5.68	(2.01)	6.55	(1.88)
toil	2362	4.20	(1.63)	5.04	(1.77)	4.56	(1.94)
toilet	2363	4.66	(1.8)	3.96	(1.95)	5.26	(1.02)
tomato	2364	5.45	(1.97)	3.79	(2.02)	5.36	(1.52)
tomb	442	2.94	(1.88)	4.73	(2.72)	3.72	(2.05)
ton	2365	4.68	(1.47)	4.33	(1.98)	5.04	(2.17)
tool	1015	5.19	(1.27)	4.33	(1.78)	5.67	(1.62)
tooth	2366	5.19	(1.26)	4.66	(1.98)	5.06	(1.39)
toothache	443	1.98	(1.15)	5.55	(2.51)	3.90	(1.85)
topless	2367	6.93	(2.12)	6.67	(2.4)	6.30	(1.97)
topple	2368	3.93	(1.9)	5.46	(2.33)	4.25	(2.3)
tornado	444	2.55	(1.78)	6.83	(2.49)	4.30	(2.42)
torte	2369	5.33	(1.09)	4.30	(1.74)	4.97	(1.03)
torture	445	1.56	(0.79)	6.10	(2.77)	3.33	(2.37)
total	2370	5.74	(1.63)	4.81	(2.22)	5.30	(1.64)
touch	2371	6.31	(1.49)	6.19	(2.08)	5.81	(1.67)

Description	Word No.	Valence Mean(SD)		Arousal Mean(SD)		Dominance Mean (SD)	
tourist	2372	5.66	(1.59)	4.52	(1.77)	5.38	(1.45)
tow	2373	3.63	(1.63)	4.67	(2.29)	4.00	(2.26)
towel	2374	5.75	(1.55)	4.19	(2.47)	5.69	(1.65)
tower	1016	5.46	(1.75)	3.95	(2.28)	5.78	(2.14)
town	2375	5.93	(1.61)	4.29	(2.31)	4.64	(1.73)
toxic	446	2.10	(1.48)	6.40	(2.41)	4.42	(2.51)
toy	1017	7.00	(2.01)	5.11	(2.84)	6.09	(1.84)
traffic	2376	2.27	(1.31)	5.80	(2.52)	2.57	(1.68)
tragedy	447	1.78	(1.31)	6.24	(2.64)	3.50	(2.34)
train	2377	5.59	(1.25)	4.85	(2.36)	5.04	(1.87)
trait	2378	6.04	(1.81)	4.04	(2.53)	4.07	(2.07)
traitor	448	2.22	(1.69)	5.78	(2.47)	4.61	(2.71)
trash	615	2.67	(1.45)	4.16	(2.16)	5.24	(1.85)
trauma	616	2.10	(1.49)	6.33	(2.45)	2.84	(1.87)
travel	1018	7.10	(2)	6.21	(2.51)	6.31	(2.08)
tray	2379	5.10	(1.03)	3.67	(1.77)	5.17	(1.21)
tread	2380	5.31	(1.31)	4.97	(1.99)	4.86	(1.53)
treasure	449	8.27	(0.9)	6.75	(2.3)	6.36	(2.42)
treat	1019	7.36	(1.38)	5.62	(2.25)	5.78	(1.82)
treatment	2381	5.24	(2.13)	5.52	(1.7)	4.48	(1.82)
tree	450	6.32	(1.56)	3.42	(2.21)	5.08	(2.29)
tremor	2382	3.94	(1.22)	5.41	(2.17)	3.34	(1.79)
trend	2383	5.40	(2.03)	5.03	(2.01)	4.40	(1.87)
tribe	2384	5.61	(1.77)	4.68	(2.33)	5.00	(1.96)
trick	2385	4.97	(2.5)	6.10	(1.73)	4.60	(1.99)
trip	2386	6.96	(1.74)	6.30	(2.05)	5.00	(2.08)
triumph	451	7.80	(1.83)	5.78	(2.6)	6.98	(2.2)
triumphant	452	8.82	(0.73)	6.78	(2.58)	6.95	(2.55)
trivia	2387	5.77	(1.66)	5.00	(2.32)	5.15	(1.8)
trophy	453	7.78	(1.22)	5.39	(2.44)	6.44	(2.32)
trouble	454	3.03	(2.09)	6.85	(2.03)	4.85	(2.39)
troubled	455	2.17	(1.21)	5.94	(2.36)	3.91	(2.33)
trout	2388	5.57	(1.57)	4.40	(2.09)	5.67	(2.09)
truce	2389	6.62	(1.45)	4.82	(2.06)	6.11	(1.03)
truck	577	5.47	(1.88)	4.84	(2.17)	5.33	(1.83)

Description	Word No.	Valence Mean(SD)		Arousal Mean(SD)		Dominance Mean (SD)	
true	2390	6.96	(1.32)	5.11	(2.06)	6.00	(1.39)
trumpet	456	5.75	(1.38)	4.97	(2.13)	4.57	(1.72)
trunk	1020	5.09	(1.57)	4.18	(2.19)	5.14	(1.9)
trust	457	6.68	(2.71)	5.30	(2.66)	6.61	(2.04)
truth	458	7.80	(1.29)	5.00	(2.77)	6.47	(2.11)
tulip	2391	6.83	(1.81)	4.52	(2.37)	5.69	(2.19)
tumor	459	2.36	(2.04)	6.51	(2.85)	3.58	(2.42)
tune	1021	6.93	(1.47)	4.71	(2.09)	5.74	(1.82)
turkey	2392	6.48	(1.92)	5.04	(2.54)	6.12	(1.62)
turmoil	2393	2.71	(2.04)	5.87	(2.53)	3.20	(2.16)
turtle	2394	6.78	(1.53)	4.85	(2.3)	5.07	(1.96)
tusk	2395	5.41	(1.43)	4.46	(2.05)	4.93	(1.46)
twelve	2396	5.24	(1.43)	3.66	(2.16)	5.31	(1.73)
twilight	1022	7.23	(1.8)	4.70	(2.41)	5.59	(1.82)
twins	2397	6.24	(1.33)	5.17	(1.93)	5.21	(1.72)
tyrant	2398	3.25	(2.05)	5.71	(2.35)	3.14	(2.35)
ugly	460	2.43	(1.27)	5.38	(2.23)	4.26	(2.33)
ulcer	461	1.78	(1.17)	6.12	(2.68)	4.17	(2.22)
umbrella	578	5.16	(1.57)	3.68	(1.99)	5.42	(1.91)
uncertain	2399	3.45	(1.43)	4.79	(2.6)	3.37	(1.9)
uncomfortable	2400	2.97	(1.6)	6.06	(1.98)	3.42	(1.77)
understand	2401	7.13	(1.89)	5.00	(2.08)	6.77	(2.03)
uneasy	2402	3.07	(1.91)	6.03	(2.29)	3.24	(1.79)
unfaithful	462	2.05	(1.55)	6.20	(2.7)	3.02	(2.54)
unhappy	463	1.57	(0.96)	4.18	(2.5)	3.34	(2.35)
unify	2403	6.90	(2.06)	4.93	(2.53)	5.83	(2.36)
union	2404	6.48	(1.66)	4.79	(1.88)	6.14	(1.55)
unit	1023	5.59	(1.87)	3.75	(2.49)	5.11	(1.74)
unite	2405	6.77	(1.59)	5.81	(1.96)	6.10	(1.54)
unity	2406	7.29	(1.44)	4.64	(2.68)	6.43	(1.73)
unsafe	2407	2.82	(1.81)	6.18	(2.64)	3.67	(2.54)
unsure	2408	3.03	(1.52)	5.97	(1.99)	3.60	(2.03)
untroubled	464	7.62	(1.41)	3.89	(2.54)	5.53	(2.54)
upset	465	2.00	(1.18)	5.86	(2.4)	4.08	(2.31)
urinate	2409	5.59	(1.58)	4.10	(2.34)	5.45	(2.41)

Description	Word No.	Valence Mean(SD)		Arousal Mean(SD)		Dominance Mean (SD)	
urine	617	3.25	(1.71)	4.20	(2.18)	5.24	(1.86)
usage	2410	4.93	(1.13)	4.90	(1.57)	4.97	(1.3)
useful	466	7.14	(1.6)	4.26	(2.47)	5.93	(2.1)
useless	467	2.13	(1.42)	4.87	(2.58)	3.92	(2.62)
utensil	1024	5.14	(1.39)	3.57	(1.98)	5.40	(1.47)
vacation	468	8.16	(1.36)	5.64	(2.99)	6.80	(2.08)
vagina	1025	6.14	(1.77)	5.55	(2.55)	5.88	(1.74)
valentine	469	8.11	(1.35)	6.06	(2.91)	5.81	(2.45)
valley	2411	5.46	(1.55)	3.70	(1.88)	4.74	(1.85)
vampire	470	4.26	(1.86)	6.37	(2.35)	5.05	(2.27)
van	2412	4.97	(1.77)	3.86	(2.42)	5.03	(1.52)
vandal	471	2.71	(1.91)	6.40	(1.88)	3.91	(2.49)
vanish	2413	3.83	(2.28)	5.29	(2.32)	3.18	(2.07)
vanity	472	4.30	(1.91)	4.98	(2.31)	4.80	(2.03)
vase	2414	4.97	(1.18)	3.90	(2.14)	5.41	(1.52)
vegetable	2415	5.40	(2.06)	4.07	(2.16)	5.27	(2)
vehicle	473	6.27	(2.34)	4.63	(2.81)	5.77	(2.61)
venom	474	2.68	(1.81)	6.08	(2.44)	3.94	(2.23)
vessel	2416	5.50	(1.68)	4.31	(1.81)	5.34	(1.8)
vest	1026	5.25	(1.33)	3.95	(2.09)	5.09	(1.24)
vibrator	2417	6.17	(2.36)	6.39	(2.22)	5.54	(2.2)
vice	2418	4.43	(2.25)	5.30	(2.1)	5.23	(2.18)
victim	618	2.18	(1.48)	6.06	(2.32)	2.69	(2.04)
victory	475	8.32	(1.16)	6.63	(2.84)	7.26	(2.14)
video	2419	7.30	(1.42)	5.67	(2.15)	5.77	(1.45)
vigor	2420	5.85	(1.78)	5.77	(1.97)	5.73	(1.64)
vigorous	476	6.79	(1.54)	5.90	(2.66)	5.41	(2.22)
village	477	5.92	(1.34)	4.08	(1.87)	4.94	(1.74)
vine	2421	5.36	(1.64)	4.48	(2.17)	4.75	(1.9)
violent	478	2.29	(1.78)	6.89	(2.47)	5.16	(2.86)
violin	579	5.43	(1.98)	3.49	(2.26)	5.18	(2.01)
violinist	2422	6.32	(1.57)	4.33	(2.24)	5.38	(1.93)
virgin	1027	6.45	(1.76)	5.51	(2.06)	6.24	(2.48)
virtue	479	6.22	(2.06)	4.52	(2.52)	6.13	(2.09)
virus	2423	2.50	(1.75)	5.46	(2.66)	2.79	(1.55)

Description	Word No.	Valence Mean(SD)		Arousal Mean(SD)		Dominance Mean (SD)	
vision	480	6.62	(1.84)	4.66	(2.43)	6.02	(1.96)
vista	2424	5.10	(1.81)	4.57	(2.03)	4.63	(1.5)
vodka	2425	5.52	(2.34)	5.86	(2.37)	5.00	(2.05)
volcano	619	4.84	(2.14)	6.33	(2.21)	3.25	(1.97)
vomit	481	2.06	(1.57)	5.75	(2.84)	3.58	(2.45)
vote	2426	6.63	(1.59)	5.30	(2.22)	6.93	(2.07)
vow	2427	6.08	(2.04)	5.35	(1.98)	5.92	(2)
vows	2428	6.48	(1.79)	6.13	(1.71)	5.61	(1.75)
voyage	1028	6.25	(1.91)	5.55	(2.23)	5.18	(1.98)
wagon	1029	5.37	(0.97)	3.98	(2.04)	5.05	(1.2)
wail	2429	3.63	(1.94)	5.50	(1.63)	4.53	(1.57)
wall	2430	4.76	(1.21)	4.18	(1.87)	4.41	(1.78)
wander	2431	5.27	(1.6)	4.93	(1.72)	5.43	(2.16)
wane	2432	4.39	(1.81)	4.54	(2.01)	3.96	(1.71)
want	2433	5.25	(2.08)	6.00	(2.11)	4.54	(2.13)
war	482	2.08	(1.91)	7.49	(2.16)	4.50	(3)
warden	2434	3.79	(1.68)	4.24	(2.01)	3.41	(1.88)
warehouse	2435	5.13	(1.55)	4.00	(1.97)	4.72	(1.51)
warm	2436	6.50	(1.2)	5.14	(1.92)	5.36	(1.25)
warmth	483	7.41	(1.81)	3.73	(2.4)	5.61	(1.67)
warn	2437	4.20	(1.52)	5.37	(2.19)	4.60	(1.77)
wart	2438	2.41	(1.36)	4.34	(2.5)	3.62	(1.52)
wasp	484	3.37	(1.63)	5.50	(2.17)	3.76	(1.82)
waste	485	2.93	(1.76)	4.14	(2.3)	4.72	(1.94)
watch	580	5.78	(1.51)	4.10	(2.12)	5.37	(1.75)
water	486	6.61	(1.78)	4.97	(2.49)	5.08	(1.99)
waterfall	487	7.88	(1.03)	5.37	(2.84)	5.20	(2.18)
watermelon	2439	6.52	(1.64)	5.18	(1.74)	5.79	(1.34)
waterskiing	2440	7.00	(2.19)	6.77	(2.18)	5.60	(2.53)
wealth	2441	7.70	(1.39)	5.90	(2.11)	6.70	(2.25)
wealthy	488	7.70	(1.34)	5.80	(2.73)	6.77	(2.57)
weapon	489	3.97	(1.92)	6.03	(1.89)	5.19	(2.61)
weary	490	3.79	(2.12)	3.81	(2.29)	4.00	(1.91)
web	2442	5.33	(1.69)	4.30	(2.38)	5.20	(1.47)
wed	2443	6.75	(1.96)	6.41	(2.26)	5.56	(2.33)

Description	Word No.	Valence Mean(SD)		Arousal Mean(SD)		Dominance Mean (SD)	
wedding	491	7.82	(1.56)	5.97	(2.85)	6.68	(2.08)
week	2444	5.35	(1.02)	4.38	(1.7)	4.92	(1.41)
weep	2445	1.80	(1.24)	5.07	(2.19)	3.00	(1.79)
weight	2446	4.28	(1.74)	5.40	(1.96)	4.92	(2.55)
weightlifter	2447	5.93	(1.7)	5.50	(2.08)	5.57	(2.11)
welfare	2448	3.36	(2.13)	5.18	(2.54)	2.82	(1.63)
wet	2449	5.57	(1.76)	5.57	(2.08)	5.63	(1.67)
wheat	2450	5.90	(1.32)	4.03	(2.08)	5.62	(1.24)
wheel	2451	5.55	(0.83)	4.86	(1.51)	5.55	(1.24)
whistle	1030	5.81	(1.21)	4.69	(1.99)	5.27	(1.87)
white	542	6.47	(1.59)	4.37	(2.14)	5.98	(1.73)
whore	492	2.30	(2.11)	5.85	(2.93)	4.61	(2.73)
wicked	493	2.96	(2.37)	6.09	(2.44)	4.36	(2.65)
wife	1031	6.33	(1.97)	4.93	(2.22)	5.57	(1.68)
wild	2452	6.37	(1.4)	6.43	(1.92)	5.27	(1.89)
willing	2453	6.60	(1.45)	5.30	(1.82)	6.00	(1.53)
win	494	8.38	(0.92)	7.72	(2.16)	7.39	(2.36)
wince	2454	3.85	(1.41)	5.27	(1.8)	4.69	(1.46)
windmill	1032	5.60	(1.65)	3.74	(2.13)	5.24	(1.04)
window	495	5.91	(1.38)	3.97	(2.01)	4.91	(1.6)
windshield	2455	4.64	(0.99)	4.22	(2.03)	4.93	(1.41)
windsurfers	2456	6.50	(1.57)	6.30	(1.47)	5.33	(1.84)
wine	496	5.95	(2.19)	4.78	(2.34)	5.31	(2.15)
wings	2457	6.39	(1.93)	5.33	(2.27)	4.96	(1.95)
wink	1033	6.93	(1.83)	5.44	(2.68)	5.70	(1.77)
winner	2458	8.24	(1.18)	6.86	(2.46)	7.72	(1.77)
winter	2459	5.79	(2.61)	5.32	(2.5)	4.79	(2.04)
wires	2460	4.63	(1.5)	4.70	(2.02)	4.73	(1.57)
wisdom	2461	7.34	(1.45)	5.25	(2.38)	6.61	(1.66)
wise	497	7.52	(1.23)	3.91	(2.64)	6.70	(2.39)
wish	1034	7.09	(2)	5.16	(2.62)	5.28	(2.09)
wishful	2462	7.50	(1.46)	5.57	(2.36)	5.27	(1.89)
wit	1035	7.32	(1.9)	5.42	(2.44)	6.38	(2.01)
wolf	2463	5.00	(2.35)	6.70	(1.82)	4.33	(2.31)
woman	498	6.64	(1.76)	5.32	(2.59)	6.33	(1.52)

Description	Word No.	Valence Mean(SD)		Arousal Mean(SD)		Dominance Mean (SD)	
wonder	499	6.03	(1.58)	5.00	(2.23)	5.32	(2.17)
wood	2464	5.86	(1.84)	4.67	(1.98)	5.59	(1.19)
wool	2465	5.27	(1.14)	3.60	(2.04)	5.17	(1.32)
work	2466	3.96	(2.05)	5.11	(2.28)	4.11	(2.17)
worker	2467	5.07	(1.53)	4.73	(1.95)	5.27	(1.86)
world	500	6.50	(2.03)	5.32	(2.39)	5.26	(2.47)
worry	2468	2.31	(1.51)	6.00	(2.45)	2.96	(2.22)
worth	2469	6.15	(1.35)	4.62	(2.12)	5.23	(1.63)
wounds	620	2.51	(1.58)	5.82	(2.01)	3.92	(1.57)
wrath	2470	3.47	(2.33)	5.60	(2.42)	4.23	(2.53)
wreath	2471	6.07	(1.36)	4.64	(1.75)	5.14	(1.04)
wring	2472	4.81	(0.93)	4.78	(1.52)	5.03	(1.43)
writer	1036	5.52	(1.9)	4.33	(2.45)	4.73	(1.84)
wrong	2473	2.93	(1.8)	4.67	(2.04)	3.30	(1.8)
yacht	1037	6.95	(1.79)	5.61	(2.72)	6.10	(2.13)
year	2474	5.41	(1.41)	4.22	(1.96)	5.25	(1.3)
yellow	545	5.61	(1.94)	4.43	(2.05)	5.47	(1.58)
yelp	2475	3.69	(1.46)	5.73	(1.95)	4.27	(1.69)
yolk	2476	5.48	(1.77)	4.07	(2.14)	5.04	(1.35)
young	1038	6.89	(2.12)	5.64	(2.51)	5.30	(2.49)
youth	1039	6.75	(2.29)	5.67	(2.52)	5.11	(2.55)
zeal	2477	6.15	(1.66)	5.41	(1.6)	5.52	(1.5)
zealous	2478	5.67	(2.02)	5.47	(2.5)	5.60	(1.73)
zest	1040	6.79	(2.04)	5.59	(2.66)	6.00	(1.99)
zipper	2479	5.39	(1.23)	5.19	(1.9)	5.63	(2)
zoom	2480	6.56	(1.83)	5.88	(2.35)	5.91	(1.87)

Description	Word No.	Valence Mean(SD)	Arousal Mean(SD)	Dominance Mean (SD)
